# Chromosome rearrangements shape the diversification of secondary metabolism in the cyclosporin producing fungus *Tolypocladium inflatum*

**DOI:** 10.1186/s12864-018-5399-x

**Published:** 2019-02-07

**Authors:** Rodrigo A. Olarte, Jon Menke, Ying Zhang, Shawn Sullivan, Jason C. Slot, Yinyin Huang, Jonathan P. Badalamenti, Alisha C. Quandt, Joseph W. Spatafora, Kathryn E. Bushley

**Affiliations:** 1 Department of Plant and Microbial Biology, University of Minnesota, St. Paul, MN USA; 2 Cargill Inc., Wayzata, MN USA; 3 Minnesota Supercomputing Institute, Minneapolis, MN USA; 4 Phase Genomics, Seattle, WA USA; 5 Department of Plant Pathology, Ohio State University, Columbus, OH USA; 6 University of Minnesota Genomics Center, University of Minnesota, Minneapolis, MN USA; 7 Department of Ecology and Evolutionary Biology, University of Colorado, Boulder, CO USA; 8 Department of Botany and Plant Pathology, Oregon State University, Corvallis, OR USA

**Keywords:** Fungi, Secondary metabolism, Genome evolution, Structural variation, Chromosome rearrangement, Hi-C

## Abstract

**Background:**

Genes involved in production of secondary metabolites (SMs) in fungi are exceptionally diverse. Even strains of the same species may exhibit differences in metabolite production, a finding that has important implications for drug discovery. Unlike in other eukaryotes, genes producing SMs are often clustered and co-expressed in fungal genomes, but the genetic mechanisms involved in the creation and maintenance of these secondary metabolite biosynthetic gene clusters (SMBGCs) remains poorly understood.

**Results:**

In order to address the role of genome architecture and chromosome scale structural variation in generating diversity of SMBGCs, we generated chromosome scale assemblies of six geographically diverse isolates of the insect pathogenic fungus *Tolypocladium inflatum*, producer of the multi-billion dollar lifesaving immunosuppressant drug cyclosporin, and utilized a Hi-C chromosome conformation capture approach to address the role of genome architecture and structural variation in generating intraspecific diversity in SMBGCs. Our results demonstrate that the exchange of DNA between heterologous chromosomes plays an important role in generating novelty in SMBGCs in fungi. In particular, we demonstrate movement of a polyketide synthase (PKS) and several adjacent genes by translocation to a new chromosome and genomic context, potentially generating a novel PKS cluster. We also provide evidence for inter-chromosomal recombination between nonribosomal peptide synthetases located within subtelomeres and uncover a polymorphic cluster present in only two strains that is closely related to the cluster responsible for biosynthesis of the mycotoxin aflatoxin (AF), a highly carcinogenic compound that is a major public health concern worldwide. In contrast, the cyclosporin cluster, located internally on chromosomes, was conserved across strains, suggesting selective maintenance of this important virulence factor for infection of insects.

**Conclusions:**

This research places the evolution of SMBGCs within the context of whole genome evolution and suggests a role for recombination between chromosomes in generating novel SMBGCs in the medicinal fungus *Tolypocladium inflatum*.

**Electronic supplementary material:**

The online version of this article (10.1186/s12864-018-5399-x) contains supplementary material, which is available to authorized users.

## Background

Gene clusters involved in secondary metabolite (SM) production are exceptionally diverse and contain some of the most rapidly evolving gene families in fungi [1–3]. Even within a single species, strain specific variation in metabolite production can have important phenotypic consequences, such as conferring pathogenicity on specific hosts [4–6]. Yet, few studies have examined the variability in the genetic potential for SM production within species [7–10] or systematically evaluated the roles of genetic processes such as transposition, recombination, gene-conversion, inversion, and duplication/deletion in generating this diversity at the population scale [9, 10]. For most species of fungi, the genome sequence of a single reference strain is used to assess SM potential of an entire species. Clearly, this approach fails to capture a large proportion of diversity in SM potential across fungi and has important implications for drug screening programs and natural products development.

Unlike in other eukaryotes, the genes involved in production of most SMs in fungi are clustered in the genome, forming secondary metabolite biosynthetic gene clusters (SMBGCs) that are also co-regulated and co-expressed [11, 12]. These SMBGCs may contain one or multiple types of “core” enzymes responsible for biosynthesis of the backbone structure of the metabolite. The main classes of these core enzymes include non-ribosomal peptide synthetases (NRPSs), Polyketide synthases (PKSs), terpene synthases (TSs), and dimethylallyl tryptophan synthases (DMATs). Both NRPSs and PKSs are large multi-modular enzymes comprised of repeated units of protein domains. NRPSs, for example, synthesize bioactive peptides in an assembly line fashion. They may contain multiple modules, each consisting of an adenylation (A) domain involved in substrate recognition, a condensation (C) domain, and a thiolation (T) domain, responsible for addition of a single amino acid substrate onto the growing peptide chain [13]. Multimodular NRPSs are thought to evolve through tandem duplication or deletion of these A-T-C modules [14]. In addition to these core enzymes, SMBGCs also harbor “modifying” enzymes, such as dehydrogenases, methyltransferases, acetyl transferases, and cytochrome P450 monooxygenases (P450s), among others, that are involved in chemical transformations of the backbone structure to produce the final metabolite. SMBGCs may also contain cluster specific transcription factors that coordinately regulate expression of all genes in the cluster, as well as transporters involved in detoxification or self-protection in the producing fungus [15].

Various hypotheses have been put forth to explain the evolutionary forces driving clustering of SM genes in fungi, including horizontal gene transfer (HGT) [12], reduced toxicity [16], and coordinated regulation through epigenetic mechanisms [17]. The unique clustering of secondary metabolic pathways in fungi allows for evolution of novel metabolites at both the gene and the cluster level. At the gene level, point or indel null mutations in the core genes or regulatory transcription factors of SMBGCs can inactivate metabolite production [18]. Similarly, mutations in amino acid residues in the A domains involved in substrate recognition in NRPSs [19] or the duplication, loss, or “swapping” of modules [14] can dramatically alter the metabolite backbone structure.

At the cluster level, changes in the gene content of a SMBGC through duplication/loss of individual genes or groups of genes can lead to evolution of novel metabolites [20–22]. Whole cluster duplication [6, 23], loss/degeneration [22, 24, 25], or fragmentation [21] have also been observed. In some cases, distinct SMBGCs have also been shown to occupy the same syntenic ancestral locus [26]. The observation that the same SMBGC locus may contain different modular subunits or “alleles” of a larger composite cluster has recently been described as an allelic or “idiomorphic polymorphism” [10], in reference to the fungal mating type loci, in which unrelated genes occupy the same mating type locus and differentiate the two mating types (Mat-a and Mat-α) [27]. Because of a clear null phenotype, loss of function mutations have generally been better characterized than gain of function mutations and shown to occur relatively frequently in fungal populations [10]. In contrast, only a handful of studies have demonstrated the birth of clustered primary metabolic pathways in fungi, such as the DAL [28] cluster in yeast, among others [16, 29], while no studies have yet documented the birth of a metabolic cluster involved in production of a SM in fungi.

As SMBGCs can be highly dynamic, it is challenging to trace their evolution even across closely related species. Many studies have observed variable SMBGCs across species, but have been unable to trace the evolutionary processes generating this diversity due to the rapid evolution of SMBGCs even between very closely related species. Analyzing SMBGCs across closely related strains of the same species offers the opportunity to capture a snapshot of evolution and to better elucidate the genome scale evolutionary processes shaping the birth and death of SMBGCs. Previous studies characterizing intraspecific diversity of SMBGCs in fungi have focused primarily on the evolution of individual gene clusters [20, 23, 30–32], and only a few studies have characterized intraspecific variation in SMBGCs at the genome scale [10, 33]. None have systematically analyzed the evolution of SMBGCs within the context of whole genome evolution at the chromosome scale.

**Fig. 1:**
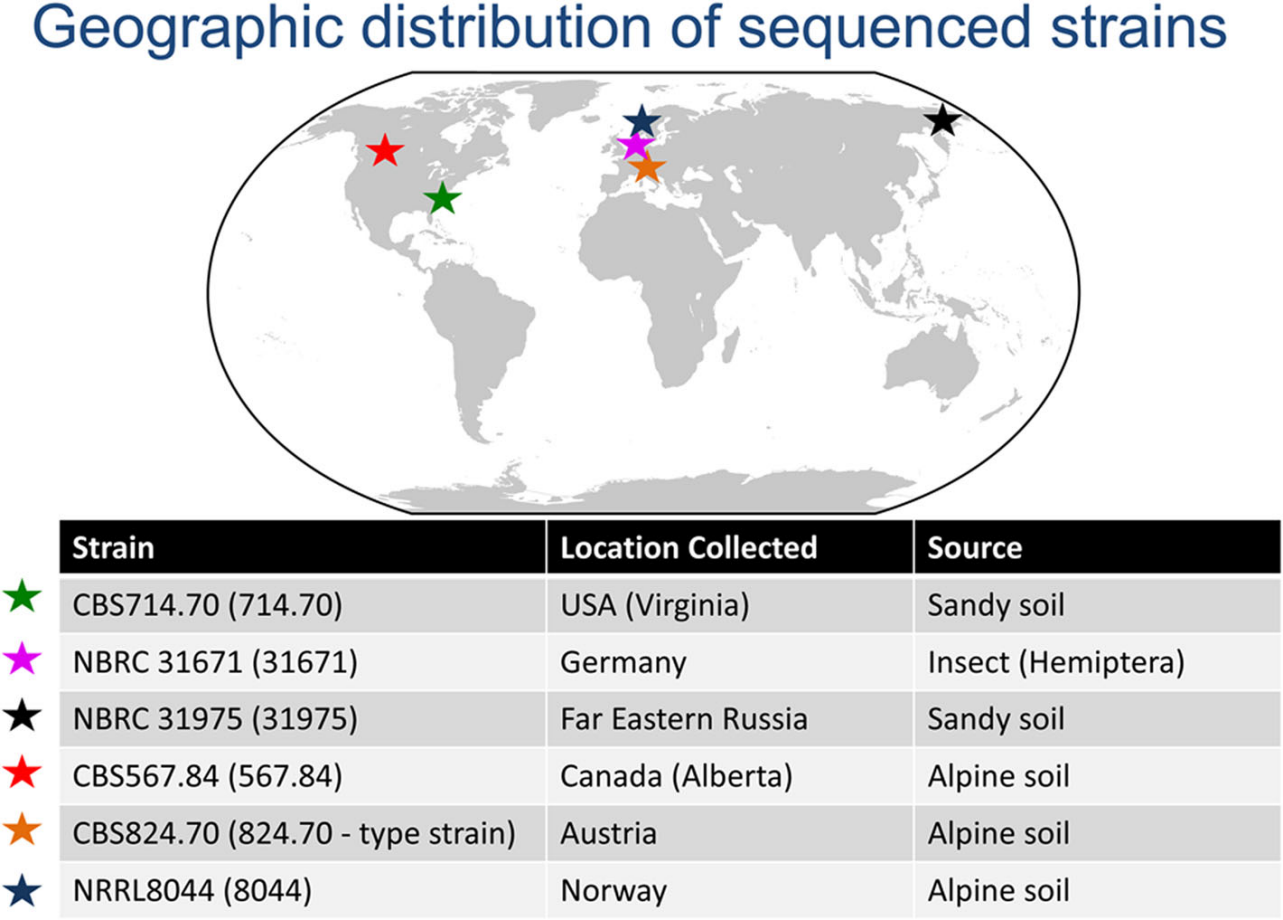
Geographic distribution of sequenced strains of *T. inflatum*. Six strains of *T. inflatum* were chosen from diverse geographic locations including Europe (Norway, Austria, and Germany), North America (Virginia, U.S.A. and Alberta, Canada), and one from Far eastern Russia (Siberia). While most strains were isolated from soil, one (31671) was isolated from a hemipteran insect species. Image of the globe is adapted from https://en.wikipedia.org/wiki/Template:Earth_Labelled_Map#/media/File:WorldMap.svg

The ascomycete fungus *Tolypocladium inflatum* is an insect pathogen and ubiquitous soil saprotroph, best known as the producer of the lifesaving immunosuppressant drug cyclosporin, which revolutionized modern medicine by making organ transplantation possible [34, 35]. The genome of the isolate from which cyclosporin was originally isolated, *T. inflatum* NRRL8044, revealed that in addition to the cluster producing cyclosporin, this strain harbors at least 36 additional SMBGCs, whose products remain chemically uncharacterized [36]. In order to investigate the impact of genome scale evolutionary processes such as chromosome rearrangements on the conservation and diversification of SMBGCs in fungi and to evaluate how well a single reference genome captures the genetic capacity for SM production, we resequenced the NRRL8044 isolate along with 5 additional geographically diverse strains of *T. inflatum* (Fig. 1) using long read PacBio technology to generate chromosome scale assemblies and a Hi-C chromosome capture technique to characterize chromosome structural variants. We demonstrate the potential birth of a novel PKS cluster through a series of translocation events involving several different chromosomes, provide phylogenetic evidence that recombination between heterologous chromosome ends likely contributes to the rapid evolution of peptaibiotic NRPS clusters located within subtelomeres, and characterize a polymorphic AF-like cluster that shows evidence of evolution by modular subunits. By examining closely related strains within the same species within the context of whole chromosomes, we demonstrate the role of genome architecture and chromosome rearrangements in shaping the evolution of secondary metabolism in fungi.

## Results

### Genome assembly and features

Assembly of PacBio reads produced high quality chromosome scale assemblies for all isolates. A previous karyotype of the NRRL8044 strain of *T. inflatum* suggested that *T. inflatum* has 6 chromosomes ranging in size from 3.8 to 6.6 Mb for a total genome size of approximately 30.45 Mb [37]. Our assemblies corresponded well to this estimated genome size and chromosomal architecture (Table 1). Strain CBS714.70 assembled de novo into only 7 contigs, including six chromosomes and the mitochondrial genome (Additional file 1: Table S1). The remaining six contigs appear to be complete chromosomes as telomeric repeats of TTAGGG [38] were identified on both ends of all but one contig (Additional file 1: Table S1). Thus, we utilized the complete CBS714.70 assembly, rather than the previously sequenced NRRL8044 strain, as a reference for further analyses.

**Table 1:** Genome Assembly Statistics

Strain	Contigs	Assembly Size (Mb)	%GC	N50 (Mb)	L75	Gene calls	Busco	Error Rate	PacBio Coverage	Illumina Coverage
714.70	7	29.86	57.74	5.07	5	8766	99.6	9.06e-03	88.5	114.67
8044	35	31.40	57.86	4.46	5	9232	99.6	8.64e-03	93.86	108.06
824.70	25	31.71	57.73	5.25	5	9221	99.6	5.12e-03	115.45	140.62
567.84	22	31.83	57.72	5.28	5	9314	99.6	5.91e-03	92.35	125.07
31671	20	30.18	57.98	4.98	5	8891	99.6	1.93e-03	88.56	112.23
31975	23	30.56	57.86	5.03	5	9200	99.6	3.82e-03	79.46	103.27

Assemblies of other strains ranged from 7 to 35 contigs. However, each strain contained 6–7 large contigs greater than 1 Mb that aligned to the assembled chromosomes of the CBS714.70 strain (Additional file 2: Figure S1, Additional file 1: Table S1). For all strains, > 75% of the total genome size (L75) was contained in the five largest contigs (Table 1). Telomeric repeats were also identified at both ends of a majority of the large contigs in strains NBRC31671, CBS567.84, and CBS824.70, and in over half of the larger contigs of NBRC31975 and NRRL8044 (Additional file 1: Table S1). Smaller contigs ranged in size from several Kb to 0.6 Mb and several contained telomeric repeats (Additional file 1: Table S1). Importantly, no SBMGs were identified on any contigs smaller than 1 Mb. Assembly using Hi-C chromosome capture data and 20-kb bins of the PacBio assembly of strain NRRL8044 did not significantly improve the assembly in terms of placement of these smaller contigs, but did validate the accuracy of the NRRL8044 PacBio assembly and supported the grouping of two contigs to form a single chromosome (Additional file 3: Figure S2). For consistency in comparisons across strains, we utilized the PacBio assemblies for identification of SMBGCs and comparative genomic analyses and the Hi-C data to examine structural variants in strain NRRL8044. For several strains (NRRL8044, NBRC31671, and NBRC31975), the chromosome corresponding to chromosome 6 of CBS714.70 was also split into two contigs. In NRRL8044, the Hi-C data showed strong interactions between these two contigs (unitig 34 and unitig 43), supporting both their grouping as a single chromosome and their orientation relative to CBS714.70 chromosome 6 (Additional file 3: Figure S2). In all other strains, these contigs also aligned to the two arms of chromosome 6 with a high degree of synteny. The sequencing error rate, estimated by mapping Illumina reads to the final pilon corrected assemblies using Bowtie2 [39] was also low (< 1 × 10^− 2^) (Table 1). Gene annotations performed in MAKER 2.28 [40] were estimated to be greater than 95% complete in all genomes by BUSCO [41].

#### Relationships between strains

**Fig. 2:**
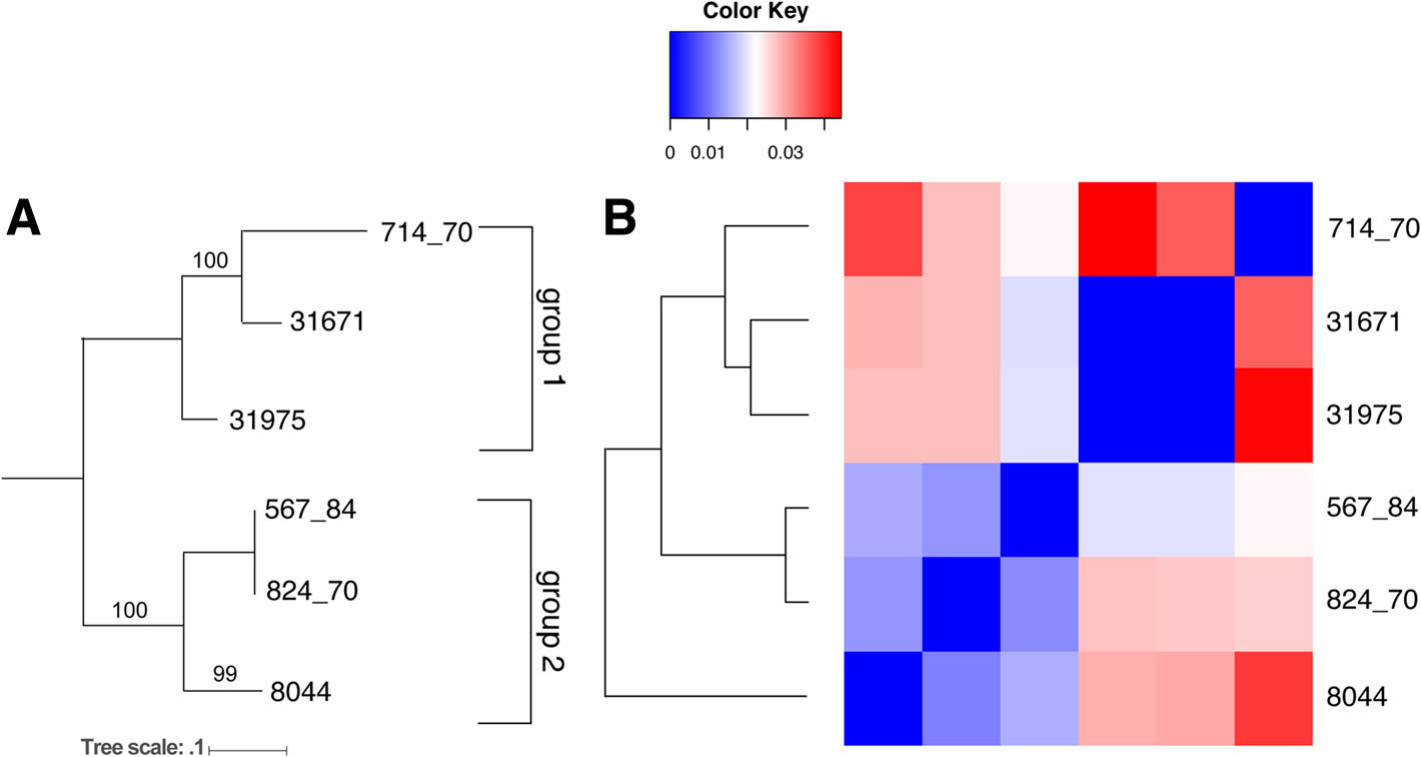
Relationships between strains of *T. inflatum.* **a** A SNP phylogeny used to assess relationships between strains showed two major groups supported by strong bootstrap support. Group 1 was comprised of 714.70, 31671, and 31975, while group 2 was comprised of 567.84, 824.70, and 8044. **b** A cladogram and heatmap showing relationships based on pairwise inversion distances between strains was also created and mostly supported the two groupings observed from SNP data, with the exception that 8044 came out as sister to all other clades rather than as part of group2. Strain 8044 was most diverged in terms of genome rearrangements from all other strains

A phylogeny based on SNPs was constructed using maximum likelihood implemented in RAxML [42]. When rooted along the longest branch, the SNP phylogeny formed two major groups. Group 1 consisted of CBS714.70, NBRC31671, and NBRC31975 and Group 2 included the type strain of *T. inflatum*, CBS824.70, as well as CBS567.84 and the previously sequenced isolate NRRL8044 (Fig. 2a). Strains did not group together by geographic origin, suggesting that *T. inflatum* either has a cosmopolitan distribution with weak biogeographic structure or may group instead by factors such as host association or environment.

#### Inversions and structural variants

Inversions and other structural variants were identified using both Assemblytics [43] to characterize small-scale structural variants < 10 kb and a custom pipeline utilizing the NUCmer aligner in MUMmer to identify both medium sized inversions (10-40 kb) and larger sized inversions (> 40 kb) between each strain compared to the reference strain CBS714.70. Each chromosome was also aligned separately using Mauve to visualize structural variants, repeats, and levels of synteny across the chromosomes and in regions surrounding SMBGCs (Additional file 2: Figure S1). Using the NUCmer pipeline, several large-scale inversions > 40 kb were identified but these inversions were not shared across all strains (Additional file 4: Table S2). A heatmap of the pairwise inversion distance between strains closely followed the phylogeny based on SNPs, showing that strains CBS714.70 and NRRL8044 were separated by the most inversions, while CBS824.70 and CBS567.84 were most similar (Fig. 2b). However, strain NRRL8044 grouped as sister to all other strains rather than clustering in group 2 with CBS824.70 and CBS567.84 as in the SNP phylogeny (Fig. 2b). Smaller scale structural variants, identified using Assemblytics, showed that insertion/deletions from 50 to 500 bp were the most common (100–165 per genome) while numbers of larger insertions and deletions of 500–10,000 bp in size ranged from 26 to 55 per genome (Additional file 5: Table S3). Repeat expansions and contractions of both smaller (50-500 bp) and larger (500–10,000 bp) size classes were also quite common ranging from 64 to 176 per genome, while tandem expansions and contractions were rare (Additional file 5: Table S3). Results showed that when compared to CBS714.70, most strains shared a similar number of small scale structural variants with the exception of NBRC31671, which contained < 60% the number of variants found in other strains (Additional file 5: Table S3).

#### Large scale chromosomal translocations

**Fig. 3:**
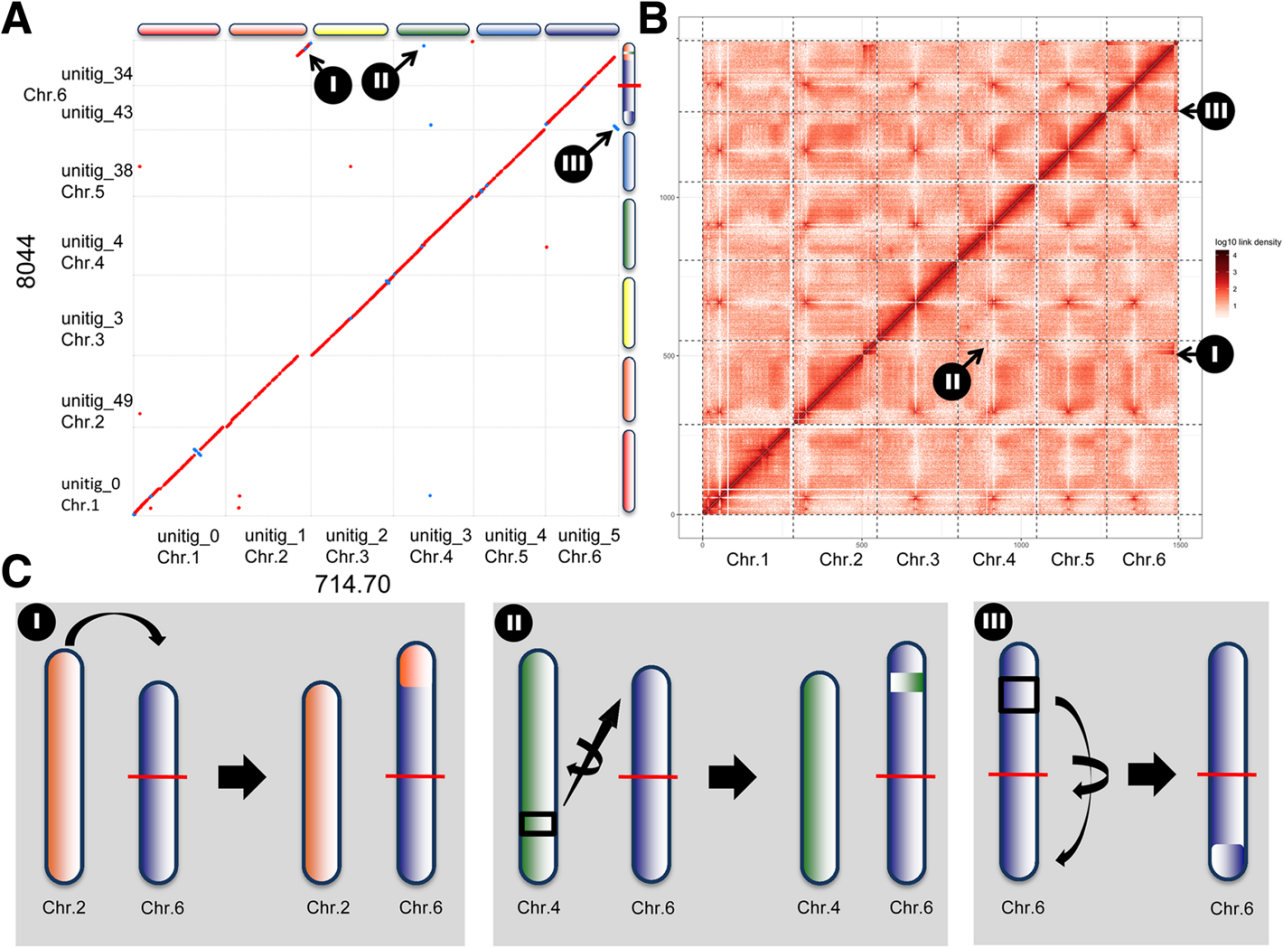
Chromosomal translocations in strain 8044. **a** MUMmer plot in which 714.70 was used as the reference genome (x-axis) and 8044 as the query (y-axis). Red dots aligning with the diagonal axis indicate syntenic aligned sequence, while blue dots represent an inversion. Dots off the diagonal indicate a translocation or exchange of DNA between chromosomes. Homologous chromosomes in each strain are shown along each axis and color coded (Chr.1 = red, Chr. 2 = orange, Chr.3 = yellow, Chr.4 = green, Chr.5 = light blue, Chr. 6 = dark blue). Genome rearrangements observed in 8044 relative to 714.70 include I) A translocation of a ~ 800 Kb segment of DNA from one end of chromosome 2 onto the end of chromosome 6 (orange segment); II) Both translocation and inversion of a small segment of DNA containing a PKS and several accessory genes from chromosome 4 to the large translocated region (I) on the end of chromosome 6 (green line); III) An intrachromosomal inversion/translocation from the right arm of chromosome 6 to the opposite (left) arm of the same chromosome. The red line bisecting chromosome 6 indicates the boundary of two unitigs [34, 43] that comprise chromosome 6. **b** Plot of 8044 Hi-C data mapped to the 714.70 assembly shows hotspots that deviate from the diagonal and support these three translocation events (Additional file 7: Figure S4). **c** Cartoon of each of the three translocation events

Genomes of all strains were aligned to the reference CBS714.70 strain using MUMmer and showed a high overall level of synteny with CBS714.70 (Additional file 6: Figure S3). However, several large-scale chromosomal rearrangements were observed in strain NRRL8044 (Fig. 3, Additional file 6: Figure S3). The end of chromosome 2 in NRRL8044 was translocated to the end of the chromosome 6 (Fig. 3a-c; I, Additional file 6: Figure S3). This major translocation is roughly 800 Kb in size. The translocation is supported both by Hi-C data for NRRL8044 mapped to the CBS714.70 assembly (Fig. 3b, Additional file 7: Figure S4) and by contiguous and high-coverage mapping of full length PacBio reads across the junction of this translocation in NRRL8044 (Additional file 8: Figure S5). Several additional translocations, potentially associated with this major event, were also observed in strain NRRL8044. A smaller (~ 20 Kb) piece of DNA from chromosome 4 was translocated and inserted into the large 800 Kb translocated region on chromosome 6 (Fig. 3a-c; II). In NRRL8044, a third large-scale intra-chromosomal translocation involved movement of approximately 200 Kb from the right arm of chromosome 6 to the other arm of the same chromosome (Fig. 3a-c; III).

#### Repeat content

RepeatMasker masked from 1 to 3% of the genomes as repetitive sequence, while de-novo methods using RepeatScout and a custom NUCmer pipeline identified between 3 and 7% and 4–9% repeats, respectively (Table 2). The differences in repeat content can be partially attributed to differences in numbers of transposable elements (TEs) between strains. When analyzed by RepeatMasker, all strains harbored both RNA and DNA elements, with LINE’s and LTRs dominating retrotransposon elements and hobo-Activator and Tc1-IS630-Pogo transposons dominating DNA elements (Table 2). Variation in numbers of LINE and LTR retrotransposons and DNA elements contributed most to differences in total number of TEs across strains. Repeat content also correlated with strain relationships. Group 1 strains consistently had a lower number of repetitive DNA and TEs than group 2 strains, particularly for LINEs and DNA elements. Similarly, although the total number of LTRs did not differ significantly, the composition of specific TEs differed between groups. Group 1 strains had more DIRS1 elements while group 2 strains had more Ty1/Copia elements (Table 2). Strain NRRL8044, from which cyclosporin was originally isolated, had a much higher number of both hAT and Tc1-IS630-Pogo superfamily DNA elements than other strains (Table 2).

**Table 2:** Repeat and Tranposable Element Content

% Total Repeat Content	714.70	31671	31975	567.84	824.70	8044
RepeatMasker	1.85%	2.18%	2.22%	2.66%	2.35%	2.66%
Repeat Scout	3.54%	4.07%	4.55%	6.67%	5.34%	6.67%
NUCmer	4.61%	4.65%	5.18%	8.45%	7.34%	8.45%
Combined nonredundant	6.17%	6.24%	6.76%	10.16%	9.03%	10.16%
# Transposable Elements	714.70	31671	31975	567	824	8044
RNA Transposons						
SINE	0	0	0	0	0	0
LINE	32	48	73	140	140	110
LTR	83	73	67	94	94	92
Ty1/Copia	27	28	24	51	50	49
Gypsy	56	45	43	43	44	43
DIRS	21	38	45	10	9	16
DNA Transposons						
TIR	48	48	65	123	91	185
hAT	5	12	38	83	56	128
Tc1-IS630-Pogo	13	10	8	9	9	26
PiggyBac	11	1	0	2	2	2
Tourist/Harbinger	0	1	1	1	1	1

#### Secondary metabolite biosynthetic gene clusters

SMBGCs were identified and defined computationally using antiSMASH [44]. A total of 51 distinct clusters were identified across all six strains. The total number of SMBGCs varied across strains from 42 in NRRL8044 to 47 in NBRC31671 (Table 3, Additional file 9: Table S4), with Group 1 isolates having a larger overall number of clusters than Group 2 isolates. Similarly, variation was detected in the total number of all metabolite subclasses. Group 1 strains had a larger number of NRPS clusters, while group 2 strains had more hybrid T1PKS-NRPS clusters, or those containing a hybrid core enzyme composed of fused Type 1 PKS and an NRPS (Table 3). All strains also harbored a cluster involved in biosynthesis of an indole or indole-terpene, which contained 7 out of 13 cluster genes matching those in the cluster for terpendole E biosynthesis [45] in *Botrytis cinerea*, as well as 5–6 additional clusters classified as “Other”, all containing an acyl-adenylating enzyme (Table 3, Additional file 9: Table S4).

**Table 3:** Secondary metabolite clusters determined by antiSMASH

	Total	Nrps	T1pks-Nrps	T1pks	Terpene	Indole-Terpene	Indole	Other
714.70	46	15	6	15	3	1	0	6
31671	47	14	8	15	4	0	1	5
31975	45	14	7	14	4	0	1	5
567.84	44	12	9	14	3	1	0	5
824.70	44	12	9	14	3	1	0	5
8044	42	12	7	14	3	1	0	5

**Fig. 4:**
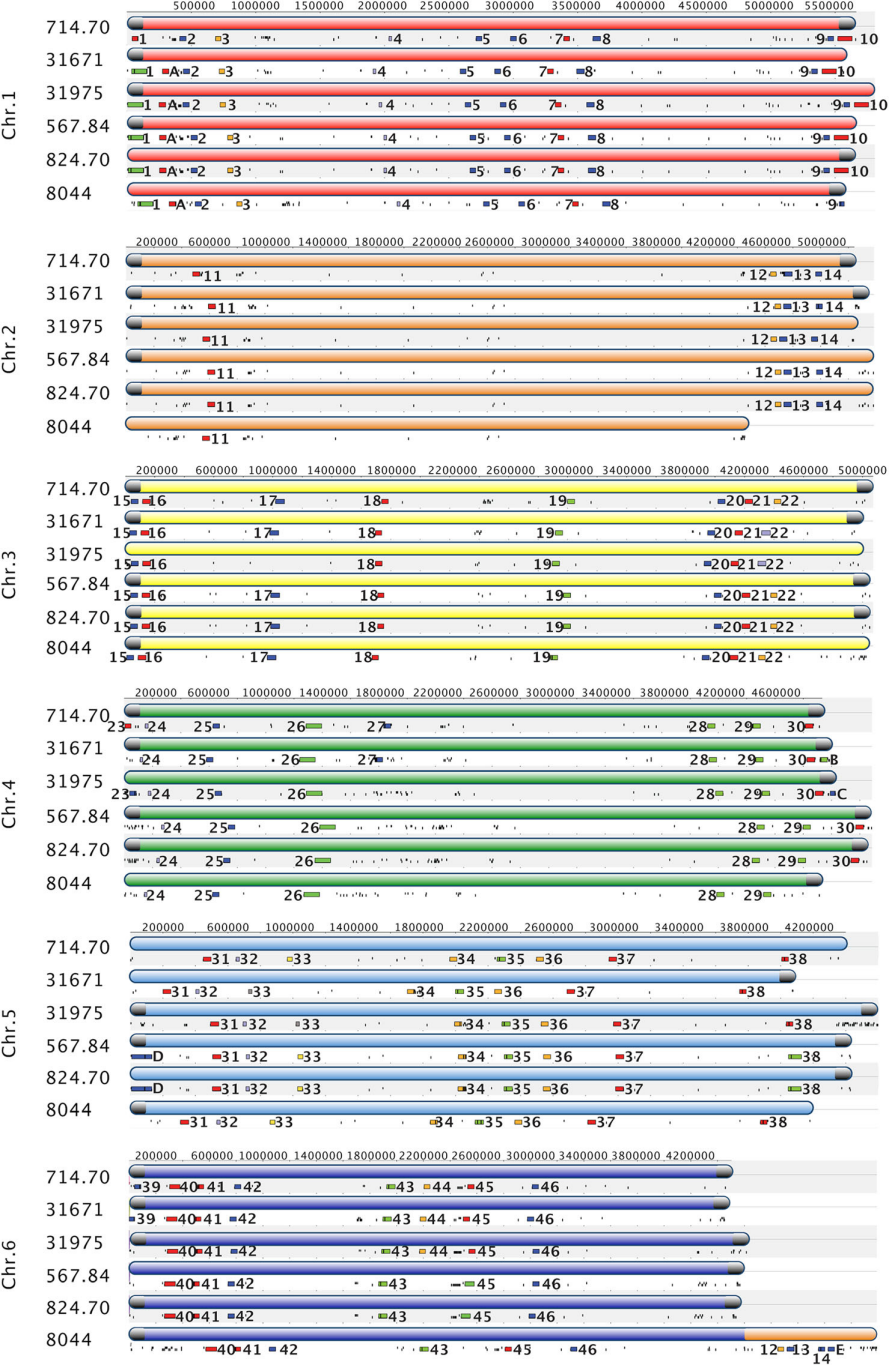
Mapping of SMBGCs onto chromosomes. Each chromosome panel contains syntenic alignments of each chromosome across all strains, with sequence coordinates at the top. Chromosomes are color coded, (Chr. 1 = red, Chr. 2 = orange, Chr. 3 = yellow, Chr. 4 = green, Chr. 5 = light blue, Chr. 6 = dark blue). Presence of telomeric repeats is indicated with a gray cap on the end of the chromosome. Below each chromosome is a track showing SMBGCs (colored blocks) and TEs (black lines). SMBGCs are numbered according to those found in the reference 714.70 genome and colored by metabolite class (red = NRPS, blue = T1PKS, green = T1PKS-NRPS, purple = terpene, yellow = Indole-terpene, and orange = other). Variable clusters found in only one or several strains but not in the reference are labelled with letters A-E

**Fig. 5:**
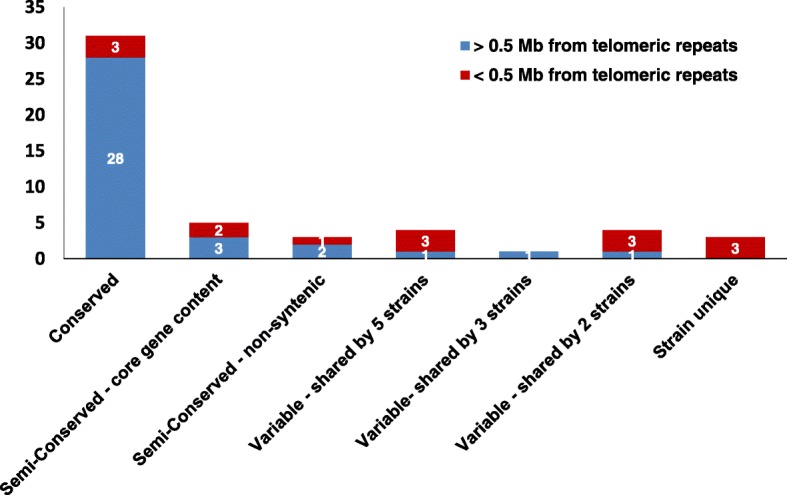
Histogram and genomic locations of conserved, semi-conserved, and variable SMBGCs. Numbers of SMBGCs located > 0.5 Mb from telomeric repeats are shown in blue, while numbers of SMBGCs located on the ends of chromosomes < 0.5 Mb from telomeric repeats are shown in maroon. Gene clusters were classified as conserved if they were present in the same syntenic location in all strains and contained the same core gene content. Clusters were classified as semi-conserved if they were present in all strains but varied in either 1) SM core genes (core gene content) or 2) genomic location (non-syntenic). Clusters were classified as variable if they were only shared by a few strains (shared by 5, 3, and 2 strains) or present in only one strain (strain unique). Semi-variable and variable clusters were enriched in regions located < 0.05 Mb from telomeric repeats on the ends of chromosomes

When SMBGCs were mapped to chromosomal location, they were distributed fairly evenly across chromosomes, without a strong bias towards a subtelomeric location (Fig. 4). Phylogenetic analyses of the A domains of NRPSs, the ketosynthase (KS) domains of PKSs, and the TS domains of TSs was performed using maximum likelihood to identify homologous core genes (Additional file 10: Figures S6, Additional file 11: Figure S7, and Additional file 12: Figure S8). SMBGCs were classified as conserved, semi-conserved, or variable based on both phylogenetic relationships and synteny. Clusters were classified as conserved if 1) they were present in all strains, 2) the core gene content (e.g.*,* NRPS, PKS, TS, and DMAT) was conserved and the core genes clustered together with strong bootstrap support in phylogenetic analysis (Additional file 10: Figures S6, Additional file 11: Figure S7, and Additional file 12: Figure S8), and 3) they occupied the same syntenic genomic location across all strains. Of a total of 51 clusters, 31 were conserved in both synteny and core gene content (Fig. 5). Conserved clusters included the cyclosporin cluster, a T1PKS cluster with 7 out of 15 genes in the cluster shared with the fumonisin SMBGC of *Fusarium* spp. [46], and a T1PKS cluster sharing 3 out of 15 genes with the stipitatic acid cluster (Fig. 4; clusters 26, 19, and 25, respectively, Additional file 9: Table S4) of *Aspergillus* spp., among others.

Clusters were classified as semi-conserved if they were present in all strains, but varied in either core gene content or syntenic location. Out of a total of 8 semi-conserved clusters, 5 occupied the same syntenic locus and retained at least one homologous core gene, but had either gained or lost a second core gene, thus changing the type of SMBGC (e.g.*,* NRPS, PKS, NRPS-PKS hybrid, TS cluster) (Fig. 4; clusters 1, 22, 33, 38, and 45, Fig. 5). Three semi-conserved clusters were found in all strains and shared the same homologous core gene(s), but were situated in different genomic locations, recently termed location polymorphisms or “jumping clusters” [10] (Fig. 4; clusters 12, 13, 14, Fig. 5). These three gene clusters were part of the large 800 Kb translocation that moved them from the end of chromosome 2 to the end of chromosome 6 in NRRL8044 (Fig. 3a-c; I, Fig. 4; orange segment of NRRL 8044 chromosome 6).

Variable clusters were either not conserved across all strains and/or contained nonhomologous core genes occupying the same syntenic locus, recently termed “idiomorphic” clusters [10]. Clusters that were not present in the reference CBS714.70 were assigned letter identifiers (Fig. 4; clusters A-E). Of the 12 variable clusters that were not shared across all strains, 4 were shared by 5 strains (Fig. 4; clusters A, 10, 17, 30, Fig. 5), 1 was shared by 3 strains (Fig. 4; cluster 44, Fig. 5), 4 were found in only 2 strains (Fig. 4; clusters 23, 27, D, 39, Fig. 5), and 3 were strain unique or found in only 1 strain (Fig. 4; clusters B, C, and E, Fig. 5). Two variable clusters were also “idiomorphic” in that they contained different core gene content at the same syntenic locus (Fig. 4; clusters 23 and D, Fig. 5).

### Genomic architecture of SMBGCs

Although SMBGCs were distributed randomly across chromosomes, a Fishers exact test comparing the genomic location of clusters classified as either less than or greater than 0.5 Mb from telomeric repeats across categories of conserved clusters versus those that varied across strains (e.g.*,* both semi-conserved and variable) was highly significant (*p* < 0.0003), indicating that variable SBMCs showed a strong bias for location towards the ends of chromosomes (Fig. 5). The majority of variable clusters (9 out of 12) were located within 0.5 Mb of telomeric repeats (Fig. 5). Although two of these (Fig. 4, clusters 23 and E) were located on the end of a contig that did not have telomeric repeats, they were also classified in this group as they were located outside of other clusters (Fig. 4; clusters 24 and 14) found to be within 0.5 Mb of telomeric repeats in other strains. Similarly, 3 out of 8 semi-variable clusters were located within 0.5 Mb of telomeric repeats (Fig. 5). In contrast, the majority of conserved clusters [28] were located internally on chromosomes and only 3 out of 31 were located near the ends of chromosomes within 0.5 Mb of telomeric repeats (Fig. 5).

All clusters unique to a single strain were located in subtelomeric regions, operationally defined here as within 100 Kb from the telomeric repeats, although subtelomeric regions are known to vary from a few kilobases to more than 200 Kb [47]. For example, strain unique clusters were found on both the left arm of chromosome 4 in strains CBS714.70 and NBRC31671 (Fig. 4; cluster 23) and the right arm of chromosome 4 in strains NBRC31671 and NBRC31975 (Fig. 4; clusters B and C). Similarly, two variable clusters were located within subtelomeres on both the left arm (Fig. 4; cluster 1) and the right arm of chromosome 1 (Fig. 4; cluster 10). Additional variable clusters that were shared by only a few strains were found within 0.5 Mb of telomeric repeats on the left arm of chromosome 5 in both CBS567.84 and CBS824.70 (Fig. 4; cluster D), on the left arm of chromosome 6 (Fig. 4; cluster 39) in CBS714.70 and NBRC31671, and on the left arm of chromosome 1 (Fig. 4, cluster A).

The genomic locations of known TE elements were also plotted onto chromosomes (Fig. 4, black lines) and genome correlation analysis was used to look for associations between SMBGCs and specific classes of TEs. Although we did not detect a strong genome-wide signal of association between TEs and SMBGCs (Additional file 13: Table S5), statistically significant associations between SMBGCs and TE elements were found on some chromosomes. Notably, SMBGCs on CBS714.70 chromosome 2 showed a significant association with LTR elements (Additional file 13: Table S5).

### Genome evolution shapes conservation and diversification of SMBGCs

To characterize the impacts of chromosome rearrangements and genome architecture on the evolution of SMBGCs, we examined relationships between cluster genomic locations and different types of structural variants, including translocations, inversions, and micro duplication/deletions events along genomic alignments of each of the six major chromosomes (Additional file 2: Figure S1). These analyses revealed several types of genome scale evolutionary processes leading to diversification of SMBGCs located near the ends of chromosomes: 1) translocations between chromosomes affecting either complete clusters or core genes, 2) insertion/deletion or recombination events impacting core gene content in clusters located within subtelomeres or other repeat rich regions of the genome, and 3) evolution of large idiomorphic clusters through independent evolution of modular parts. In contrast, several clusters located internally on chromosomes, including the cluster producing cyclosporin A (CsA), were conserved across all strains.

#### Translocation moves a PKS core gene between clusters on different chromosomes

**Fig. 6:**
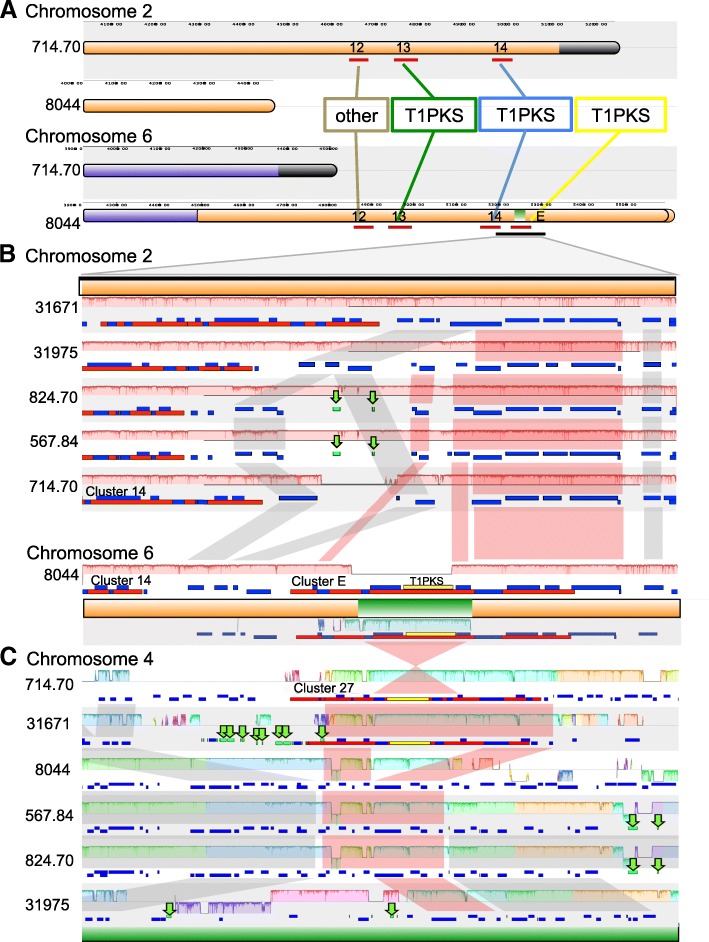
Translocation of a T1-PKS and several accessory genes between chromosomes. **a** Three PKS clusters (12, 13, and 14; red bars below chromosome) were moved as part of the large 800 Kb translocation of the end of chromosome 2 (orange) to the end of chromosome 6 (dark-blue) in 8044 (Fig. 3; I). Within this translocated region in 8044, however, an additional cluster (cluster E) was identified that was not found in any other strains and contained a T1-PKS gene that shared homology with a T1-PKS in cluster 27 on chromosome 4 and appears to have been translocated from chromosome 4 to chromosome 6 (Fig. 3; II). Between panels B and C is a cartoon of the end of chromosome 6 in 8044, showing segments translocated from chromosome 2 (orange) and chromosome 4 (green). Above this cartoon is a zoomed in genomic alignment of the region surrounding cluster E (red bar containing genes as yellow (T1-PKS) or blue (accessory gene) with **b** syntenic regions from chromosome 2 in all other strains (above, orange) showing that the T1-PKS and accessory genes are not found on chromosome 2 in other strains. Below the cartoon is a genomic alignment of the translocated region surrounding cluster E on chromosome 6 in 8044 with (**c**) the region surrounding cluster 27 on chromosome 4 (below, green) in all other strains. Only the green segment containing the T1-PKS and accessory genes aligns with the PKS and homologous accessory genes in cluster 27 on chromosome 4, but in an inverted orientation. Each genomic alignment shows the chromosome as a line, genes as blue boxes in a track below the line, SMBGCs as a red box in the gene track, and TEs as green blocks and arrows. Colored shaded bars directly above (forward) or below (inverted) the line indicate syntenic blocks. Red shading connecting the gene tracks indicates genes shared within cluster E, while grey shading between gene tracks indicates shared genes flanking the cluster

Analyses using antiSMASH revealed a SMBGC in the large 800 Kb translocation from chromosome 2 to chromosome 6 in strain NRRL8044 that was not present in the corresponding region on chromosome 2 of other *T. inflatum* strains (Fig. 6a; cluster E). Alignment of cluster E on chromosome 6 in NRRL8044 to the corresponding syntenic region on chromosome 2 in other strains showed that although genes on both the 3′ and the 5′ ends of cluster E were present across all strains (Fig. 6b; red shading), NRRL8044 contained a unique region in the middle of the cluster consisting of a PKS gene and three additional accessory genes that showed homology to a portion of cluster 27 on chromosome 4 (Fig. 6b; green segment). Genes flanking the 3′ end of the cluster were also highly conserved and syntenic (Fig. 6b; gray shading), while the region flanking the 5′ end varied in size between strains and contained unique regions of DNA in another strain (CBS714.70) as well as several Gypsy LTRs in the 5′ region in strains CBS567.84 and CBS824.70 (Fig. 6b; green arrows).

Phylogenetic analyses of the KS domain showed that the core PKS gene in cluster E (Fig. 6b; yellow) grouped with strong bootstrap support with the KS domain of a PKS gene found in cluster 27 on chromosome 4 (Additional file 11: Figure S7). When the region surrounding the PKS gene in cluster E in NRRL8044 was aligned to cluster 27 on chromosome 4, only the PKS and three adjacent accessory genes found in cluster E aligned to homologous genes in cluster 27, but in an inverted orientation (Fig. 6c). The PKS and three surrounding genes were only present in two strains, CBS714.70 and NBRC31671, located within cluster 27 on chromosome 4 (Fig. 6c; red bar). Together, these data support that in NRRL8044, the PKS and three accessory genes moved from an original location within cluster 27 on chromosome 4 to cluster E on the end of chromosome 6. Although genes within the 5′ and 3′ ends of cluster 27 were mostly conserved across all strains (Fig. 6c; red shading), all other strains lacked the region containing the PKS and accessory genes in the middle of the cluster. The genomic regions flanking Cluster 27 showed multiple independent insertion, deletion, and inversion events (Fig. 6c). Both the 5′ and 3′ flanking regions were rich in TEs (Fig. 6c; green arrows), especially LTRs (Class Gypsy), suggesting a possible role for TE mediated movement of this piece of DNA.

The movement of the PKS and three accessory genes from the middle of chromosome 4 to the end of chromosome 6 placed them in a new genomic context. AntiSMASH predicted two distinct SMBGCs surrounding the PKS and three accessory genes in each location. Each of these clusters contained different accessory genes with possible roles in secondary metabolism (Additional file 14: Figure S9). For example, in addition to the translocated genes, Cluster E on chromosome 6 contained a myb family transcription factor and an acetyl-CoA carboxylase, which is responsible for synthesis of the precursor compound malonyl Co-A, which is the substrate used by the PKS in this cluster. Similarly, cluster 27 contained an ABC transporter in addition to the MFS transporter found in both clusters (Additional file 14: Figure S9).

### Duplication, deletion, and recombination in subtelomeric SMBGCs

**Fig. 7:**
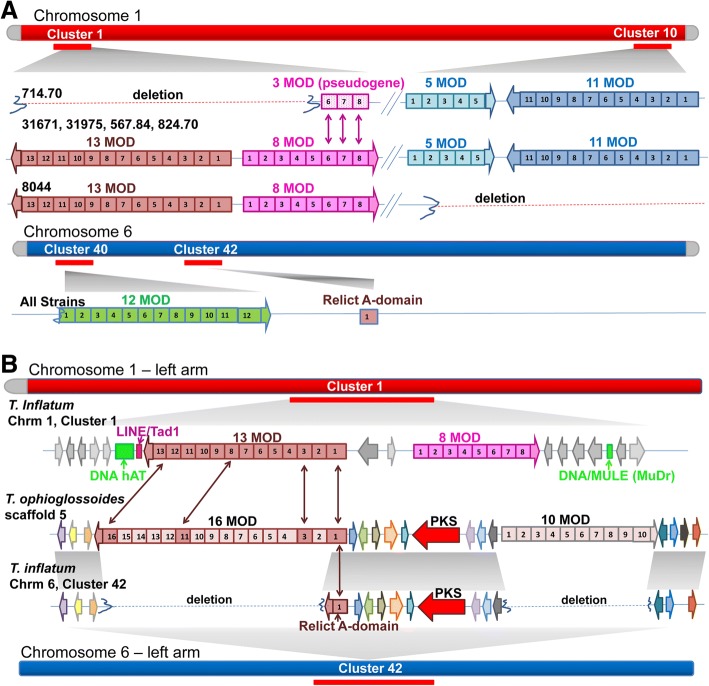
Evolution of peptaibiotic clusters in *Tolypocladium* species. **a** Clusters 1 and 10 on opposite ends of chromosome 1 and Clusters 40 and 42 on the left arm of chromosome 6 are indicated by red bars below the chromosomes and contain a total of five peptaibiotic NRPSs and one relict NRPS adenylation (A) domain. Clusters1 and 10 showed differential deletion in different strains. The entire 13 modular NRPS (containing 13 modules of A, T, and C domains numbered 1–13) and the first five modules of the 8 modular NRPS deleted in strain 714.70, leaving only a pseudogene comprised of 3 modules. Similarly, the complete cluster 10 has been deleted in strain 8044. **b** A relict NRPS A domain in *T. inflatum*, found in Cluster 42 on chromosome 6, is located within a region of DNA that aligns with a peptaibiotic cluster in the closely related sister species *Tolypocladium ophioglossoides* [24]*.* The cluster in *T. ophioglossoides*, however, contains a 16 modular and 10 modular NRPS as well as a PKS (red) [24]. Although the PKS gene was conserved, both large NRPS genes were missing in cluster 42 in *T. inflatum*. Several A domains from the *T. ophioglossoides* 16 modular NRPS grouped phylogenetically with those in the *T. inflatum* 13 modular NRPS (1 ➔ 1, 3 ➔3, 11➔8, and 16 ➔13;maroon arrows), suggesting these two large NRPS genes are homologs and that the 13 modular NRPSs moved from cluster 42 on chromosome 6 to cluster 1 on chromosome 1 in *T. inflatum*. A DNA (hAT) TE (green) and a LINE/Tad1 retrotransposon (pink) are found at the edge of the deleted 13 modular NRPS within cluster 1 in strain 8044 and one DNA Mule (MuDr) element (green) is present on the 3′ end of the cluster in all *T. inflatum* strains

Many of the variable clusters located within subtelomeric regions showed evidence of gain or loss of core genes and/or complete clusters. The two variable clusters located on each end of chromosome 1 (Fig. 4; clusters 1 and 10), as well as cluster 40 on chromosome 6, contained large multi-modular NRPSs that grouped phylogenetically with the NRPS genes *Tex1* and *Tex2,* known to produce peptaibiotics in the mycoparasitic fungus *Trichoderma virens* (Fig. 7a, Additional file 15: Figure S10). Peptaibiotics are among the largest and most rapidly evolving classes of NRPS metabolites [48, 49] and function to form pores in lipid bilayer membranes, often exhibiting antimicrobial and antiviral activity [50, 51]. They are often divergent even among closely related species [48, 49], and here we found considerable variation in the modular structure of these NPRS genes and their respective clusters even within the single species *T. inflatum*. In all strains except CBS714.70, Cluster 1, located on the left arm of chromosome 1, contained two large NRPSs comprised of 13 and 8 modules, while the 13 modular gene and all but three modules of the 8 modular gene were deleted in CBS714.70. Cluster 10, located on the opposite end of chromosome 1, contained two NRPSs of 11 and 5 modules each, but the entire cluster was missing in NRRL8044 (Fig. 4, Fig. 7a). Telomeric repeats were identified on both arms of chromosome 1 in CBS714.70 and on the right arm of chromosome 1 in NRRL8044, suggesting these represent true losses rather than a failure to assemble these regions of the genome (Fig. 4, Additional file 1: Table S1). Blastn searches of the peptaibiotic NRPSs and DNA sequences from the cluster against the entire assembly also failed to recover any matching sequences.

Alignment of the region surrounding cluster 1 across all strains showed that loss of the large NRPS core genes in CBS714.70 resulted from deletion of a ~ 88 Kb piece of DNA from the middle of the cluster (Fig. 7b, Additional file 16: Figure S11). The 3′ end of the cluster was conserved across strains and harbored all of the accessory genes found in other strains as well as a remnant pseudogene containing three NRPS A domains that grouped phylogenetically with the last three modules of the 8 modular NRPS in cluster 1 (Fig. 7a; arrows, Additional file 16: Figure S11). Alignment of the 5′ end of the cluster, however, showed that although one gene on the 5′ edge of the cluster was conserved across strains, the large 13 modular NRPS, as well as the first 5 modules of the 8 modular NRPS, were deleted in CBS714.70 (Fig. 7a, Additional file 16: Figure S11). Several DNA TEs, including a DNA/MULE (MuDr) element and the DNA hAT element *Restless*, first identified in *T. inflatum* [52], were found in all strains within the 5′ end of the cluster near the border of the region that was deleted in CBS714.70. A LINE element was also found in the 5′ end of the cluster in strain NRRL8044 (Fig. 7b, Additional file 16: Figure S11). Similarly, cluster 10 on the opposite end of chromosome 1 was completely missing from strain NRRL8044. Interestingly, cluster 9, directly adjacent to cluster 10, was truncated and also contained a DNA hAT transposon located inside the cluster (Additional file 16: Figure S11).

A third peptaibiotic cluster containing a 12 modular NRPS was found within 0.5 Mb of telomeric repeats on the left arm of chromosome 6 (Fig. 7a, cluster 40). A previous study of peptaibiotics in *T. ophioglossoides*, a sister species to *T. inflatum*, identified a genomic region that shared both synteny and gene content with a large NRPS-PKS hybrid cluster in *T. ophioglossoides* (Fig. 7b) [24]. Homologs of the core PKS gene and nearly all additional accessory genes present in the *T. ophioglossoides* cluster were found in syntenic arrangement in *T. inflatum,* but the two large 16 modular and a 10 modular NRPSs present in the *T. ophioglossoides* cluster were missing in all strains of *T. inflatum*, except for a single remaining “relict” NRPS A domain [24] (Fig. 7b, Additional file 16: Figure S11). In our assemblies, this relict A domain, as well as the PKS gene and other accessory genes shared with the *T. ophioglossoides* cluster, were located in cluster 42 on chromosome 6 (Fig. 7b). Phylogenetic analyses suggested that this relict A domain was most closely related to the first module of the 16 modular NRPS in *T. ophioglossoides* and the first module of the 13 modular NRPS found in *T. inflatum* [24]. Our phylogenetic analyses supported this previous finding (Fig. 7b; arrows, Additional file 16: Figure S11), and showed that although there is not a one-to-one correspondence between A domains from different modules of the 16 modular NRPS in *T. ophioglossoides* and the 13 modular gene in *T. inflatum*, several other A domains from these two genes also grouped together phylogenetically (Fig. 7b; arrows; Additional file 15: Figure S10), supporting that these two NRPSs are likely homologs [24]. The phylogenetic grouping of the relict A domain in cluster 42 on chromosome 6 with A domains from the 13 modular NRPS in cluster 1 on chromosome 1 in *T. inflatum* (Additional file 15: Figure S10) suggests the possibility that this large 13 modular NRPS has moved from its ancestral location within cluster 42 on chromosome 6 to another cluster (cluster 1) on chromosome 1.

#### Modular evolution of an idiomorphic AF-like cluster

**Fig. 8:**
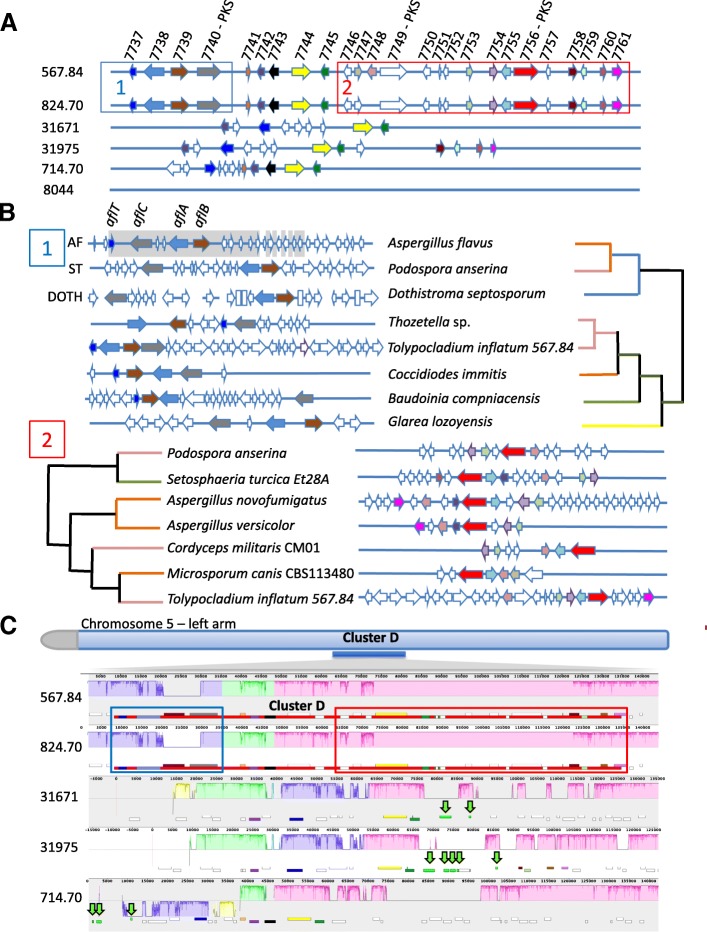
Evolution of an idiomorphic AF-like cluster in *T. inflatum*. **a** Only two strains (567.84 and 824.70) harbor a complete cluster D, which is composed of at least two subclusters with independent evolutionary histories. Subcluster 1 (Box 1; blue) contains a PKS (7740) and three additional genes (7737, 7738, 7739) with homology to *aflC*, *aflT*, *aflA*, and *aflB*, respectively, of the AF mycotoxin cluster of *Aspergillus flavus*. Subcluster 2 (Box 2; red) contains two additional PKS genes (7749 and 7756) as well as a large number of accessory genes. All strains except 8044 harbor a pair of genes (7744 and 7745) that mark a boundary between the two subclusters. No homologs of genes from cluster D were detected in 8044. **b** Each subcluster shows a distinct pattern of co-diversification with genes found in other SMBGCs mined from a database of 340 fungal taxa. For subcluster 1, the four AF homologs are consistently clustered together but in different arrangements in AF-like clusters found in distinct Eurotiomycete (orange), Dothideomycete (green), and Sordariomycete (pink) taxa. The LD block found in the AF cluster across various *Aspergillus* spp. is shaded grey. Subcluster 2 shares four to seven genes with distinct SMBGCs found in other Eurotiomycete (orange), Dothideomycete (green), Leotiomycete (yellow), and Sordariomycete (pink) taxa. **c** Mauve alignment of the region of the chromosome containing cluster D and the syntenic regions in other strains revealed numerous genome rearrangements within and surrounding cluster D. The chromosome of each strain is shown as a line with genes marked in dark blue in a track below the line and colored shading above (forward) or below (inverted) the line indicating syntenic blocks relative to 714.70. Subclusters 1 and 2 are shown as blue and red boxes, respectively. A number of LTR TEs (green arrows) flank the cluster

An intriguing variable cluster, located in a subtelomeric region on the end of the left arm of chromosome 5 in only two strains (CBS824.70 and CBS567.84) (Fig. 4; cluster D), contained a PKS and several other genes showing homology to those from the cluster in *Aspergillus flavus* responsible for producing the carginogenic mycotoxin and food contaminant aflatoxin (AF) [53, 54] (Fig. 8a). In *A. flavus*, the gene cluster producing AF contains 25 genes and *aflC* is the core PKS (Fig. 8b). The 5′ end of cluster D in *T. inflatum* strains CBS567.84 and CBS824.70 contained homologs of *alfC* as well as three additional genes showing high sequence similarity to *aflB*, *aflA* and *aflT* from the AF cluster (Additional file 17: Table S6). These homologs of the AF genes were not detected in any other strains through protein or DNA based BLAST searches using cluster genes, intergenic regions, or the genomic region of the entire AF cluster as queries of the complete *T. inflatum* assemblies.

Phylogenetic analyses of homologs of all genes in cluster D mined from a database of 340 other ascomycete taxa identified two distinct regions or subclusters of the complete cluster D that showed phylogenetic relationships consistent with independent co-diversification. Thus, the larger cluster D observed in CBS567.84 and CBS824.70 appears to be an “idiomorphic” cluster, composed of two or more independently evolving subclusters or “alleles” [10]. Subcluster 1, spanning the 5′ end of the cluster, contained the four AF homologs (Fig. 8a, b; subcluster 1, blue box; genes 7737–7740, Additional file 18: Figure S12) as well as additional accessory genes, and showed a distinct evolutionary history from genes located on the 3′ end of the cluster. Similarly, phylogenetic analyses of homologs of cluster genes mined from these 340 other ascomycete taxa identified a group of genes from the 3′ end of the cluster comprising subcluster 2 (Fig. 8a, b; subcluster 2, red box; genes 7746–7758, Additional file 18: Figure S12), including the two additional PKS core genes and additional accessory genes. The phylogenetic relationships of genes within subcluster 2 were consistent with independent co-diversification with SMBGCs found in Eurotiomycete, Dothideomycete, Leotiomycete, and Sordariomycete fungi [21] (Fig. 8b). Two genes located in the middle of cluster (7744 and 7745), between the boundaries of the two subclusters, were conserved across all *T. inflatum* strains and encoded enzymes not known to have roles in secondary metabolism. The gene 7745 encoded a putative long chain fatty acid CoA ligase, while 7744 encoded a putative exo-1, 3-beta-D glucanase (Additional file 17: Table S6). In contrast to most other genes in the cluster, the phylogeny of 7745 (Fig. 8a, c; yellow), in particular, more closely followed the species phylogeny (Additional file 18: Figure S12), potentially indicative of housekeeping genes.

Other AF-like clusters containing at least three homologs of these same four AF biosynthetic genes have been previously identified in Dothideomycete (dothistromin; DOTH) and Eurotiomycete (sterigmatocystin; ST) fungal classes [21] (Fig. 8b). The four AF gene homologs in cluster D were arranged in alternative orientations compared to their orientation in the AF cluster of *A. flavus* and these other AF-like clusters (Fig. 8b). *T. inflatum* homologs formed a clade with genes in other AF-like clusters from *Coccidioides immitis* (Eurotiomycetes)*, Thozetella* sp. PMI_491 (Sordariomycetes), *Glarea lozoyensis* (Leotiomycetes), and *Baudinia compniacensis* (Dothideomycetes) (Fig. 8b, Additional file 18: Figure S12). This clade was usually sister to the clade containing homologs from all previously characterized AF and AF-like (e.g.*,* ST, DOTH) clusters (Fig. 8b; subcluster 1, Additional file 18: Figure S12). Previous studies of diversity of the AF SMBGC across both *Aspergillus* species and isolates of *A. flavus* [31, 55] have shown that these same four genes occur within one of the largest linkage disequilibrium (LD) blocks (Fig. 8b; grey shaded boxes in AF cluster) found in the AF cluster.

The complete cluster D was found in only two strains, CBS824.70 and CBS567.84, although several strains (NBRC31671, NBRC31975, and CBS714.70) contained scattered genes from the 5′ end (Fig. 8a; subcluster 1; gene7737), middle (Fig. 8a; genes 7744 and 7745), and/or 3′ end (Fig. 8a; subcluster 2; genes 7758–7761) (Additional file 19: Table S7) of the cluster. Several also contained additional genes in the region syntenic with cluster D that were not present in CBS567.84 and CBS824.70 (Fig. 8a; white genes). Interestingly, none of the genes from cluster D were present in strain NRRL8044 (Fig. 8a, Additional file 19: Table S7). Strain NBRC31975 was the only strain that contained genes from both subcluster 1 and subcluster 2 as well as the two genes in the middle of the cluster (7744 and 7745)(Fig. 8a). The length of sequence between the genes flanking the two subclusters in strain NBRC31975, as well as between genes flanking subcluster 1 in other strains, was significantly shorter than the distance in CBS824.70 and CBS567.84 (Fig. 8a). Genomic alignments of the region surrounding the cluster across all *T. inflatum* strains showed multiple retrotransposons, (Fig. 8c; green arrows), primarily Gypsy LTRs, scattered in the region directly 3′ of genes in the middle (7744 and 7745) between subcluster 1 and subcluster 2. Similarly, CBS714.70 showed an inversion within subcluster 1 and contained several Gypsy LTR transposons just 5′ of the cluster (Fig. 8c; green arrows).

#### Conservation of the Cyclosporin biosynthetic cluster

**Fig. 9:**
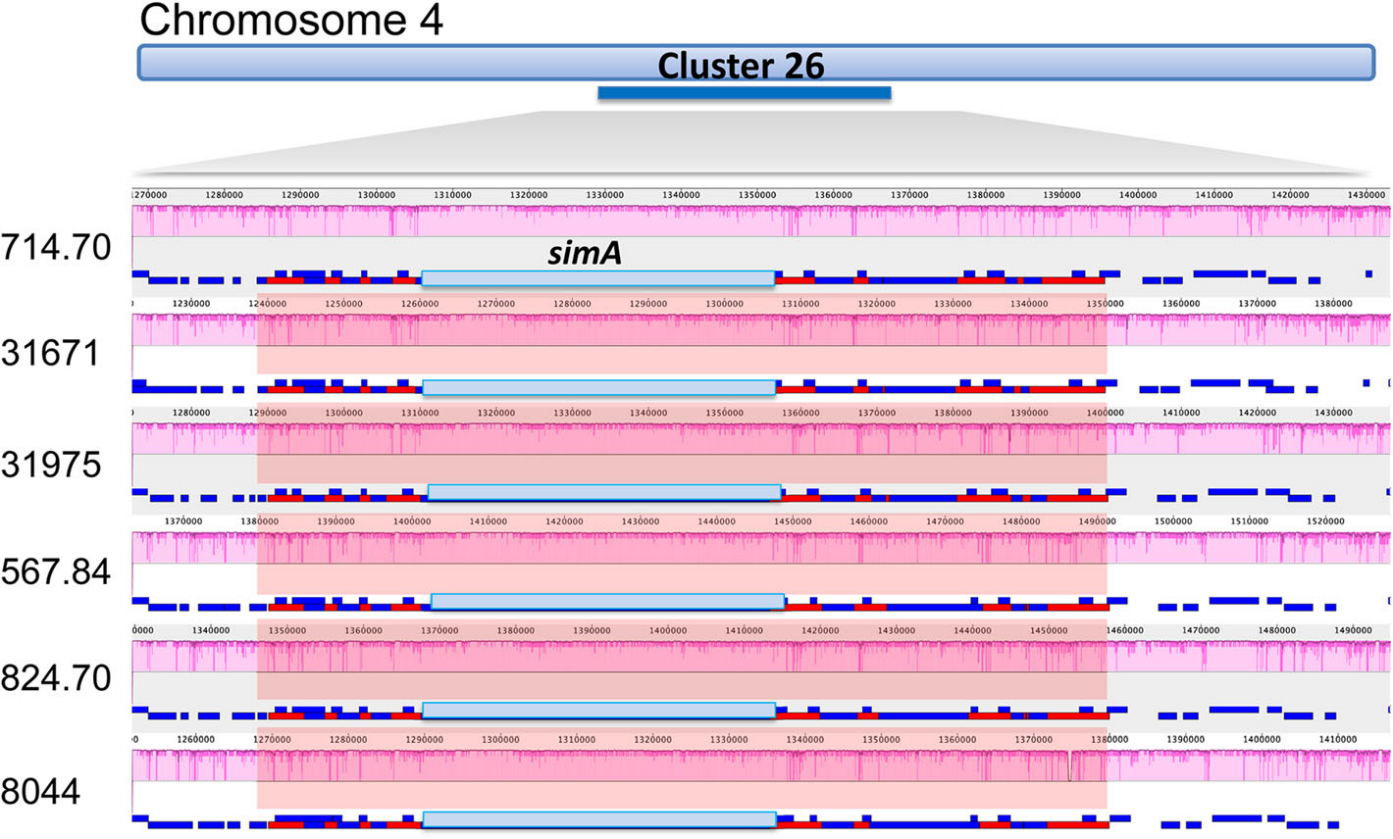
Conservation of the CsA cluster. Mauve alignment of the SMBGC responsible for CsA biosynthesis (cluster 26) in the middle of chromosome 4 across all six *T. inflatum* strains shows high synteny (red shaded regions) at the DNA level and complete conservation of gene content (blue boxes on track below each syntenic block). Cyclosporin synthase (*simA*) is shown in light blue

In contrast to these rapidly evolving clusters located on the ends of chromosomes, several clusters located more internally on chromosomes, including the cyclosporin cluster, were highly conserved. The cyclosporin cluster was found roughly 1.4 Mbp away from the telomeric repeats on the left arm of chromosome 4 (Fig. 4; cluster 26). Genomic alignments showed synteny and complete conservation of gene content of the cyclosporin gene cluster across all *T. inflatum* strains (Fig. 9). Analyses of selection of the cyclosporin synthase gene (*simA*), responsible for biosynthesis of the peptide backbone structure of cyclosporin, found no codon positions under positive selection and showed an average dN/dS ratio of 0.204 (Additional file 20: Table S8). In contrast, 84 codon positions showed statistically significant (*p* < 0.05) evidence for purifying selection (Additional file 20: Table S8). Several other clusters located internally on chromosomes, including a cluster containing homologs of the fumonisin biosynthetic cluster in *F. graminearum* (Fig. 4; cluster 19), and a cluster showing similarity to the cluster producing stipitatic acid in *Aspergillus spp*. (Fig. 4; cluster 25), were also highly conserved across all strains (Additional file 21: Figure S13).

## Discussion

Our results show that genome scale evolutionary processes such as chromosomal rearrangements and transposition contribute substantially to the evolution of intraspecific diversity in SMBGCs in *T. inflatum*. We show movement of a PKS gene from one cluster to another in strain NRRL8044, potentially forming a new SMBGC through a series of translocation events that bring together different parts of the cluster from three different chromosomes. We also demonstrate that genome architecture and genomic location of a cluster strongly impacted the evolution of SMBGCs. Although SMBGCs were more or less evenly distributed along chromosomes, clusters located towards the end of chromosomes, particularly within subtelomeric regions, were significantly more variable across strains. In other organisms, ranging from humans to yeast to the malarial parasite *Plasmodium falciparum*, subtelomeres are highly dynamic regions of the genome [56, 57], comprising hotspots for recombination between both homologous and heterologous chromosomes [58, 59], as well as for insertion and deletion events [57]. They often harbor rapidly evolving gene families involved in evading host-immune responses or in organismal adaptation to particular niches [60], including SMBGCs in fungi [61, 62].

We characterize several different mechanisms contributing to the diversification of SMBGC clusters on the ends of chromosomes. The movement of core SM genes between different chromosomes and/or clusters, mediated by either translocation, unequal crossover, or the activity of TEs, plays an important role in the diversification of SMBGCs in *T. inflatum*. We show evidence for the duplication and/or deletion of core SM biosynthetic genes or modular parts of SMBGCs located within subtelomeres. The presence of both DNA and LTR TEs surrounding and within these SMBGCs support their possible involvement in these processes. In contrast, localization internally on chromosomes or in regions of reduced recombination contributed to the conservation of SMBGCs with important roles in the lifecycle of *T. inflatum,* such as the cyclosporin cluster.

### Gain, loss, and movement of core SM genes between chromosomes

As core SM genes are essential for production of the backbone structure of SMs, their gain, loss, or movement within the genome has potential to generate dramatic changes in metabolites produced. Previous studies have observed movement of whole SMBGCs to new locations in the genome, a phenomenon termed “jumping clusters” [10]. We observe this phenomenon in the relocation of three clusters in the large translocation event from one end of chromosome 2 to one end of chromosome 6 in NRRL8044. Our data also suggest, however, that the exchange or movement of SMBGC component parts, either core SM genes (e.g., NRPSs, PKSs, TS) or modular subclusters, either between existing SMBGCs or to a new location in the genome where they may associate with different accessory genes, is a potential mechanism for generating novel SMBGCs. The movement of a core PKS gene and several adjacent genes from cluster 27 located on chromosome 4 (Fig. 6; cluster 27) in strain NRRL8044 to the large 800 Kb translocated region on the end of chromosome 6 placed these genes in a new genomic context adjacent to other genes with possible roles in SM biosynthesis or self-protection (Fig. 6; cluster E, Additional file 14: Figure S9).

Similarly, analyses of peptaibiotic clusters and phylogenetic analyses of A domains from peptaibiotic NRPSs in *T. inflatum* and its sister species *T. ophioglossoides* suggest movement of a large 13 modular core NRPS between clusters located on different chromosomes (Fig. 7b; cluster 42 and cluster 1). We also observed deletion and loss of several large peptaibiotic NRPSs within subtelomeric clusters on both ends of chromosome 1 in different *T. inflatum* strains (Fig. 7a, Additional file 16: Figure S11). Thus, the movement and deletion of large PKS and NRPS core SM biosynthetic genes from SMBGCs is not uncommon in *T. inflatum* and represents an important evolutionary mechanism driving diversification of clusters, even within a single species.

These events could be mediated either by TEs or through unequal crossover between subtelomeric repeats as has been observed for several other rapidly evolving gene families in eukaryotes [59–61, 63]. Previous studies have implicated LTR transposons located near SMBGCs in both translocation and insertion/deletion events [10] or have found SMBGCs to be significantly associated with TEs [62, 64]. In addition to LTR transposons observed in close vicinity to several of the variable clusters in *T. inflatum*, we found several DNA transposons, including a DNA-hAT [52], located inside cluster 1 near the border of the deletion event that removed the large 13 modular NRPS and five modules of the 8 modular NRPS in cluster 1. A DNA/MULE (MuDr) element and a non-LTR LINE/Tad1 retrotransposon also flanked this deletion (Fig. 7a, b, Additional file 15: Figure S10). A DNA-hAT transposon was also found in a truncated cluster (cluster 9) on the opposite end of chromosome 1 in strain NRRL8044 (Additional file 15: Figure S10). The DNA-hAT transposon *Restless* was previously found in the 5′ flanking region of a cluster involved in the production of destruxins, another class of immune modulating peptides produced by the related insect pathogenic fungus *Metarhizium* [65], leading to degeneration of the cluster and inability to produce destruxins. Our results support that both DNA and LTR TEs associated with the large NRPSs in cluster 1 in *T. inflatum* may mediate deletion and/or movement of these large NRPS core genes.

### Modular evolution of a polymorphic AF-like cluster

The large polymorphic cluster D in the subtelomeric region of the left arm of chromosome 5 is a composite cluster composed of at least two independently co-diversifying subclusters, each containing genes that occur in different SMBGCs of other ascomycete fungi (Fig. 8b). This cluster showed a modular structure similar to an “idiomorphic” cluster recently characterized in *A. fumigatus* in which modular subclusters or “alleles” were arranged in different combinations within the SMBGC and the regions between subclusters were enriched in TEs [10]*.* Subcluster 1 in cluster D contained homologs of four genes from the AF cluster, including the PKS *aflC* and several surrounding genes (*aflT*, *aflC*, and *aflB*) (Fig. 8b). The AF cluster itself also contains non-recombining modular blocks that have been shown to have different evolutionary histories and to co-diversify across five *Aspergillus* species [20]. Notably, the largest LD block described in *A. flavus* encompasses *aflA*, *aflB*, *aflC*, and *aflT* found in subcluster 1 and several additional genes on the 3′ end of these four genes [31, 55] (Fig. 8b; gray shaded boxes). Recombination junctions were found in genomic regions with a relatively high GC content and LD blocks were located between these regions [31]. AFs are carcinogenic polyketides that are produced primarily by *Aspergillus* species [66] and AF B1 is considered one of the most toxigenic substances known. It is responsible for hundreds, if not thousands, of human deaths worldwide [67, 68]. *T. inflatum* has never been shown to produce an AF type compound. However, AF was recently shown to increase fitness of AF producing strains of *A. flavus* in the presence of an insect competitor or herbivore when compared to non-producing strains [69]. It is known to be toxic to a variety of insects [70, 71], suggesting a possible rationale for the existence of an AF-like toxin in *T. inflatum*.

Other clusters containing homologs of genes in the AF cluster include the cluster responsible for biosynthesis of the toxic metabolite DOTH in the pine pathogen *Dothistroma septosporum*, and related clusters in other ascomycetes that contain at least three of the four AF homologs (*aflC*, *aflB*, and *aflA*) [21] (Fig. 8b). The set of four AF homologs are found clustered in most of these taxa and thus appear to be inherited as a module or unit (Fig. 8b). The only other large AF-like cluster found to date in the Sordariomycetes, to which *T. inflatum* belongs, is the ST cluster in *Podospora anserina*. The ST cluster has been shown to be horizontally transferred from *Aspergillus* based on multiple lines of evidence including gene phylogenies which conflict with species relationships, highly conserved synteny with the ST cluster, and conserved function [72, 73]. In our study, the gene trees of the four homologs of AF genes in *T. inflatum* consistently conflicted with the species phylogeny, suggesting potential HGT of AF genes to *T. inflatum* as well. However, the *T. inflatum* genes grouped most closely with those from another Sordariomycete, *Thozetella* sp. (Chaetosphaeriales) in a clade containing other classes of fungi including *Coccidiodes immitis* (Eurotiomycete), *Baudoinia compniacensis* (Dothideomycete), and *Glarea lozoyensis* (Leotiomycete) (Fig. 8b; Additional file 18: Figure S12). This clade formed a sister clade to the AF cluster and all other characterized AF-like clusters. Thus, the most likely candidate for a HGT donor would be *C. immitis* rather than the AF producing *Aspergillus* spp. Given the very limited distribution and complex topology in this clade and lack of synteny of other genes in the *T. inflatum* cluster with other AF-like clusters, it is not reasonable to test a specific hypothesis of HGT.

Another possible, but unparsimonious, explanation is that an AF-like cluster was present in the ancestor of Sordariomycetes, Eurotiomycetes, and Dothideomycetes and has been lost in most present day taxa of these groups. A previous study of dermatophyte fungi identified two distinct SMBGCs occupying the same syntenic location and concluded that an ancestral cluster containing all elements found in the locus underwent differential loss of parts of the cluster in different species, leading to distinct cluster types in extant species [26]. The presence of an AF-like cluster in only two strains of *T. inflatum* is intriguing and suggests several possible scenarios: 1) the cluster was gained through HGT in only these two strains, 2) the cluster was present in an ancestor of all these fungi and has undergone degeneration in the majority of strains from an SMBGC present in the ancestor, or 3) the cluster has undergone degeneration after HGT, a phenomena that has observed in the SMBGC responsible for production of depudecin in *Alternaria brassisicola* [25].

Several lines of evidence suggest that cluster degeneration in all strains except CBS567.84 and CBS824.70, either from an ancestrally inherited cluster or after a recent HGT event to *T. inflatum*, may be a more likely explanation of the discontinuous distribution of this cluster within *T. inflatum*. The cluster is located in a subtelomeric region showing evidence of multiple inversions and other rearrangements and several strains contain multiple TEs on either side of the cluster. Cluster degeneration has also been observed in the AF cluster itself, which is also located in subtelomeric regions on the right arm of chromosome 3 of *A. flavus*, leading to loss of AF production. Segmental aneuploidy or large-scale gene loss events have been reported previously in the AF cluster of *A. flavus* [53], including in strain NRRL21882 (Afla-Guard®), which is missing the entire AF gene cluster. Other studies have characterized differing patterns of gene loss in the AF cluster across worldwide populations of *A. flavus* [31]. Additionally, available evidence suggests that the DOTH cluster, which is fragmented and encoded by six smaller clusters dispersed across a single chromosome in *D. septosporum*, as well as related clusters in other Dothideomycetes, arose from a larger ancestral cluster that underwent fragmentation and subsequent differential recruitment of new genes in different taxa [21]. Together these results suggest that these same four AF cluster genes have co-diversified together as a modular unit over multiple evolutionary time scales, while compositional diversity of AF-like clusters, such as those identified in *T. inflatum*, have been generated by gain and loss of modular subclusters and accessory enzymes. A recent study of an idiomorphic locus in *A. fumigatus* showed evidence of TEs in regions between subclusters [10]. While TEs were not found between the subclusters in our study, LTR elements were present in flanking regions in all *T. inflatum* strains lacking cluster D.

### Conservation of SMBGCs

In contrast to other examples showing diversification of SMBGCs due to genome rearrangements, several clusters including the cyclosporin SMBGC, were highly conserved in both gene content and synteny across all strains (Fig. 9). Cyclosporin has immunosuppressive activity in both humans [74] and insects and thus a function in pathogenicity towards insects [75], which could serve as a selective force leading to its maintenance in this insect pathogenic fungus. Similarly, cluster 19 contained several genes homologous to those in the cluster producing fumonisins, which has high toxicity towards mammals [76, 77], but unknown activity in insects, was also highly conserved (Additional file 21: Figure S13). In a study examining conservation of SMBGCs in related insect pathogenic fungi within the genus *Metarhizium*, the cluster responsible for production of destruxins was found to be conserved across host-generalist species but degenerated or absent in some host-specialist species. It is possible that the ability to produce this broadly insecticidal compound may have been maintained in host-generalist species by selection [65]. We show that the cyclosporin synthase gene *simA* is under purifying selection within *T. inflatum*. While some have argued that the evolution of genome architecture is dominated by neutral processes rather than selection for specific gene arrangements [78], SMBGCs, composed of functionally related genes that may be beneficial for the producing organism, pose some challenges to this theory. In addition to evidence for purifying selection observed within the *simA* NRPS at the codon level, future research using a larger population sampling of *T. inflatum* isolates may address the role of selection in suppressing recombination and preventing degeneration of clusters that serve important functions for the *T. inflatum* lifestyle.

## Conclusions

Chromosome scale assemblies of six strains of the cyclosporin producing fungus *T. inflatum* enabled examination of the role of genome architecture and chromosome rearrangements in the conservation and diversification of SMBGCs within a single species. Our results demonstrate that movement of core SM genes (PKS, NRPS, TS, DMAT) involved in production of the metabolite backbone structures may occur more frequently in fungal genomes than previously recognized and contributes substantially to the evolution of novel metabolite clusters in fungi. We show the potential birth of a new PKS cluster through a series of translocation events that moved a PKS and several adjacent genes from one chromosome to another and provide phylogenetic evidence supporting movement of large peptiabiotic NRPSs between clusters located on the ends of different chromosomes. The presence of both DNA and LTR TEs adjacent to or within these rearranged clusters suggests a role for both classes of TEs in the movement of SM core genes within fungal genomes.

This research also demonstrates that genomic location strongly impacts the rate of evolution of SMBGCs in fungi. We characterize several genetic mechanisms by which location of a SMBGC within subtelomeric regions drives more rapid diversification. Phylogenetic relationships among A-domains from peptaibiotic NRPSs from *T. inflatum* suggests that like other rapidly evolving genes in animals and plants involved in pathogenesis or niche-adaptation, SM genes in fungi also diversify through exchange of DNA between the ends of heterologous chromosomes, mediated either by TEs or through unequal crossover. We also uncover an allelic or “idiomorphic” AF-like cluster present in the subtelomeric region of only two strains of *T. inflatum* that contains two subclusters showing independent patterns of co-diversification. This cluster was not detected in the previously published genome of *T. inflatum*, thus highlighting the cryptic diversity of SMBGCs in fungal populations and the importance of screening for intraspecific diversity in drug discovery programs. In contrast, conservation of several clusters (cyclosporin, fumonisin-like, and ST-like), all located internally on chromosomes, suggests the hypothesis that selection may maintain clustering and conservation of SMBGCs with adaptive functions for the producing organism. This research elucidates how genome architecture and chromosome rearrangements contribute to the divergence of SMBGCs within fungal species and provides a platform for future discovery of novel metabolites from this medically important fungus.

## Methods

### Strains and DNA isolation

Tolypocladium strains CBS714.14, CBS567.84, and CBS824.80 (type strain for *T. inflatum*) obtained from CBS and NBRC31671 and NBRC31975 obtained from NITE in Japan were grown for 2 weeks on ½ strength corn meal agar to produce conidia. 1 mL of a 1 × 106 spores/mL suspension was used to inoculate 100 mL of liquid potato dextrose broth and grown for 3–4 days shaking at 150 rpm at 25 °C. Mycelia was harvested and lyophilized before extraction with the Omniprep kit for Fungus (G-Biosciences, Cat# 786–399). An Illumina TruSeq library was prepared for each strain and sequenced to a depth of approximately 30-50x coverage on the HiSeq PE 80 and 100 bp runs. PacBio 20 kb libraries were prepared at the Mayo Genome Sequencing Center and sequenced to a depth of approximately100x coverage (4–6 SMRT cells/strain).

### Transcriptome sequencing and analyses

Conidia from strains grown two weeks on ½ strength corn meal agar and 1 × 10^6^ spores were inoculated into 100 mL of both Czapek-Dox broth and SM producing media [79]. Two replicate flasks were grown for each strain. Tissue for RNA extraction was harvested at 7 days and RNA was extracted using TRIzol (Invitrogen) according to the manufactures protocol. RNA libraries were prepared using the TruSeq RNA kit and sequenced as a depth of 12 multiplexed samples per lane on the Illumina Hi-Seq 2500 PE with 100 bp reads. RNA-Seq reads were first trimmed to 75 bp to remove poor quality reads at the beginning (15 bp) and end (10 bp) of the reads and reads with an average quality score of < 20 were removed. Transcripts were assembled using Trinity [80] and input into MAKER.

### Genome assembly and annotation

Raw PacBio reads were subfiltered and assembled in the HGAP assembler 3 [81]. Initial assemblies were then iteratively polished in QUIVER until the consensus concordance of base calls reached 99.9%. The Illumina reads were then used to further correct the assembly by mapping them to the polished HGAP assembly using Bowtie and PILON [82]. Assemblies were further filtered to remove contigs with greater than 10% of ambiguous or masked bases to produce the final assemblies. We attempted to place these smaller contigs into the assemblies based on syntenic alignments with complete chromosomes from other strains, but were only able to place one unambiguously, based on a continuous alignment greater than 50 Kb (Additional file 9: Table S4; contig 3 in NBRC31671). Remaining contigs containing telomeric repeats were either very short (< 110,000 kb) and/or contained masked sequence or high repeats and lacked alignments > 50 kb to any of the major contigs. Gene annotations were called using MAKER 2.28 [40] using input of protein sequences from *T. inflatum*, *Fusarium graminearum*, and *Neurospora crassa* as protein evidence and the assembled transcriptomes for each strain provided as ESTs. The fungal specific library in RepeatMasker was used for masking and to identify characterized fungal repeats and TEs in the genomes [83]. Two additional de-novo approaches, RepeatScout [84] and a custom pipeline using numer [85] were also used to identify repeat regions. SM genes and clusters were identified from the raw genome sequence using antiSMASH 4.0 [44] and a custom pipeline using custom HMMER models developed for fungal A, T, and C domains of NRPSs and KS domains of PKSs by Bushley et al. (2013).

### Phylogenetic analyses of clusters and analyses of selection

Phylogenetic analyses of NRPSs, PKSs, and TSs was conducted by extracting the A domain, KS domain, and TS or cyclase domains, respectively, from these genes. These domains were aligned with mafft version 7.221 [86] and maximum likelihood phylogenies were constructed in RAxML using the best fit RTREV + F for AMP domains and WAG for PKS and TS amino acid substitution models and automatically estimated numbers of bootstrap replicates [42, 87]. Related outgroup domains for NRPS (acetyl-CoA synthase, acyl-CoA ligase), PKS (fatty acid synthases), and the top blast hits to fungal genomes for TSs were included in each tree. For peptaibiotic NRPSs A domains from the Tex1 peptaibol synthase from *Trichoderma virens* were included [49]. For the AF-like cluster (cluster D), Protein ortho [88] was used to assess the conservation of cluster genes across all strains of *T. inflatum,* and syntenic arrangements of genes were inspected in mauve alignments. For the *T. inflatum* AF-like cluster, we also searched a local database of 340 Pezizomycotina fungal proteomes for homologs of cluster genes using Usearch version 8.0.1517 [89], retaining sequences with at least 50% amino acid similarity and an e-value of 10^− 3^*.* Preliminary gene trees were generated in fasttree version 2.1 [89] after aligning with mafft version 7.221 [86], and trimming the alignment with Trimal version 1.4 [90]. A subset of preliminary trees was generated after using usearch agglomerative clustering to reduce datasets to 50,000–2 million characters. Alignments were then reduced to include 50–150 sequences descending from a well-supported (> 95% fasttree support) node containing the *T. inflatum* cluster genes, and reanalyzed in RAxML version 8.2.9 [87] using the best amino acid substitution model according to the Bayesian Information Criterion, and 100 bootstrap replicates. Homologous clusters were identified when homologous genes were separated by no more than 6 intervening genes in the target genome [25]. A codon alignment for each of four partitions of the *simA* gene were created using pal2nal [91]. The dN/dS ratio and statistical tests for positive selection were performed with HYPHY using the fixed-effects likelihood (FEL) method [92] implemented on the data-monkey server (https://www.datamonkey.org/).

### Single nucleotide polymorphism variant calling and phylogeny reconstruction

Genomic variants, including single nucleotide polymorphisms (SNPs), indels, and structural variations, were identified through a pipeline that includes whole genome alignment and variant calling. The whole genome alignment was generated via Mugsy (PMID: 21148543) that utilizes NUCmer (PMID: 14759262) to produce all-against-all pairwise alignment and SeqAn (PMID: 18184432) to construct an alignment graph that guides the final assembly of the multi-genome alignment. The resulted MAF (multi-alignment format) file was analyzed by MafFilter (PMID: 24447531) to filter, refine and extract variants. Customized scripts were used to convert the output txt file into fasta format for downstream analysis. The fasta output format from the SNP variant calling pipeline, containing 284,848 SNPs, was used to create a maximum likelihood phylogeny in RAxML using the CAT model.

### Repeat identification and correlation analyses

Due to the limited knowledge of fungal repeats, three methods were used to define repetitive regions in the fungal genomes. The first method utilized known fungal repeats in the fungal repeat database bundled within the program RepeatMasker [83]. The second method first used RepeatScout to learn a library of repeated sequences in the six genomes through kmer alignment-and-extension strategy and then used RepeatMasker to scan the fungal genome for these de-novo identified repeats. The last method used a documented pipeline using genome aligner NUCmer (http://mummer.sourceforge.net/manual/#identifyingrepeats) within the MUMmer 3.0 package to identify similar regions within the genomes [85]. Correlation of repeats with SBMC was performed with the R-package GenometriCorr [93].

### Whole genome alignment and identification of structural rearrangements

To identify chromosome scale structural variants such as translocations, pair-wise whole-genome alignments of each strain aligned to CBS714.70 were created using NUCmer (PMID: 14759262) with default parameters. For visualization purpose, Mauve (PMID: 20593022) alignments using default parameters were generated for each chromosome across all strains and for selected genomic regions. To identify large inversions, a customized script was used to identify genome rearrangements between CBS714.70 and other strains (https://github.com/yingzhang121/Tolypocladium_inflatum_paper). This script scans NUCmer-aligned blocks along a reference genome and reports all alignment blocks that are aligned in the 3′ end to 5′ end orientation. By default, the reference and query sequences align from 5′ end to 3′ end. To examine whether inversion structural rearrangements followed the SNP phylogeny, we created a distance based clustering dendrogram based on an inversion index that measured the proportion of each genome involved in inversions in pairwise comparisons between strains. The inversion index was calculated as the average fraction of inverted base pairs in pairwise comparisons between genomes [(inverted_base_in_genome_1/size_of_genome_1) + (inverted_base_in_genome_2/size_of_genome_2)/2]. The R package gplots (https://CRAN.R-project.org/package=gplots) was used to generate the heatmap representation of rearrangement indices. Assemblytics [43] was used to identify smaller-scale structural variants within each genome.

### Hi-C analysis of structural variation

Hyphae of *T. inflatum* strain NRRL8044 were crosslinked with formaldehyde, digested with AflIII, ligated, and then sequenced on the Next-Seq Illumina platform to generate a total of 595 Mbp of data and 7,435,369 PE80 Hi-C read pairs that were used for the project [94]. To detect structural variation between the NRRL8044 and CBS714 genomes, Hi-C data of NRRL8044 was mapped onto the CBS714 genome and regions where the mapped Hi-C interaction frequencies showed significant differences from expected frequencies were identified. The CBS714 pacbio genome assembly was first broken into 20 kb bins. Hi-C data from the NRRL8044 sample was mapped to the genome of the CBS714 sample using “bwa mem -5” [95] and filtered using “samtools -F 2316” [96] to eliminate Hi-C read pairs where one or more reads were unmapped, or where one of the reads was not a primary alignment or had secondary alignments. Hi-C interaction counts were calculated for each pair of bins in the 20 kb-resolution CBS714 assembly and a heatmap of these interaction counts was generated using a custom R script (available at https://github.com/phasegenomics/fungus_heatmap). Three pairs of chromosomal regions showing possible translocations were identified visually, and the Hi-C linkage counts in these candidate regions were normalized by the number of restriction sites in the 20 kb genome bins. The average Hi-C linkage density per 20 kb in these pairs were compared to the average linkage density per 20 kb elsewhere in the same pair of chromosomes and deemed to be consistent with an inter-chromosomal translocation event if the ratio of these averages was at least fivefold greater than the average linkage density between the chromosomes. All three candidate regions were found to be consistent with these events. Additionally, to determine whether these results were associated with some kind of bias or unusual feature of the Hi-C data, the same analysis was performed using the NRRL8044 Hi-C data mapped to the NRRL8044 reference assembly and no such possible translocations were observed.

## Additional files

Additional file 1: **Table S1.** PacBio assemblies of each strain. (XLXS 13.9kb)

Additional file 2: **Figure S1.** Mauve alignments of each chromosome showing large inversions. (PDF 930 kb)

Additional file 3: **Figure S2.** Hi-C plot mapping NRRL8044 Hi-C data to the NRRL8044 assembly supports assembly of two unitigs to form chromosome 6. (PDF 499 kb)

Additional file 4: **Table S2.** Large inversions identified with NUCmer. (XLXS 21.8 kb)

Additional file 5: **Table S3.** Small-scale structural variants characterized with Assemblytics. (DOCX 45.9 kb)

Additional file 6: **Figure S3.** MUMmer plots of complete assemblies for all strains against the reference strain CBS714.70. (PDF 490 kb)

Additional file 7: **Figure S4.** Hi-C evidence for major translocation events in NRRL8044. (PDF 582 kb)

Additional file 8: **Figure S5.** Mapping of PacBio reads to the translocation junction. (PDF 2.89 Mb)

Additional file 9: **Table S4.** Secondary metabolite clusters found in six *T. inflatum* strains. (XLXS 17.5 kb)

Additional file 10: **Figure S6.** NRPS A domain phylogenetic tree. (PDF 13.5 kb)

Additional file 11: **Figure S7.** PKS KS domain phylogenetic tree. (PDF 12.17 Mb)

Additional file 12: **Figure S8.** Terpene synthase and terpene cyclase phylogenetic trees. (PDF 19.0 Mb)

Additional file 13: **Table S5.** Genome-wide correlation analyses of transposable elements with secondary metabolite clusters. (XLXS 18.9 kb)

Additional file 14: **Figure S9.** Shared and divergent gene content of clusters 27 and E. (PDF 653 kb)

Additional file 15: **Figure S10.** Phylogeny of A-domains of peptaibiotic NRPSs from *T. inflatum, T. ophioglossoides*, and *T. virens*. (PDF 25.0 Mb)

Additional file 16: **Figure S11.** Mauve alignment of peptaibiotic clusters 1, 10 and 42. (PDF 1.25 Mb)

Additional file 17: **Table S6.** Blast hits of the Cluster D genes to *A. flavus* genome and NCBI nr database (XLSX 15.3 kb)

Additional file 18: **Figure S12.** RAxML trees for homologs of each gene within *T. inflatum* CBS567.84 and CBS824.70 cluster D datamined from a database of 340 ascomycete fungi. (PDF 61.4 Mb)

Additional file 19: **Table S7.** Ortholog analysis of proteomes of all *T. inflatum* strains to cluster D genes. (XLSX 15 kb)

Additional file 20: **Table S8.** Selection in cyclosporin synthase. (*simA*) gene (XLSX 15.2 kb)

Additional file 21: **Figure S13.** Mauve alignments of conserved SMBGCs. (PDF 2.19 Mb)

## Abbreviations

A: Adenylation
AF: Aflatoxin
C: Condensation
CsA: Cyclosporin A
DMATs: Dimethylallyl tryptophan synthases
DOTH: Dothistromin
HGT: Horizontal gene transfer
KS: Ketosynthase
L75: >75% of the total genome size
LD: Linkage disequilibrium
NRPSs: Non-ribosomal peptide synthetases
P450s: P450 monooxygenases
PKSs: Polyketide synthases
SM: Secondary Metabolite
SMBGCs: Secondary metabolite biosynthetic gene clusters
SNPs: Single nucleotide polymorphisms
ST: Sterigmatocystin
T: Thiolation
TEs: Transposable elements
TS: Terpene synthase
