# Supplementary material for: Chromosome rearrangements shape the diversification of secondary metabolism in the cyclosporin producing fungus Tolypocladium inflatum
Source: BMC Genomics. 2019 Feb 7;20:120. doi: 10.1186/s12864-018-5399-x (PMC6367777; doi:10.1186/s12864-018-5399-x)
Supplement: Supplementary file 2 — Figure S1. Mauve alignments of each chromosome showing large inversions. (PDF 930 kb) [file 12864_2018_5399_MOESM2_ESM.pdf]

Figure S1. Mauve sequence alignments of the six *T. inflatum* chromosomes against reference strain CBS714.70 chromosome with the sequence coordinates on top. The end of chromosome six in strain NRRL8044 (orange segment) shows the 800 Kb translocated region from chromosome 2. Regions containing telomeric repeats in CBS714 are shown in grey on the end of each chromosome cartoon (not to scale). The chromosome for each strain is shown as a black line. Synteny and inversions are shown as colored syntenic blocks either above the black line, indicating synteny in the forward (sense) orientation relative to CBS714.70, or below the line indicating an inverse (antisense) orientation. The strength and height of the colored lines within each colored syntenic block represents the average sequence conservation. Areas without a colored block indicate regions not sharing synteny with any other strains. Secondary metabolite clusters are numbered according to those found in the reference 714.70 genome and colored by metabolite class (red=NRPS, blue=T1PKS, green=T1PKS-NRPS, purple=terpene, yellow=Indole-terpene, and orange=other). Cluster boxes labeled with a letter indicate clusters not found in the reference genome. Small black lines represent transposable elements. Red vertical lines that bisect the horizontal middle black line mark the beginning and end of unitigs that were not joined in the PacBio assembly; chromosome 6 of isolates 31671, 31975, 824.70 and 8044 is comprised of two unitigs that align to chromosomes 6 in 714.70 and their connection into a single chromosome and relative orientation is supported in 8044 by Hi-C data.

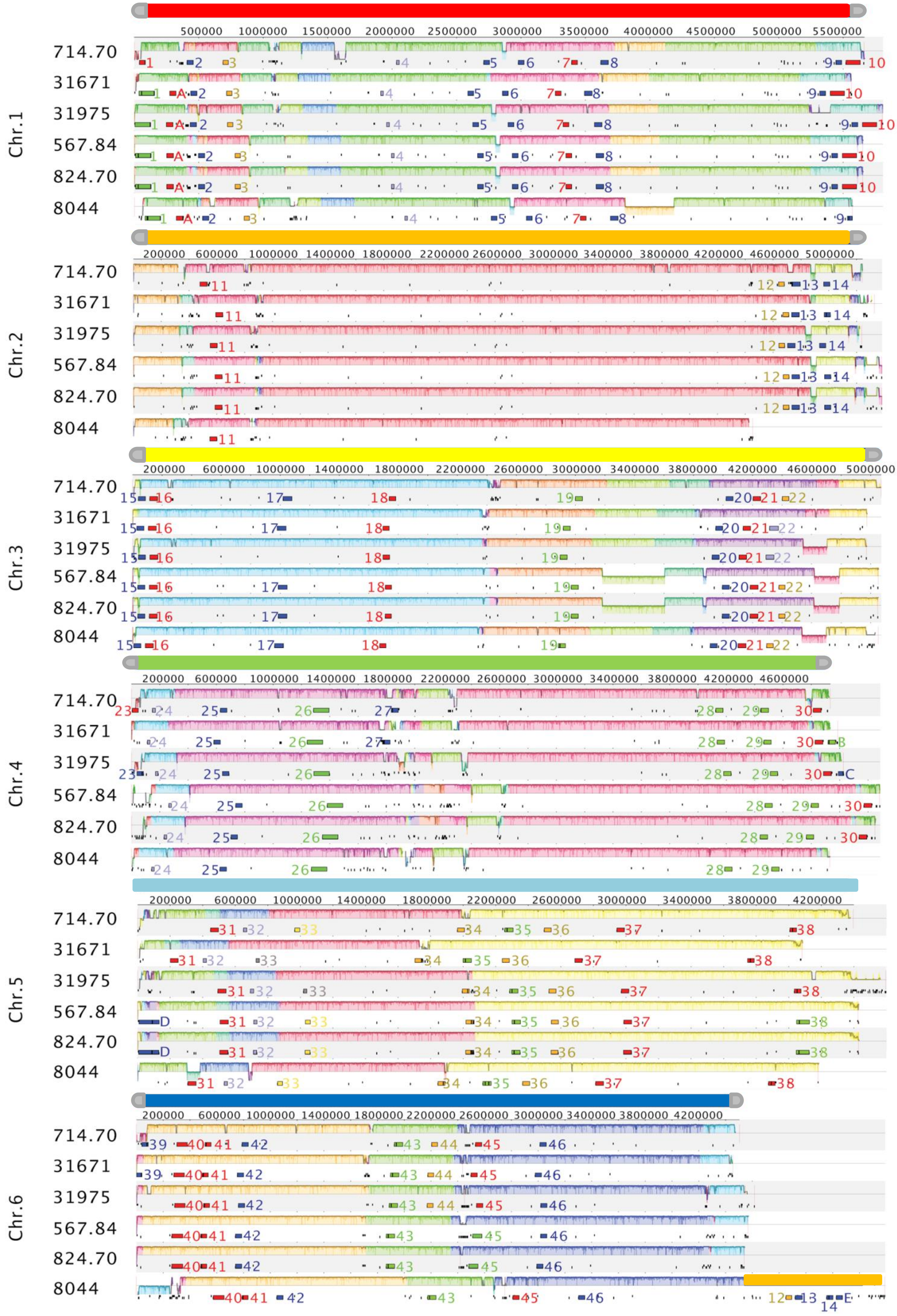
Chr.1
500000 1000000 1500000 2000000 2500000 3000000 3500000 4000000 4500000 5000000 5500000
714.70
31671
31975
567.84
824.70
8044
Chr.2
200000 600000 1000000 1400000 1800000 2200000 2600000 3000000 3400000 3800000 4200000 4600000 5000000
714.70
31671
31975
567.84
824.70
8044
Chr.3
200000 600000 1000000 1400000 1800000 2200000 2600000 3000000 3400000 3800000 4200000 4600000 5000000
714.70
31671
31975
567.84
824.70
8044
Chr.4
200000 600000 1000000 1400000 1800000 2200000 2600000 3000000 3400000 3800000 4200000 4600000
714.70
31671
31975
567.84
824.70
8044
Chr.5
200000 600000 1000000 1400000 1800000 2200000 2600000 3000000 3400000 3800000 4200000
714.70
31671
31975
567.84
824.70
8044
Chr.6
200000 600000 1000000 1400000 1800000 2200000 2600000 3000000 3400000 3800000 4200000
714.70
31671
31975
567.84
824.70
8044
