# Supplementary material for: Chromosome rearrangements shape the diversification of secondary metabolism in the cyclosporin producing fungus Tolypocladium inflatum
Source: BMC Genomics. 2019 Feb 7;20:120. doi: 10.1186/s12864-018-5399-x (PMC6367777; doi:10.1186/s12864-018-5399-x)

Figure S2. Post-scaffolding heatmap of 8044 Hi-C data mapped to 10kb bins of the 8044 PacBio assembly. Dashed black lines correspond to chromosome boundaries and centromeres depicted as high-density dots in the middle of chromosomes. Strong Hi-C signal is seen along the diagonal. The blue lines within chromosome 6 correspond to boundaries of the two unitigs 43 and 34 that comprise chromosome six. The strong signal along the diagonal supports the relative orientation of these two unitigs forming chromosome 6 in the assembly.

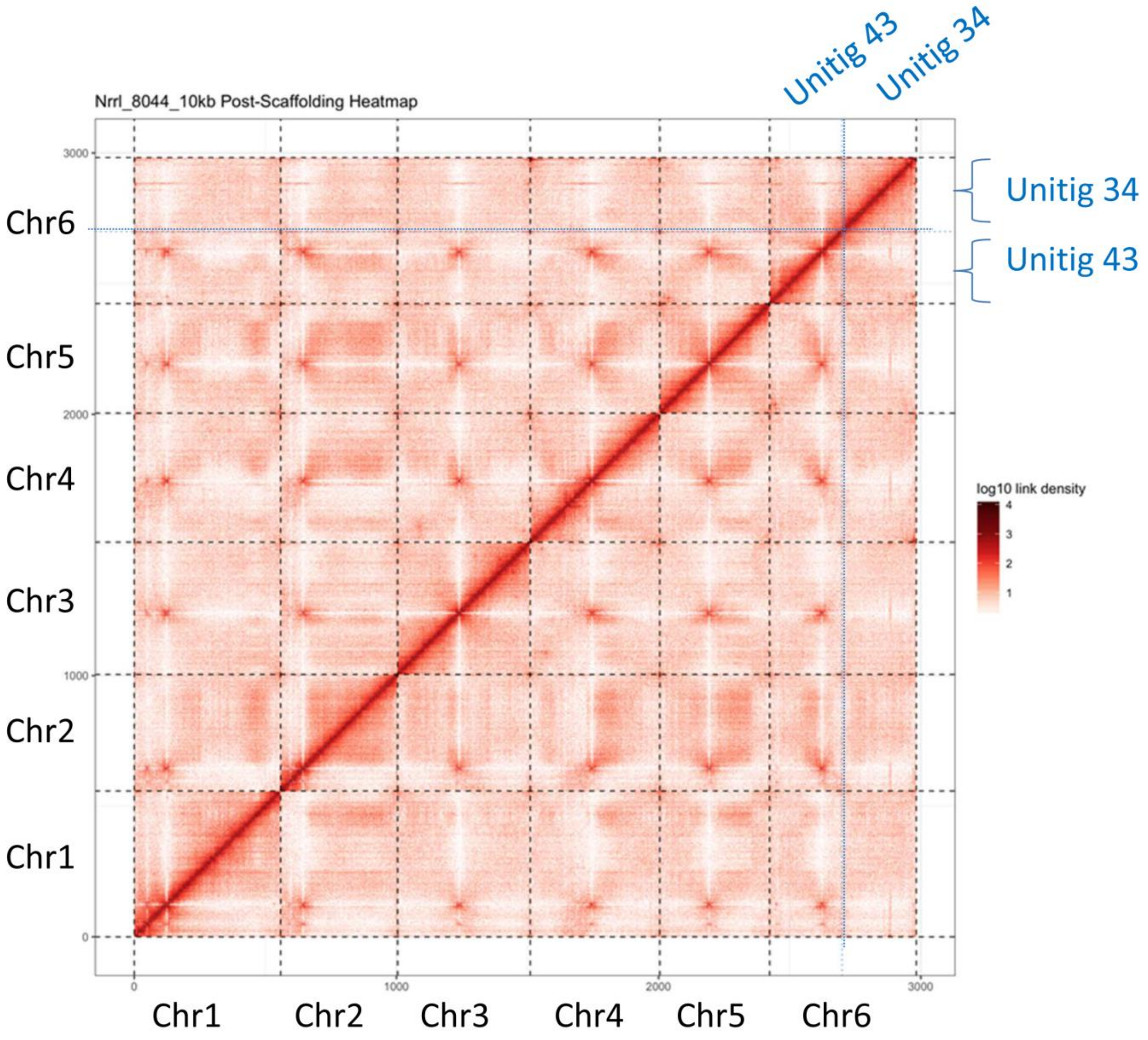

Supplement: Supplementary file 3 — Figure S2. Hi-C plot mapping NRRL8044 Hi-C data to the NRRL8044 assembly supports assembly of two unitigs to form chromosome 6. (PDF 499 kb) [file 12864_2018_5399_MOESM3_ESM.pdf]
