# Supplementary material for: Chromosome rearrangements shape the diversification of secondary metabolism in the cyclosporin producing fungus Tolypocladium inflatum
Source: BMC Genomics. 2019 Feb 7;20:120. doi: 10.1186/s12864-018-5399-x (PMC6367777; doi:10.1186/s12864-018-5399-x)
Supplement: Supplementary file 5 — Table S3. Small-scale structural variants characterized with Assemblytics. (DOCX 45.9 kb) [file 12864_2018_5399_MOESM5_ESM.docx]

| Additional file 3: Table S3 Small-scale structural variants detected with Assemblytics | | | | | |
| --- | --- | --- | --- | --- | --- |
|  | 31671 | 31975 | 567 | 824 | 8044 |
| Insertions |  |  |  |  |  |
| 50-500 bp | 100 | 165 | 163 | 162 | 164 |
| 500-10000 bp | 26 | 38 | 32 | 32 | 39 |
| Total | 126 | 203 | 195 | 194 | 203 |
| Deletions |  |  |  |  |  |
| 50-500 bp | 109 | 138 | 152 | 152 | 161 |
| 500-10000 bp | 55 | 48 | 51 | 51 | 51 |
| Total | 164 | 186 | 203 | 203 | 212 |
| Tandem_expansions |  |  |  |  |  |
| 50-500 bp | 12 | 12 | 7 | 7 | 9 |
| 500-10000 bp | 3 | 2 | 2 | 2 | 3 |
| Total | 15 | 14 | 9 | 9 | 12 |
| Tandem_contractions |  |  |  |  |  |
| 50-500 bp | 11 | 8 | 8 | 8 | 7 |
| 500-10000 bp | 0 | 0 | 0 | 0 | 0 |
| Total | 11 | 8 | 8 | 8 | 7 |
| Repeat_expansions |  |  |  |  |  |
| 50-500 bp | 19 | 59 | 59 | 59 | 53 |
| 500-10000 bp | 47 | 99 | 111 | 111 | 123 |
| Total | 66 | 158 | 170 | 170 | 176 |
|  |  |  |  |  |  |
| Repeat_contractions |  |  |  |  |  |
| 50-500 bp | 16 | 65 | 65 | 65 | 64 |
| 500-10000 bp | 48 | 91 | 96 | 96 | 83 |
| Total | 64 | 156 | 161 | 161 | 147 |
| Total structural variants | 446 | 725 | 746 | 745 | 757 |
