# Supplementary material for: Chromosome rearrangements shape the diversification of secondary metabolism in the cyclosporin producing fungus Tolypocladium inflatum
Source: BMC Genomics. 2019 Feb 7;20:120. doi: 10.1186/s12864-018-5399-x (PMC6367777; doi:10.1186/s12864-018-5399-x)

Figure S3 MUMmer plots of each *T. inflatum* strain (x-axis) against CBS714.70 (y-axis) . High levels of synteny were observed for nearly all strains with the exception of NRRL8044 (right), which showed evidence of a large 800 Kb translocation (A;green circle) from chromosome 2 to chromosome 6 and a smaller 200 Kb translocation and inversion (B;blue circle) from one end of chromosome 6 to the other end.

NRRL8044

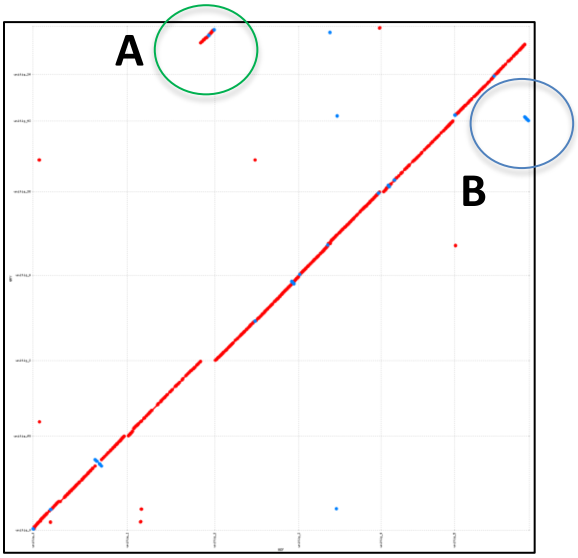

CBS567.84

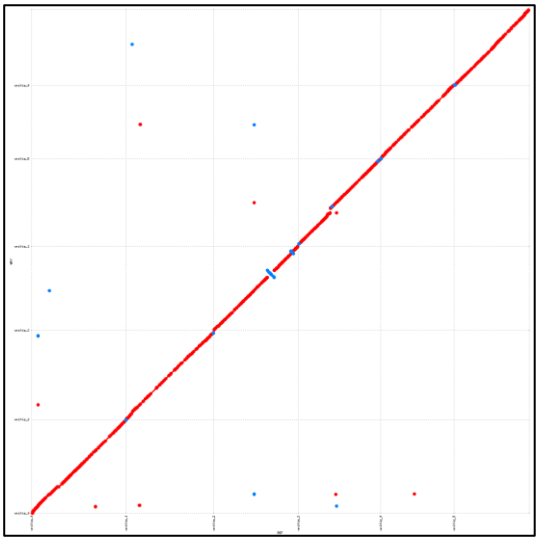

CBS824.70

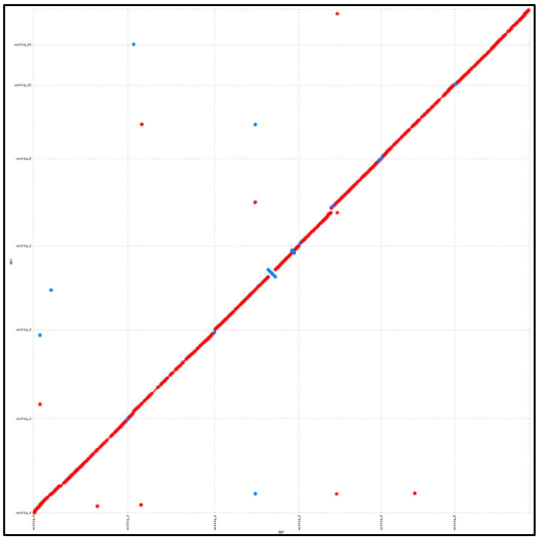

NBRC31671

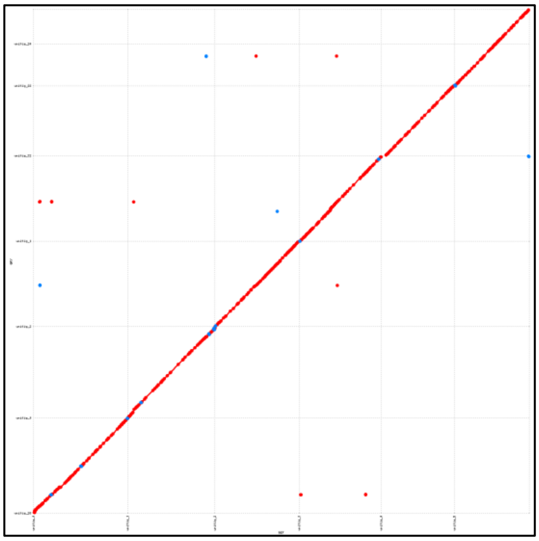

NBRC31975

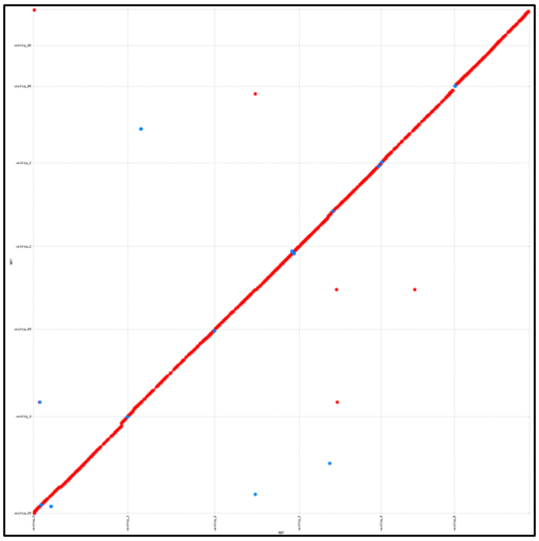

Supplement: Supplementary file 6 — Figure S3. MUMmer plots of complete assemblies for all strains against the reference strain CBS714.70. (PDF 490 kb) [file 12864_2018_5399_MOESM6_ESM.pdf]
