# Supplementary material for: Chromosome rearrangements shape the diversification of secondary metabolism in the cyclosporin producing fungus Tolypocladium inflatum
Source: BMC Genomics. 2019 Feb 7;20:120. doi: 10.1186/s12864-018-5399-x (PMC6367777; doi:10.1186/s12864-018-5399-x)
Supplement: Supplementary file 7 — Figure S4. Hi-C evidence for major translocation events in NRRL8044. (PDF 582 kb) [file 12864_2018_5399_MOESM7_ESM.pdf]

Figure S4 Hi-C enrichment analysis in regions surrounding translocations in NRRL8044.

Generally, translocation events between two species or individual are visible in Hi-C data as regions where the observed Hi-C data from one individual significantly differs from the statistical expectations for those data when mapped to a second individual [3-5]. Statistical expectations are derived from both the polymer dynamics of long DNA molecules as well as the biological dynamics of chromatin features in the nucleus, such as centromeres, telomeres, or chromatin organization patterns such as the Rabl configuration [6-10]. Common statistical expectations include patterns such as significant enrichment for Hi-C interactions between loci on the same chromosome as opposed to loci on different chromosomes ("intrachromosome enrichment"), and a decay in Hi-C interation frequency that follows a power law with respect to the number of base pairs separating two loci on the same chromosome ("proximity enrichment"). Violations of these statistical expectations can identify candidate structural variants or can be used to confirm structural variants suggested by other means.

We used Hi-C data to confirm three structural variants suggested through mummer alignments by examining the intrachromosome enrichment for two interchromosomal translocations and the proximity enrichment for one intrachromosomal translocation. In each case, the putative translocation was identified by examining the Hi-C link densities (which are simply the Hi-C link counts normalized by the number of Hi-C restriction sites) between pairs of loci in the genome at 20kbp resolution, and comparing it to background link density interactions for the chromosome(s) containing the loci included in the putative translocation. The ratio of the two Hi-C link densities was calculated and deemed to confirm the putative translocation if it the link density inside the translocation was at least five times the background density level.

The translocation events are shown and numbered in Figure 3, reproduced below for convenience.

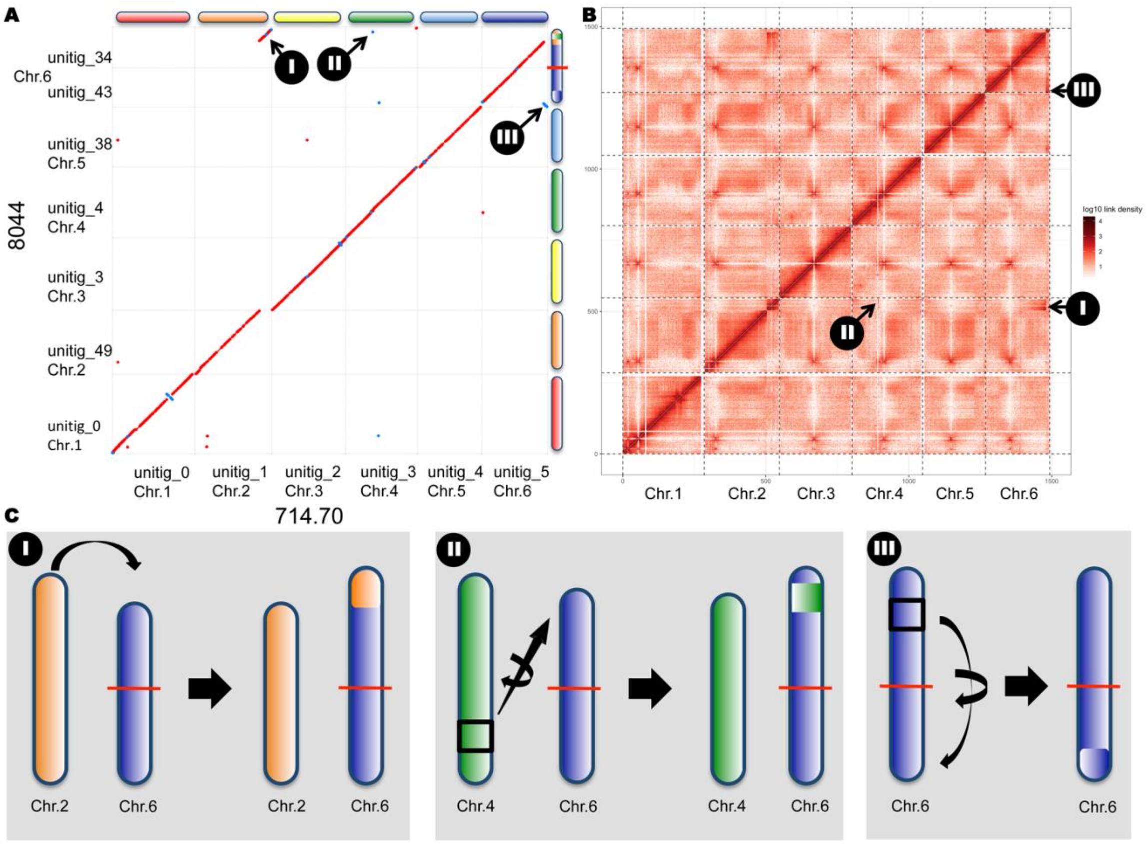

Figure 3. Chromosomal translocations in strain 8044. A) MUMmer plot in which 714.70 was used as the reference genome (x-axis) and 8044 as the query (y-axis). Red dots aligning with the diagonal axis indicate syntenic aligned sequence, while blue dots represent an inversion. Dots off the diagonal indicate a translocation or exchange of DNA between chromosomes. Homologous chromosomes in different strains along each axis are color coded (Chr.1=red, Chr. 2=orange, Chr.3=yellow, Chr.4=green, Chr.5=light blue, Chr. 6=dark blue). Genome rearrangements observed in 8044 relative to 714.70 include **I)** A translocation of a ~800 Kb segment of DNA from one end of chromosome 2 onto the end of chromosome 6 (orange segment); **II)** Both translocation and inversion of a small segment of DNA containing a PKS and several accessory genes from chromosome 4 to the large translocated region (I) on the end of chromosome 6 (green segment); **III)** An intrachromosomal inversion/translocation from the right arm of chromosome 6 to the opposite (left) arm of the same chromosome. The red line bisecting chromosome 6 indicates the boundary of two unitigs (34 and 43) that comprise chromosome 6. B) Plot of 8044 Hi-C data mapped to the 714.70 assembly shows hotspots that deviate from the diagonal and support these three translocation events (SFigure 4). C) Cartoon of three translocation events.

The following table provides additional details on these translocations:

Translocation	Type	Translocated Region Start	Translocated Region End	Translocation Destination Coordinate	Hi-C Density In Feature	Background Hi-C Density	Enrichment Score

**I**	Interchrom.	Chr 2 4,410kbp ±10kbp	Chr 2 5,230kbp ±10kbp	Chr 6 4,210kbp ±10kbp	412.38	81.11	5.08
**II**	Interchrom., inversion	Chr 4 1,850kbp ±10kbp	Chr4 1,870kbp ±10kbp	Chr 6 5,030 ±10kbp (via intermediate translocation to Chr 2 5,230 ±10kbp in **I**)	1750.77	145.30	12.05
**III**	Intrachrom., inversion	Chr 6 4,250kbp ±10kbp	Chr 6 4,450kbp ±10kbp	Chr 6 0kbp	750.37	144.68	5.19

Table 1. Translocation details and related Hi-C linkage density data supporting the existence of the translocation. Translocation coordinates were identified to within 10kbp by examining Hi-C interaction data at 20kbp resolution and using the center of the identified 20kbp regions as the coordinate. Enrichment scores were calculated as the ratio of the Hi-C link density within putative translocations as compared to the interchromosomal or intrachromosomal background depending on the type of the translocation. Note that translocation II has a non-zero size, i.e., the existence of the margin of error does not leave open the possibility that the event is of size 0.

Of particular interest, examining the Hi-C linkage density data shows that translocations I and II were related to each other. Translocation II's destination was to the region of Chr 2 which was itself translocated to Chr 6 as part of translocation I. This is visible in the Hi-C heatmap in panel B of Figure 4. Translocation II appears as an enhanced Hi-C linkage density between a region of Chr 4 and a region of Chr 2 consistent with the translocation of a small region from Chr 4 to Chr 2. These regions of Chr 2 also show enhanced Hi-C linkage density with a region of Chr 6 consistent with the translocation of those regions of Chr 2 to Chr 6. Thus, because there is enhanced Hi-C linkage density among three different regions, and the source and destination of translocations among these regions is discernable from the Hi-C linkage density patterns, we can conclude that the region involved in translocation II was subsequently included in translocation I as CBS714 and NRRL8044 diverged evolutionarily.

Citations

1. Li H. and Durbin R. (2010) Fast and accurate long-read alignment with Burrows-Wheeler Transform. Bioinformatics, Epub. [PMID: 20080505]
2. Li H., Handsaker B., Wysoker A., Fennell T., Ruan J., Homer N., Marth G., Abecasis G., Durbin R. and 1000 Genome Project Data Processing Subgroup (2009) The Sequence alignment/map (SAM) format and SAMtools. Bioinformatics, 25, 2078-9. [PMID: 19505943]
3. Martin P., McGovern A., Orozco G., Duffus K., Yarwood A., Schoenfelder S., Cooper N.J., Barton A., Wallace C., Fraser P., Worthington J., and Eyre S. (2015) Capture Hi-C reveals novel candidate genes and complex long-range interactions with related autoimmune risk loci. Nature Communications 6 Art. 10069, http://dx.doi.org/10.1038/ncomms10069
4. Harewood L, Kishore K, Eldridge MD, Wingett S, Pearson D, Schoenfelder S, et al. Hi-C as a tool for precise detection and characterisation of chromosomal rearrangements and copy number variation in human tumours. Genome Biol. 2017;18(1):125.
5. Dixon J., Xu J., Dileep V., Zhan Y., Song F., Le V.T., Yardimci G.G, Chakraborty A., Bann D.V., Wang Y., Clark R., Zhang L., Yang H., Liu T., Iyyanki S., An L., Pool C., Sasaki T., Mulia J.C.R., Ozadam H.,

Lajoie B.R., Kaul R., Buckley M., Lee K., Diegel M., Pezic D., Ernst C., Hadjur S., Odom D.T., Stamatoyannopoulos J.A., Broach J.R., Hardison R., Ay F., Noble W.S., Dekker J., Gilbert D.M., Yue F. An Integrative Framework For Detecting Structural Variations In Cancer Genomes. bioRxiv 119651; doi: https://doi.org/10.1101/119651

6. Varoquaux N., Liachko I., Ay F., Burton J.N., Shendure J., Dunham M.J., Vert J.P., Noble W.S. Accurate identification of centromere locations in yeast genomes using Hi-C. Nucleic Acids Res. 2015 Jun 23;43(11):5331-9. doi: 10.1093/nar/gkv424. Epub 2015. May 4. PubMed PMID: 25940625; PubMed Central PMCID: PMC4477656.
7. Nora E.P. Lajoie B.R. Schulz E.G. Giorgetti L. Okamoto I. Servant N. Piolot T. van Berkum N.L. Meisig J. Sedat J.et al. Spatial partitioning of the regulatory landscape of the X-inactivation centre Nature 2012 485 381 385
8. Dixon J.R. Selvaraj S. Yue F. Kim A. Li Y. Shen Y. Hu M. Liu J.S. Ren B. Topological domains in mammalian genomes identified by analysis of chromatin interactions Nature 2012 485 376 380
9. Mizuguchi T. Fudenberg G. Mehta S. Belton J.M. Taneja N. Folco H.D. FitzGerald P. Dekker J. Mirny L. Barrowman J.et al. Cohesin-dependent globules and heterochromatin shape 3D genome architecture in S. pombe Nature 2014 516 432 435
10. Marie-Nelly H. Marbouty M. Cournac A. Liti G. Fischer G. Zimmer C. Koszul R. Filling annotation gaps in yeast genomes using genome-wide contact maps Bioinformatics 2014 30 2105 2113
