# Supplementary material for: Chromosome rearrangements shape the diversification of secondary metabolism in the cyclosporin producing fungus Tolypocladium inflatum
Source: BMC Genomics. 2019 Feb 7;20:120. doi: 10.1186/s12864-018-5399-x (PMC6367777; doi:10.1186/s12864-018-5399-x)

SFigure 5. Alignment of PacBio corrected reads to the junction of the major translocation from chromosome 2 to chromosome 6 in NRRL8044 show continuous coverage over this region.

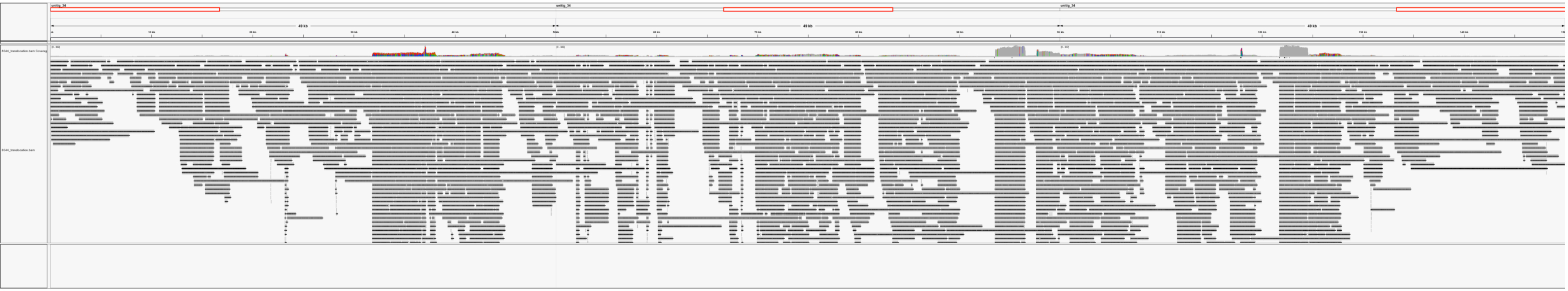

Supplement: Supplementary file 8 — Figure S5. Mapping of PacBio reads to the translocation junction. (PDF 2.89 Mb) [file 12864_2018_5399_MOESM8_ESM.pdf]
