# Supplementary material for: Chromosome rearrangements shape the diversification of secondary metabolism in the cyclosporin producing fungus Tolypocladium inflatum
Source: BMC Genomics. 2019 Feb 7;20:120. doi: 10.1186/s12864-018-5399-x (PMC6367777; doi:10.1186/s12864-018-5399-x)

Supplemental Figure 6. Phylogeny of adenylation (A) domains across six *T. inflatum* strains. Maximum likelihood phylogeny created in RAxML using the best-fit amino acid substitution model RTREV + F and an automatically optimized number of bootstrap replicates. A domains belonging to the cyclosporin cluster are color coded purple while A domains involved in biosynthesis of peptaibiotics are color coded blue.

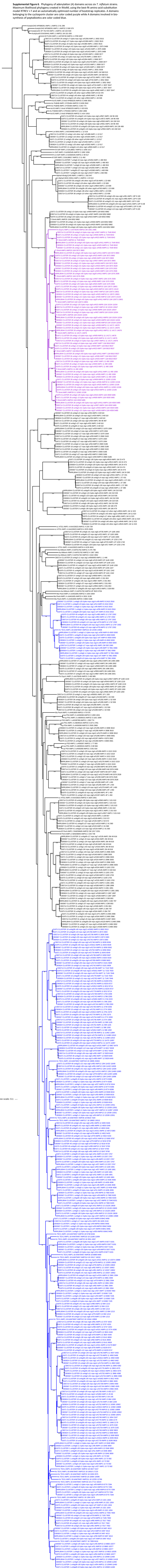

Supplement: Supplementary file 10 — Figure S6. NRPS A domain phylogenetic tree. (PDF 13.5 kb) [file 12864_2018_5399_MOESM10_ESM.pdf]
