# Supplementary material for: Chromosome rearrangements shape the diversification of secondary metabolism in the cyclosporin producing fungus Tolypocladium inflatum
Source: BMC Genomics. 2019 Feb 7;20:120. doi: 10.1186/s12864-018-5399-x (PMC6367777; doi:10.1186/s12864-018-5399-x)

**SFigure 7.** Phylogeny of ketosynthase (KS) domains of PKS genes across six *T. inflatum* strains. Maximum likelihood phylogeny created in RAxML using best-fit amino acid substitution model WAG and an automatically optimized number of bootstrap replicates. The phylogeny also includes outgroup KS domains from fatty acid synthases and top 50 BLAST hits in the ncbi nr database to the aflatoxin PKS *aflC*. Four strain unique KS domains belonging to the three PKSs found in cluster D are color coded red while the top BLAST hits to *aflC* are color coded blue.

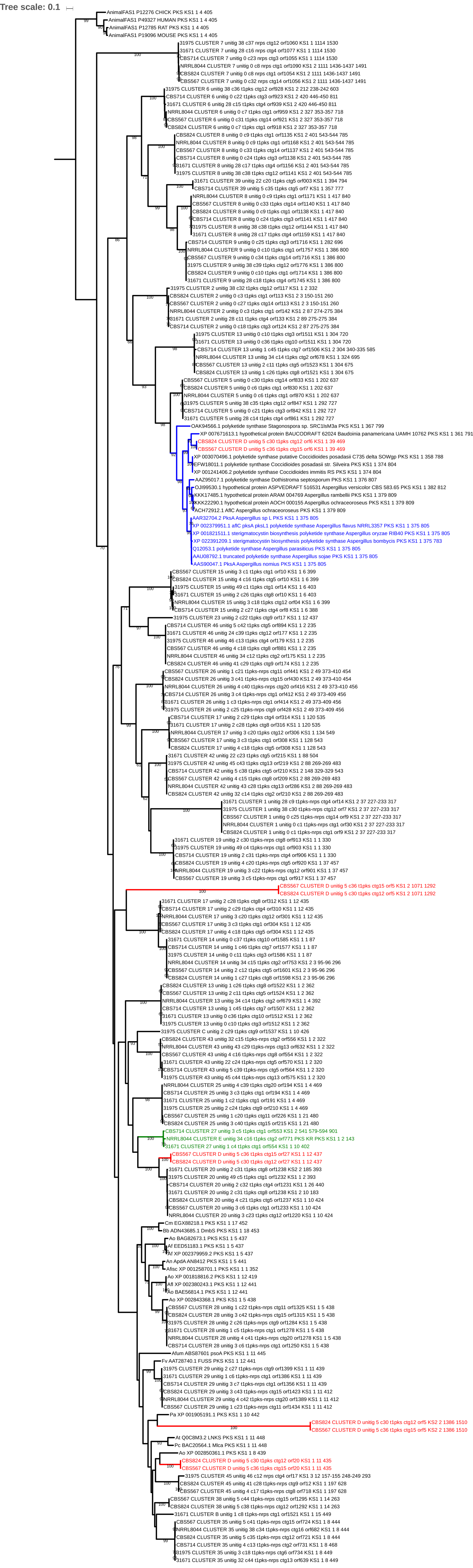

Supplement: Supplementary file 11 — Figure S7. PKS KS domain phylogenetic tree. (PDF 12.17 Mb) [file 12864_2018_5399_MOESM11_ESM.pdf]
