# Supplementary material for: Chromosome rearrangements shape the diversification of secondary metabolism in the cyclosporin producing fungus Tolypocladium inflatum
Source: BMC Genomics. 2019 Feb 7;20:120. doi: 10.1186/s12864-018-5399-x (PMC6367777; doi:10.1186/s12864-018-5399-x)
Supplement: Supplementary file 12 — Figure S8. Terpene synthase and terpene cyclase phylogenetic trees. (PDF 19.0 Mb) [file 12864_2018_5399_MOESM12_ESM.pdf]

SFigure 8. Phylogenetic trees of terpene biosynthetic genes. Distinct terpene synthase domains (phytoene synthase, PPI synthase, terpene cyclase, and terpene synthase) were extracted from core genes involved in terpene metabolism across the six *T. inflatum* strains. Each domain class was used in BLAST searched of the ncbi nr database (NCBI accession numbers shown) and the top 50 hits of fungal genes from each class were included. Phylogenetic analysis was performed using maximum likelihood in RAxML using the best-fit amino acid substation model (WAG) model and an automatically optimized number of bootstrap replicates. *T. inflatum* genes are color coded green.

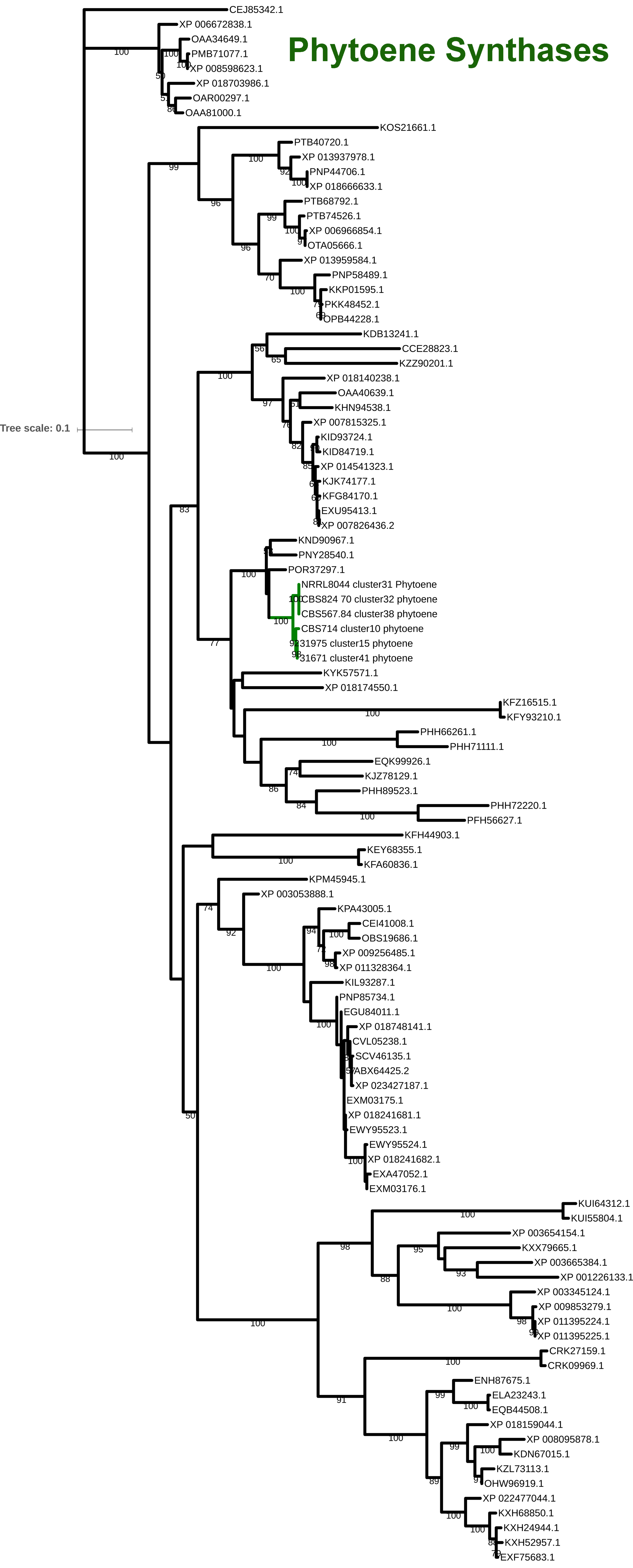

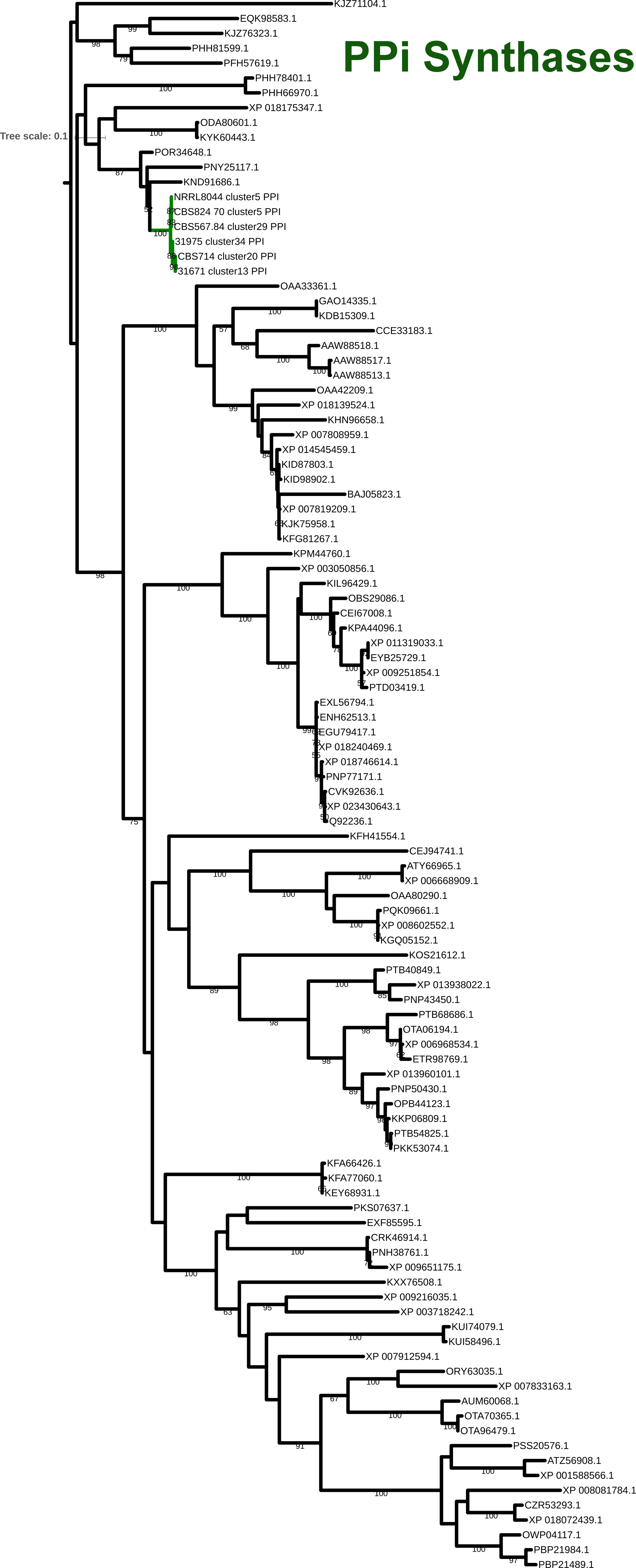

PPi Synthases
Tree scale: 0.1
KJZ71104.1
EQK98583.1
KJZ76323.1
PHH81599.1
PFH57619.1
PHH78401.1
PHH66970.1
XP 018175347.1
ODA80601.1
KYK60443.1
POR34648.1
PNY25117.1
KND91686.1
NRRL8044 cluster5 PPI
CBS824 70 cluster5 PPI
CBS567.84 cluster29 PPI
31975 cluster34 PPI
CBS714 cluster20 PPI
31671 cluster13 PPI
OAA33361.1
GAO14335.1
KDB15309.1
CCE33183.1
AAW88518.1
AAW88517.1
AAW88513.1
OAA42209.1
XP 018139524.1
KHN96658.1
XP 007808959.1
XP 014545459.1
KID87803.1
KID98902.1
BAJ05823.1
XP 007819209.1
KJK75958.1
KFG81267.1
KPM44760.1
XP 003050856.1
KIL96429.1
OBS29086.1
CEI67008.1
KPA44096.1
XP 011319033.1
EYB25729.1
XP 009251854.1
PTD03419.1
EXL56794.1
ENH62513.1
EGU79417.1
XP 018240469.1
XP 018746614.1
PNP77171.1
CVK92636.1
XP 023430643.1
Q92236.1
KFH41554.1
CEJ94741.1
ATY66965.1
XP 006668909.1
OAA80290.1
PQK09661.1
XP 008602552.1
KGQ05152.1
KOS21612.1
PTB40849.1
XP 013938022.1
PNP43450.1
PTB68686.1
OTA06194.1
XP 006968534.1
ETR98769.1
XP 013960101.1
PNP50430.1
OPB44123.1
KKP06809.1
PTB54825.1
PKK53074.1
KFA66426.1
KFA77060.1
KEY68931.1
PKS07637.1
EXF85595.1
CRK46914.1
PNH38761.1
XP 009651175.1
KXX76508.1
XP 009216035.1
XP 003718242.1
KUI74079.1
KUI58496.1
XP 007912594.1
ORY63035.1
XP 007833163.1
AUM60068.1
OTA70365.1
OTA96479.1
PSS20576.1
ATZ56908.1
XP 001588566.1
XP 008081784.1
CZR53293.1
XP 018072439.1
OWP04117.1
PBP21984.1
PBP21489.1

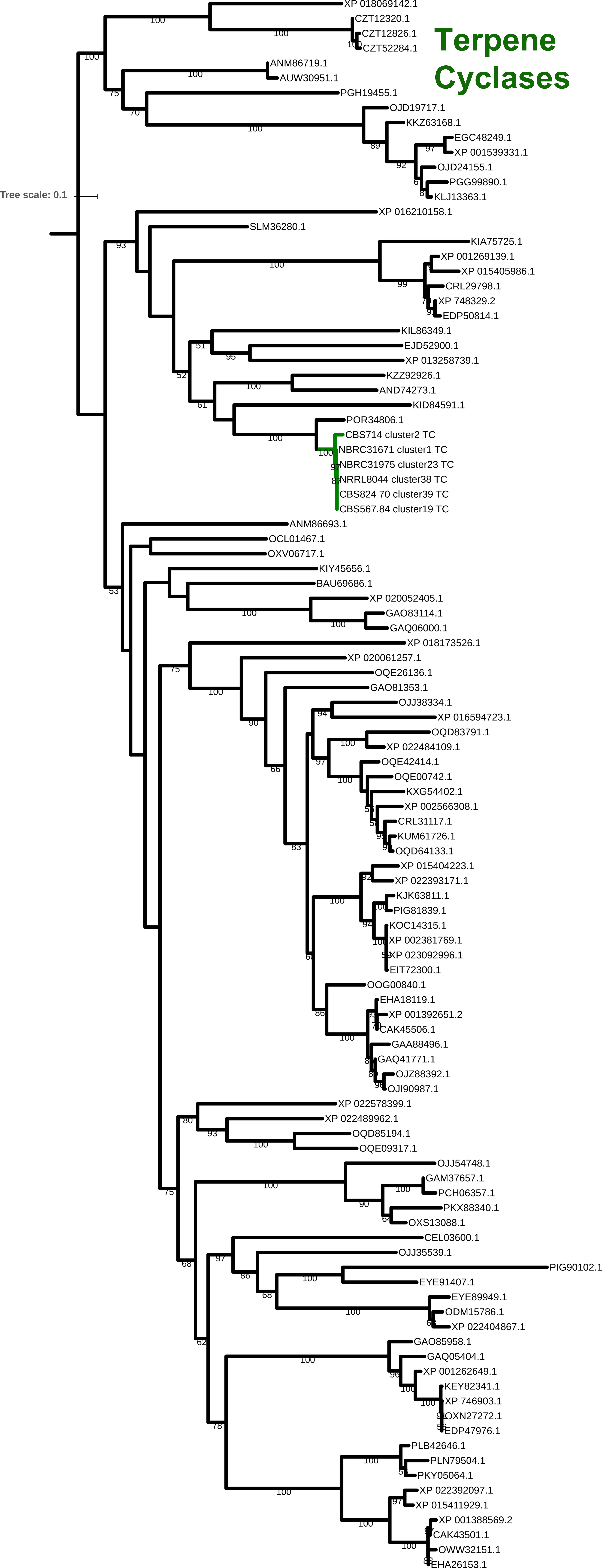

Terpene Cyclases
Tree scale: 0.1
XP 018069142.1
CZT12320.1
CZT12826.1
CZT52284.1
ANM86719.1
AUW30951.1
PGH19455.1
OJD19717.1
KKZ63168.1
EGC48249.1
XP 001539331.1
OJD24155.1
PGG99890.1
KLJ13363.1
XP 016210158.1
SLM36280.1
KIA75725.1
XP 001269139.1
XP 015405986.1
CRL29798.1
XP 748329.2
EDP50814.1
KIL86349.1
EJD52900.1
XP 013258739.1
KZZ92926.1
AND74273.1
KID84591.1
POR34806.1
CBS714 cluster2 TC
NBRC31671 cluster1 TC
NBRC31975 cluster23 TC
NRRL8044 cluster38 TC
CBS824 70 cluster39 TC
CBS567.84 cluster19 TC
ANM86693.1
OCL01467.1
OXV06717.1
KIY45656.1
BAU69686.1
XP 020052405.1
GAO83114.1
GAQ06000.1
XP 018173526.1
XP 020061257.1
OQE26136.1
GAO81353.1
OJJ38334.1
XP 016594723.1
OQD83791.1
XP 022484109.1
OQE42414.1
OQE00742.1
KXG54402.1
XP 002566308.1
CRL31117.1
KUM61726.1
OQD64133.1
XP 015404223.1
XP 022393171.1
KJK63811.1
PIG81839.1
KOC14315.1
XP 002381769.1
XP 023092996.1
EIT72300.1
OOG00840.1
EHA18119.1
XP 001392651.2
CAK45506.1
GAA88496.1
GAQ41771.1
OJZ88392.1
OJI90987.1
XP 022578399.1
XP 022489962.1
OQD85194.1
OQE09317.1
OJJ54748.1
GAM37657.1
PCH06357.1
PKX88340.1
OXS13088.1
CEL03600.1
OJJ35539.1
PIG90102.1
EYE91407.1
EYE89949.1
ODM15786.1
XP 022404867.1
GAO85958.1
GAQ05404.1
XP 001262649.1
KEY82341.1
XP 746903.1
OXN27272.1
EDP47976.1
PLB42646.1
PLN79504.1
PKY05064.1
XP 022392097.1
XP 015411929.1
XP 001388569.2
CAK43501.1
OWW32151.1
EHA26153.1

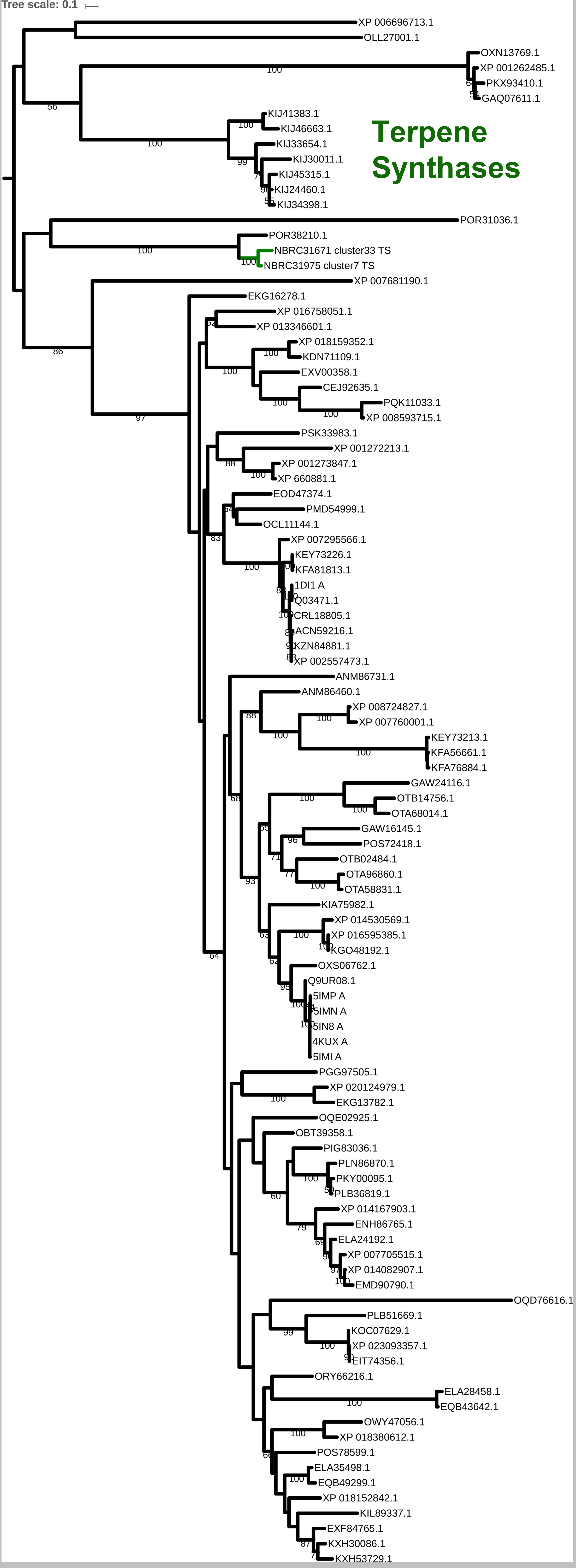

Tree scale: 0.1
Terpene Synthases
XP 006696713.1
OLL27001.1
OXN13769.1
XP 001262485.1
PKX93410.1
GAQ07611.1
KIJ41383.1
KIJ46663.1
KIJ33654.1
KIJ30011.1
KIJ45315.1
KIJ24460.1
KIJ34398.1
POR31036.1
POR38210.1
NBRC31671 cluster33 TS
NBRC31975 cluster7 TS
XP 007681190.1
EKG16278.1
XP 016758051.1
XP 013346601.1
XP 018159352.1
KDN71109.1
EXV00358.1
CEJ92635.1
PQK11033.1
XP 008593715.1
PSK33983.1
XP 001272213.1
XP 001273847.1
XP 660881.1
EOD47374.1
PMD54999.1
OCL11144.1
XP 007295566.1
KEY73226.1
KFA81813.1
1DI1 A
Q03471.1
CRL18805.1
ACN59216.1
KZN84881.1
XP 002557473.1
ANM86731.1
ANM86460.1
XP 008724827.1
XP 007760001.1
KEY73213.1
KFA56661.1
KFA76884.1
GAW24116.1
OTB14756.1
OTA68014.1
GAW16145.1
POS72418.1
OTB02484.1
OTA96860.1
OTA58831.1
KIA75982.1
XP 014530569.1
XP 016595385.1
KGO48192.1
OXS06762.1
Q9UR08.1
5IMP A
5IMN A
5IN8 A
4KUX A
5IMI A
PGG97505.1
XP 020124979.1
EKG13782.1
OQE02925.1
OBT39358.1
PIG83036.1
PLN86870.1
PKY00095.1
PLB36819.1
XP 014167903.1
ENH86765.1
ELA24192.1
XP 007705515.1
XP 014082907.1
EMD90790.1
OQD76616.1
PLB51669.1
KOC07629.1
XP 023093357.1
EIT74356.1
ORY66216.1
ELA28458.1
EQB43642.1
OWY47056.1
XP 018380612.1
POS78599.1
ELA35498.1
EQB49299.1
XP 018152842.1
KIL89337.1
EXF84765.1
KXH30086.1
KXH53729.1
