# Supplementary material for: Chromosome rearrangements shape the diversification of secondary metabolism in the cyclosporin producing fungus Tolypocladium inflatum
Source: BMC Genomics. 2019 Feb 7;20:120. doi: 10.1186/s12864-018-5399-x (PMC6367777; doi:10.1186/s12864-018-5399-x)
Supplement: Supplementary file 14 — Figure S9. Shared and divergent gene content of clusters 27 and E. (PDF 653 kb) [file 12864_2018_5399_MOESM14_ESM.pdf]

Figure S9. Alignment of gene content of gene cluster E in strain 8044 and cluster 27 in strains 31671 and 714.70, respectively. Arrows represent computationally annotated genes within each cluster, and colors indicate gene homology between the strains. Inlayed tables above and below figure are BLAST hits of each protein sequence corresponding to each gene. The 5' end of cluster 27 in 31671 is interrupted by a retrotransposon.

NRRL 8044 Gene No.	BLAST hit
8065	PREDICTED: collagen alpha-1(V) chain isoform X1 [*Xenopus tropicalis*]
8066	myb family transcription factor [*Hirsutella minnesotensis* 3608]
8067	Related to phospholipid-translocating ATPase [*Fusarium fujikuroi* IMI 58289]
8068	MFS general substrate transporter [*Aspergillus fijiensis* CBS 313.89]
8069	Putative polyketide synthase [*Diaporthe ampelina*]
8070	Beta,beta-carotene 15,15'-monooxygenase [*Tolypocladium ophioglossoides* CBS 100239]
8071	Hypothetical protein
8072	ERAD-associated E3 ubiquitin-protein ligase doa10 [*Tolypocladium ophioglossoides* CBS 100239]
8073	Nuclear distribution protein nudE [*Tolypocladium paradoxum*]
8074	Acetyl-CoA carboxylase [*Tolypocladium paradoxum*]

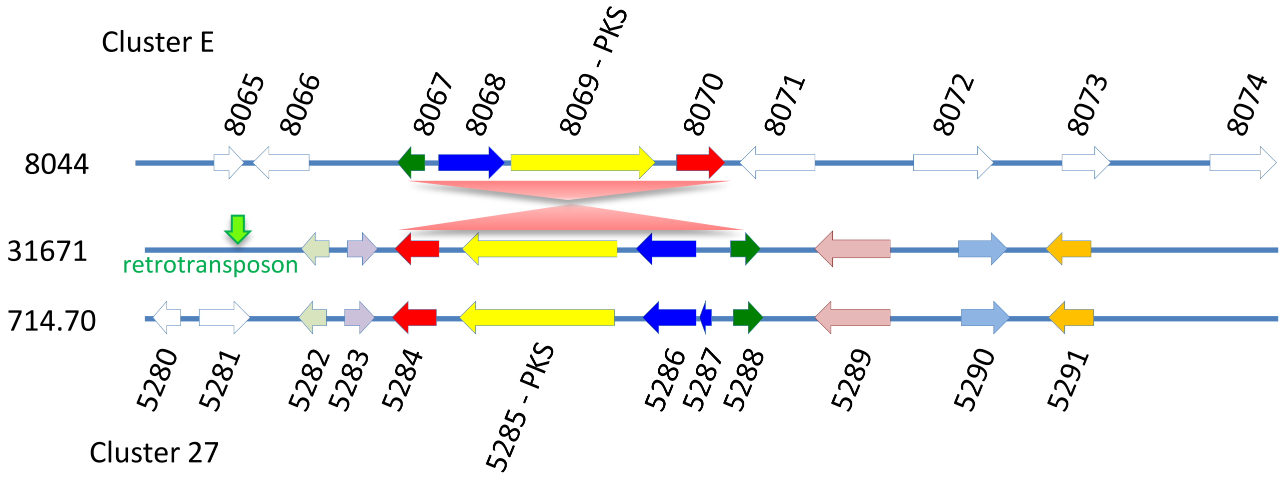

CBS 714.70 Gene No.	BLAST hit
5280	Hypothetical protein
5281	Phosphotransferase family protein [*Pochonia chlamydosporia* 170]
5282	Hypothetical protein
5283	Hypothetical protein
5284	Beta,beta-carotene 15,15'-monoxygenase [*Tolypocladium paradoxum*]
5285	Polyketide synthase [*Cenococcum geophilum* 1.58]
5286	MFS general substrate transporter [*Aspergillus fijiensis* CBS 313.89]*
5287	apha/beta-Hydrolase [Glarea lozoyensis ATCC 20868]*
5288	Related to phospholipid-translocating ATPase [*Fusarium proliferatum* ET1]
5289	ABC transporter
5290	LCCL domain-containing protein [*Colletotrichum orchidophilum*]
5291	Peptidase A1 [*Pochonia chlamydosporia* 170]

* The annotation of MFS transporter was split into two genes in 714.70, but was annotated as a single gene in both 8044 and 31671 and in the previous genome of strain 8044 (Bushley et al., 2013).
