# Supplementary material for: Chromosome rearrangements shape the diversification of secondary metabolism in the cyclosporin producing fungus Tolypocladium inflatum
Source: BMC Genomics. 2019 Feb 7;20:120. doi: 10.1186/s12864-018-5399-x (PMC6367777; doi:10.1186/s12864-018-5399-x)

SFigure 10. Phylogeny of A domains datamined from peptaibiotic NRPSs from *Tolypocladium ophioglossoides* (TOPH), *Trichoderma virens* (TriviGv29;Tex1 and Tex2), and the six strains of *T. inflatum*. Phylogeny was created using maximum implemented in RAxML with best fit amino acid substitution model RT-REV + F and an automatically optimized number of bootstrap replicates. NRPSs from cluster 1 on the left arm of chromosome 1 in *T. inflatum* are color coded dusty pink (13 modular) and bright pink (8 modular). NRPSs from cluster 10 on the right arm of chromosome 1 are color coded light blue (5 modular) or dark blue (11 modular). NRPSs from cluster 40 on chromosome 6 are color coded green (12 modular). The relict A domain from cluster 42 on hromosome 6 is indicated with red bar.

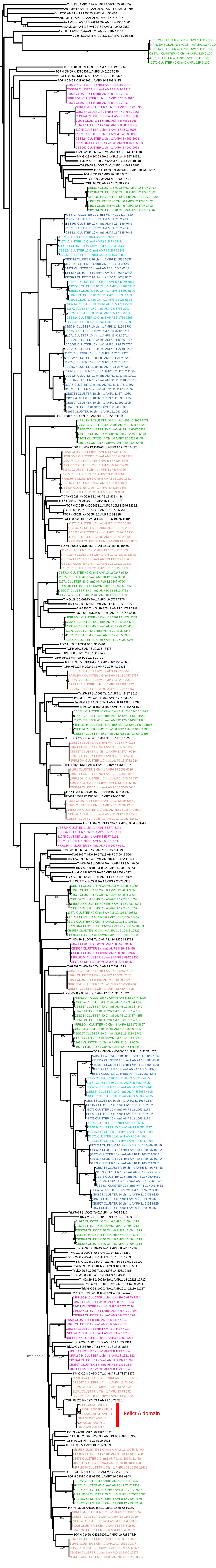

Supplement: Supplementary file 15 — Figure S10. Phylogeny of A-domains of peptaibiotic NRPSs from T. inflatum, T. ophioglossoides, and T. virens. (PDF 25.0 Mb) [file 12864_2018_5399_MOESM15_ESM.pdf]
