# Supplementary material for: Chromosome rearrangements shape the diversification of secondary metabolism in the cyclosporin producing fungus Tolypocladium inflatum
Source: BMC Genomics. 2019 Feb 7;20:120. doi: 10.1186/s12864-018-5399-x (PMC6367777; doi:10.1186/s12864-018-5399-x)

Figure S11 Mauve alignments of peptaibiotic clusters 1, 42, and 10 across all *T. inflatum* strains showing evidence for deletion of large NRPS genes from **A)** clusters 1 in CBS714.70, **B)** loss of the complete cluster 10 in strain 8044, and **C)** cluster 42 in all strains of *T. inflatum* aligned to *T. ophioglossoides* cluster showing missing 16 and 10 modular NRPSs. Red shading shows syntenic blocks within SMBGCs and gray shading shows synteny flanking the clusters.

A) Chromosome 1, Cluster 1

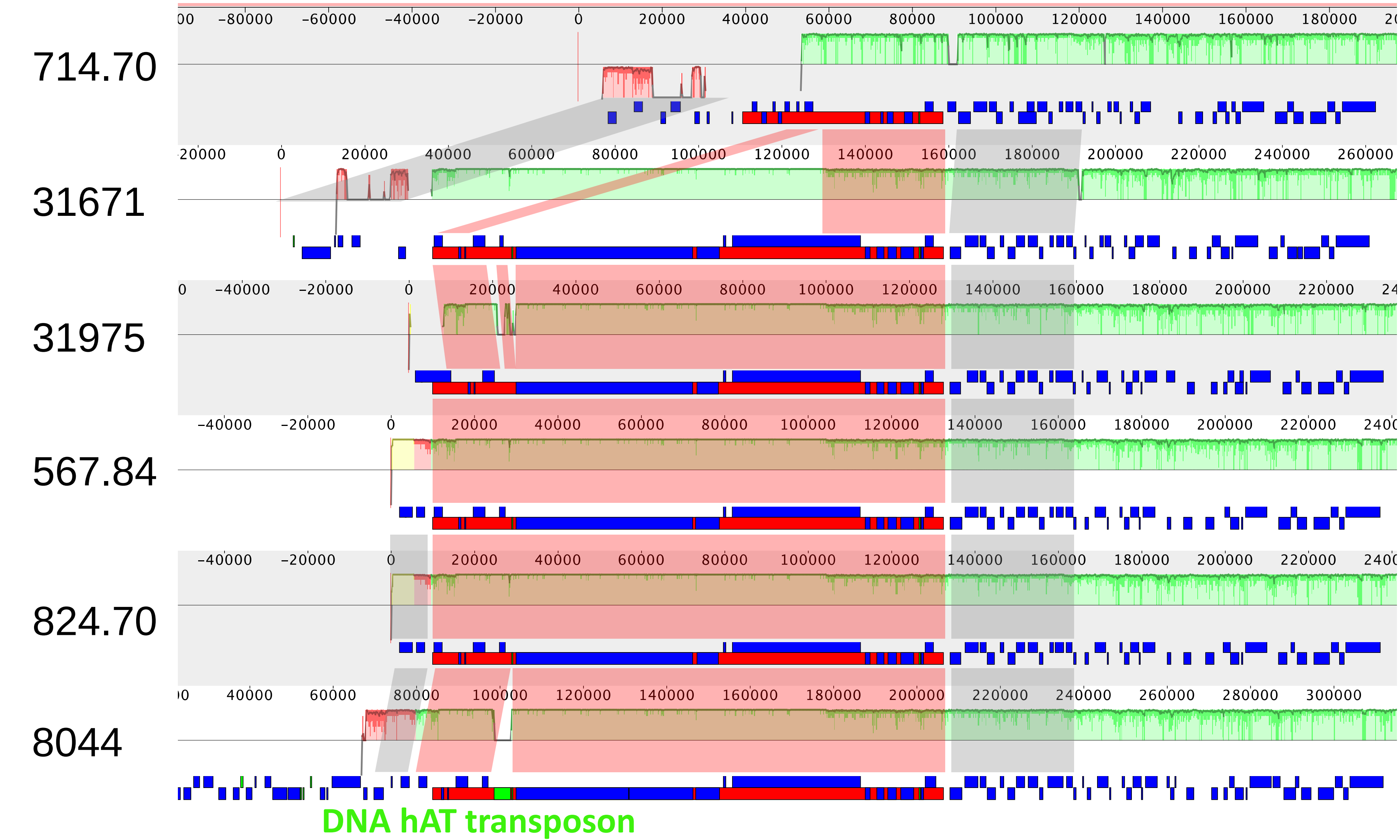

B) Chromosome 1, Cluster 10

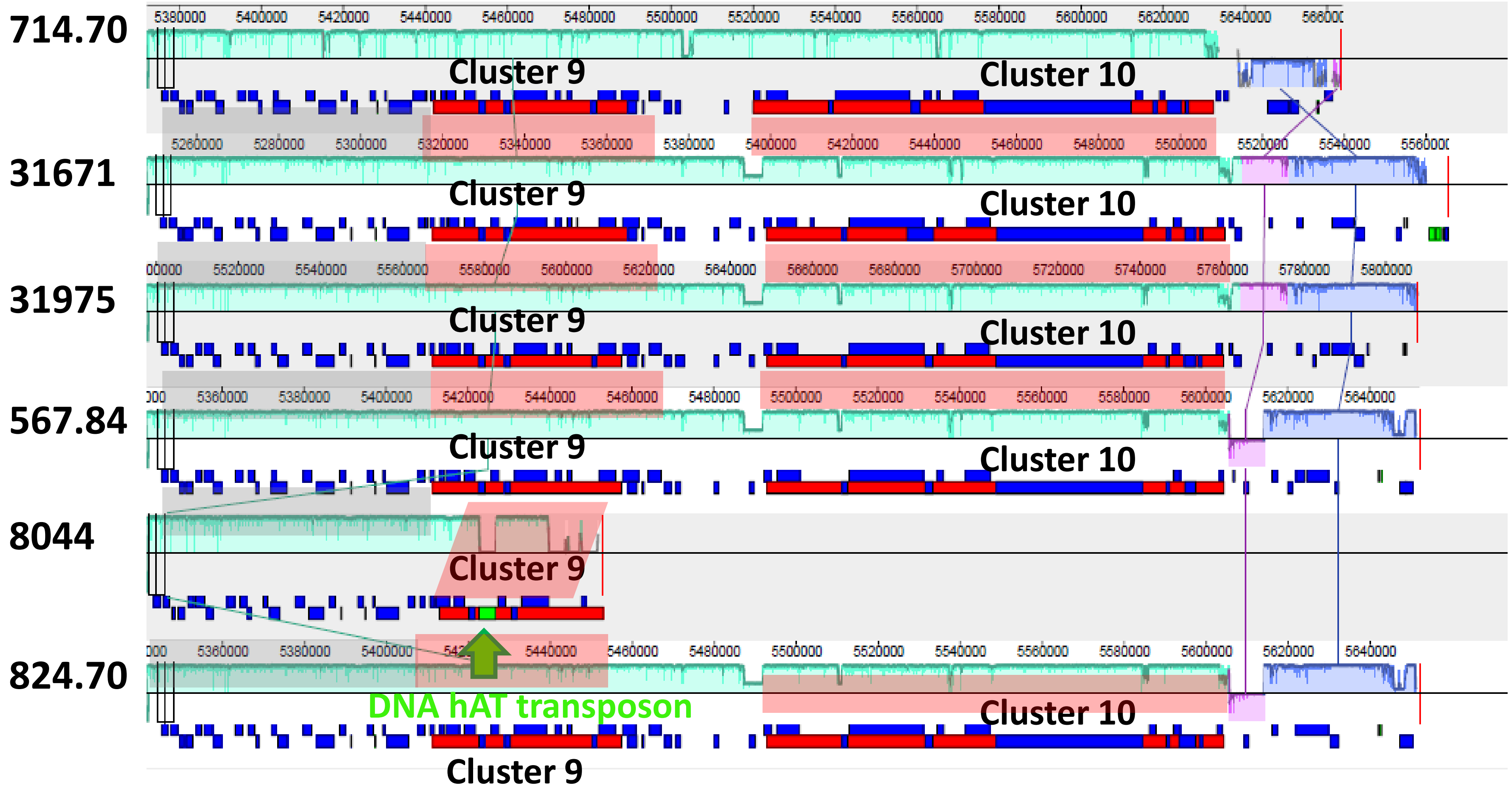

C) Chromosome 6, Cluster 42

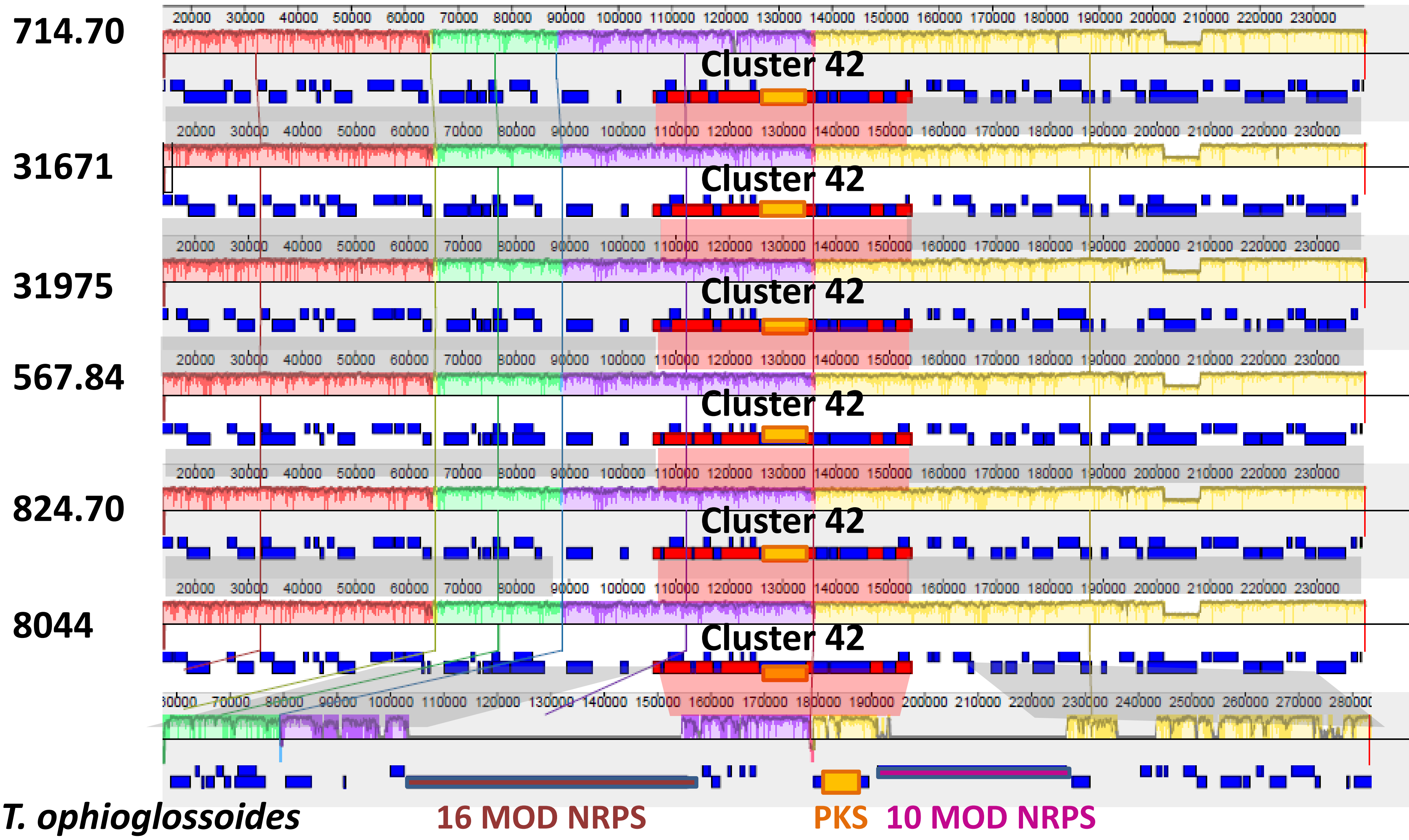

Supplement: Supplementary file 16 — Figure S11. Mauve alignment of peptaibiotic clusters 1, 10 and 42. (PDF 1.25 Mb) [file 12864_2018_5399_MOESM16_ESM.pdf]
