# Supplementary material for: Chromosome rearrangements shape the diversification of secondary metabolism in the cyclosporin producing fungus Tolypocladium inflatum
Source: BMC Genomics. 2019 Feb 7;20:120. doi: 10.1186/s12864-018-5399-x (PMC6367777; doi:10.1186/s12864-018-5399-x)
Supplement: Supplementary file 18 — Figure S12. RAxML trees for homologs of each gene within T. inflatum CBS567.84 and CBS824.70 cluster D datamined from a database of 340 ascomycete fungi. (PDF 61.4 Mb) [file 12864_2018_5399_MOESM18_ESM.pdf]

SFigure 12. Phylogenies of genes from 340 fungal proteomes clustering with individual genes in the aflatoxin-like cluster (cluster D) in *T. inflatum* strain CBS567.84 and CBS824.70. Phylogenies were constructed using maximum likelihood crieteria implemented in RAxML using the best fit model of amino acid substitution using Bayesian Information Criterion and 100 bootstrap replicates. The cluster genes in *T. inflatum* strains are highlighted in red and a key to abbreviations for other fungal taxa is included at the end of the supplement.

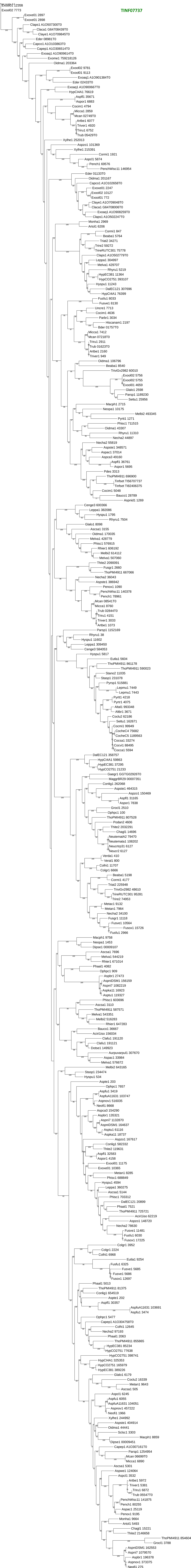

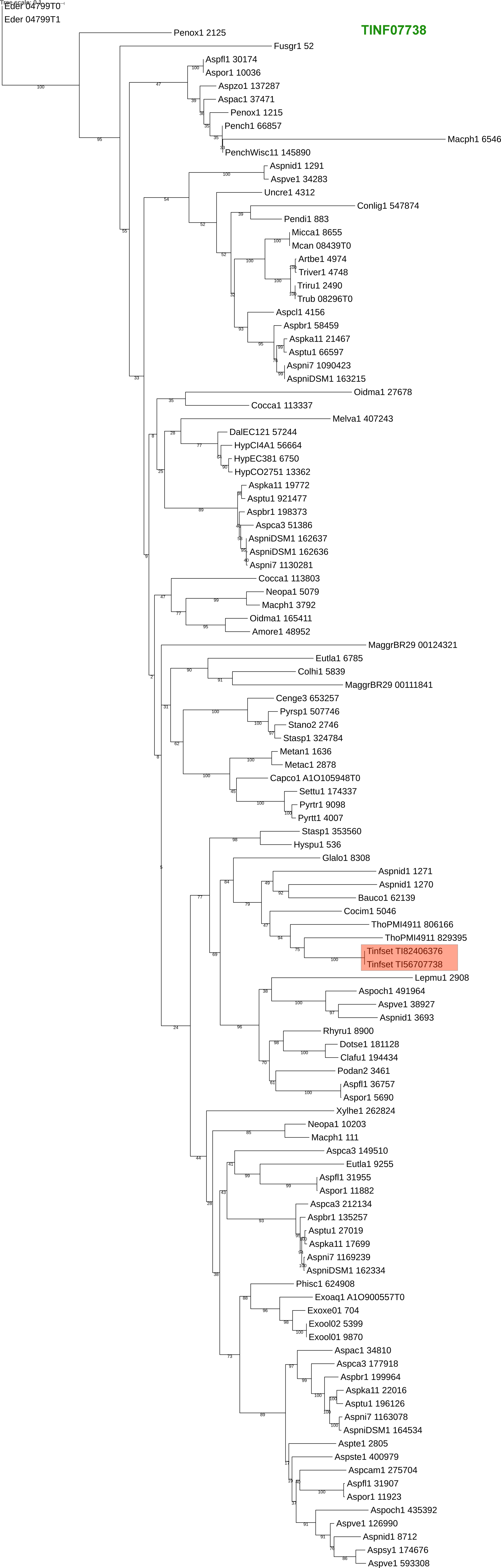

Tree scale: 0.1
TINF07738
Eder 04799T0
Eder 04799T1
Penox1 2125
Fusgr1 52
Aspfl1 30174
Aspor1 10036
Aspzo1 137287
Aspac1 37471
Penox1 1215
Pench1 66857
Macph1 6546
PenchWisc11 145890
Aspnid1 1291
Aspve1 34283
Uncre1 4312
Conlig1 547874
Pendi1 883
Micca1 8655
Mcan 08439T0
Artbe1 4974
Triver1 4748
Triru1 2490
Trub 08296T0
Aspcl1 4156
Aspbr1 58459
Aspka11 21467
Asptu1 66597
Aspni7 1090423
AspniDSM1 163215
Oidma1 27678
Cocca1 113337
Melva1 407243
DalEC121 57244
HypCI4A1 56664
HypEC381 6750
HypCO2751 13362
Aspka11 19772
Asptu1 921477
Aspbr1 198373
Aspca3 51386
AspniDSM1 162637
AspniDSM1 162636
Aspni7 1130281
Cocca1 113803
Neopa1 5079
Macph1 3792
Oidma1 165411
Amore1 48952
MaggrBR29 00124321
Eutla1 6785
Colhi1 5839
MaggrBR29 00111841
Cenge3 653257
Pyrsp1 507746
Stano2 2746
Stasp1 324784
Metan1 1636
Metac1 2878
Capco1 A1O105948T0
Settu1 174337
Pyrtr1 9098
Pyrtt1 4007
Stasp1 353560
Hyspu1 536
Glalo1 8308
Aspnid1 1271
Aspnid1 1270
Bauco1 62139
Cocim1 5046
ThoPMI4911 806166
ThoPMI4911 829395
Tinfset TI82406376
Tinfset TI56707738
Lepmu1 2908
Aspoch1 491964
Aspve1 38927
Aspnid1 3693
Rhyru1 8900
Dotse1 181128
Clafu1 194434
Podan2 3461
Aspfl1 36757
Aspor1 5690
Xylhe1 262824
Neopa1 10203
Macph1 111
Aspca3 149510
Eutla1 9255
Aspfl1 31955
Aspor1 11882
Aspca3 212134
Aspbr1 135257
Asptu1 27019
Aspka11 17699
Aspni7 1169239
AspniDSM1 162334
Phisc1 624908
Exoaq1 A1O900557T0
Exoxe01 704
Exool02 5399
Exool01 9870
Aspac1 34810
Aspca3 177918
Aspbr1 199964
Aspka11 22016
Asptu1 196126
Aspni7 1163078
AspniDSM1 164534
Aspte1 2805
Aspste1 400979
Aspcam1 275704
Aspfl1 31907
Aspor1 11923
Aspoch1 435392
Aspve1 126990
Aspnid1 8712
Aspsy1 174676
Aspve1 593308

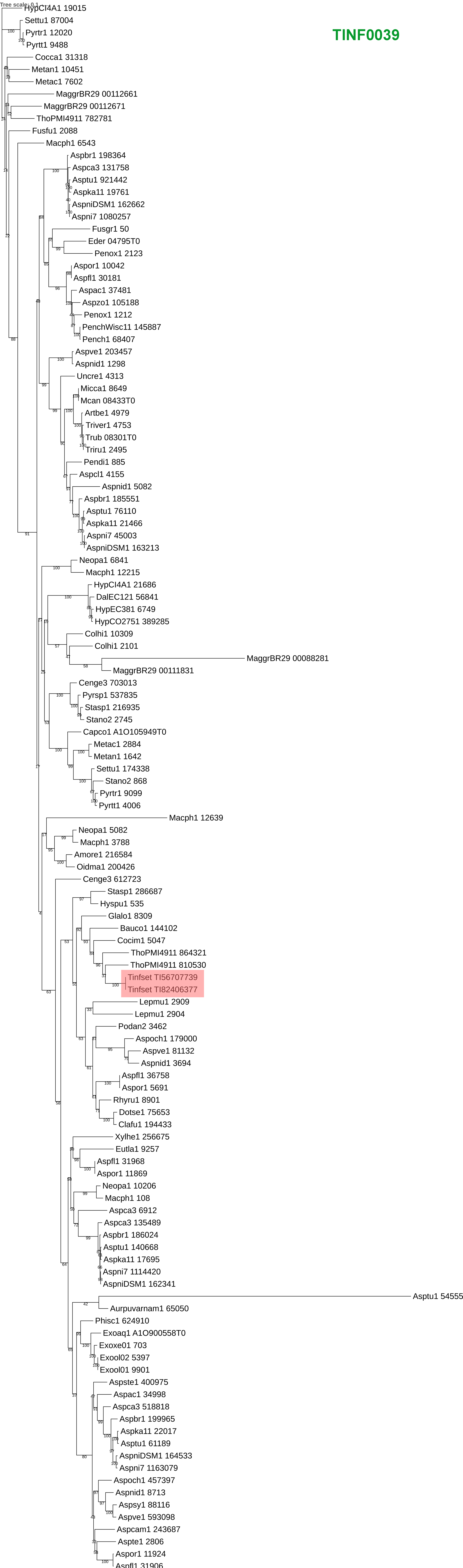
Tree scale: 0.1
TINF0039
HypCI4A1 19015
Settu1 87004
Pyrtr1 12020
Pyrtt1 9488
Cocca1 31318
Metan1 10451
Metac1 7602
MaggrBR29 00112661
MaggrBR29 00112671
ThoPMI4911 782781
Fusfu1 2088
Macph1 6543
Aspbr1 198364
Aspca3 131758
Asptu1 921442
Aspka11 19761
AspniDSM1 162662
Aspni7 1080257
Fusgr1 50
Eder 04795T0
Penox1 2123
Aspor1 10042
Aspfl1 30181
Aspac1 37481
Aspzo1 105188
Penox1 1212
PenchWisc11 145887
Pench1 68407
Aspve1 203457
Aspnid1 1298
Uncre1 4313
Micca1 8649
Mcan 08433T0
Artbe1 4979
Triver1 4753
Trub 08301T0
Triru1 2495
Pendi1 885
Aspcl1 4155
Aspnid1 5082
Aspbr1 185551
Asptu1 76110
Aspka11 21466
Aspni7 45003
AspniDSM1 163213
Neopa1 6841
Macph1 12215
HypCI4A1 21686
DalEC121 56841
HypEC381 6749
HypCO2751 389285
Colhi1 10309
Colhi1 2101
MaggrBR29 00088281
MaggrBR29 00111831
Cenge3 703013
Pyrsp1 537835
Stasp1 216935
Stano2 2745
Capco1 A1O105949T0
Metac1 2884
Metan1 1642
Settu1 174338
Stano2 868
Pyrtr1 9099
Pyrtt1 4006
Macph1 12639
Neopa1 5082
Macph1 3788
Amore1 216584
Oidma1 200426
Cenge3 612723
Stasp1 286687
Hyspu1 535
Glalo1 8309
Bauco1 144102
Cocim1 5047
ThoPMI4911 864321
ThoPMI4911 810530
Tinfset TI56707739
Tinfset TI82406377
Lepmu1 2909
Lepmu1 2904
Podan2 3462
Aspoch1 179000
Aspve1 81132
Aspnid1 3694
Aspfl1 36758
Aspor1 5691
Rhyru1 8901
Dotse1 75653
Clafu1 194433
Xylhe1 256675
Eutla1 9257
Aspfl1 31968
Aspor1 11869
Neopa1 10206
Macph1 108
Aspca3 6912
Aspca3 135489
Aspbr1 186024
Asptu1 140668
Aspka11 17695
Aspni7 1114420
AspniDSM1 162341
Asptu1 54555
Aurpuvarnam1 65050
Phisc1 624910
Exoaq1 A1O900558T0
Exoxe01 703
Exool02 5397
Exool01 9901
Aspste1 400975
Aspac1 34998
Aspca3 518818
Aspbr1 199965
Aspka11 22017
Asptu1 61189
AspniDSM1 164533
Aspni7 1163079
Aspoch1 457397
Aspnid1 8713
Aspsy1 88116
Aspve1 593098
Aspcam1 243687
Aspte1 2806
Aspor1 11924
Aspfl1 31906

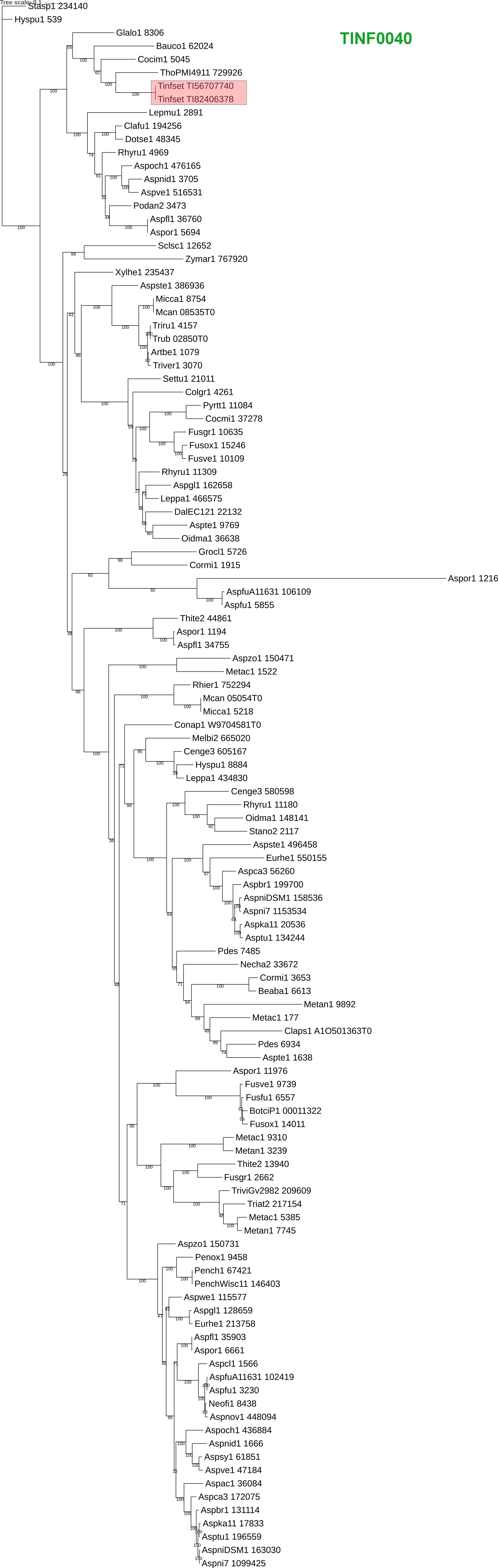
Tree scale: 0.1
TINF0040
Stasp1 234140
Hyspu1 539
Glalo1 8306
Bauco1 62024
Cocim1 5045
ThoPMI4911 729926
Tinfset TI56707740
Tinfset TI82406378
Lepmu1 2891
Clafu1 194256
Dotse1 48345
Rhyru1 4969
Aspoch1 476165
Aspnid1 3705
Aspve1 516531
Podan2 3473
Aspfl1 36760
Aspor1 5694
Sclsc1 12652
Zymar1 767920
Xylhe1 235437
Aspste1 386936
Micca1 8754
Mcan 08535T0
Triru1 4157
Trub 02850T0
Artbe1 1079
Triver1 3070
Settu1 21011
Colgr1 4261
Pyrtt1 11084
Cocmi1 37278
Fusgr1 10635
Fusox1 15246
Fusve1 10109
Rhyru1 11309
Aspgl1 162658
Leppa1 466575
DalEC121 22132
Aspte1 9769
Oidma1 36638
Grocl1 5726
Cormi1 1915
Aspor1 1216
AspfuA11631 106109
Aspfu1 5855
Thite2 44861
Aspor1 1194
Aspfl1 34755
Aspzo1 150471
Metac1 1522
Rhier1 752294
Mcan 05054T0
Micca1 5218
Conap1 W9704581T0
Melbi2 665020
Cenge3 605167
Hyspu1 8884
Leppa1 434830
Cenge3 580598
Rhyru1 11180
Oidma1 148141
Stano2 2117
Aspste1 496458
Eurhe1 550155
Aspca3 56260
Aspbr1 199700
AspniDSM1 158536
Aspni7 1153534
Aspka11 20536
Asptu1 134244
Pdes 7485
Necha2 33672
Cormi1 3653
Beaba1 6613
Metan1 9892
Metac1 177
Claps1 A1O501363T0
Pdes 6934
Aspte1 1638
Aspor1 11976
Fusve1 9739
Fusfu1 6557
BotciP1 00011322
Fusox1 14011
Metac1 9310
Metan1 3239
Thite2 13940
Fusgr1 2662
TriviGv2982 209609
Triat2 217154
Metac1 5385
Metan1 7745
Aspzo1 150731
Penox1 9458
Pench1 67421
PenchWisc11 146403
Aspwe1 115577
Aspgl1 128659
Eurhe1 213758
Aspfl1 35903
Aspor1 6661
Aspcl1 1566
AspfuA11631 102419
Aspfu1 3230
Neofi1 8438
Aspnov1 448094
Aspoch1 436884
Aspnid1 1666
Aspsy1 61851
Aspve1 47184
Aspac1 36084
Aspca3 172075
Aspbr1 131114
Aspka11 17833
Asptu1 196559
AspniDSM1 163030
Aspni7 1099425

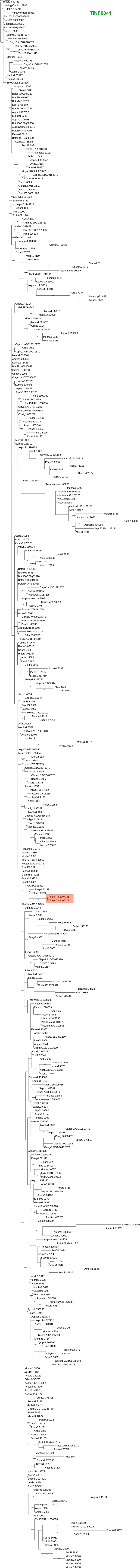
TINF0041

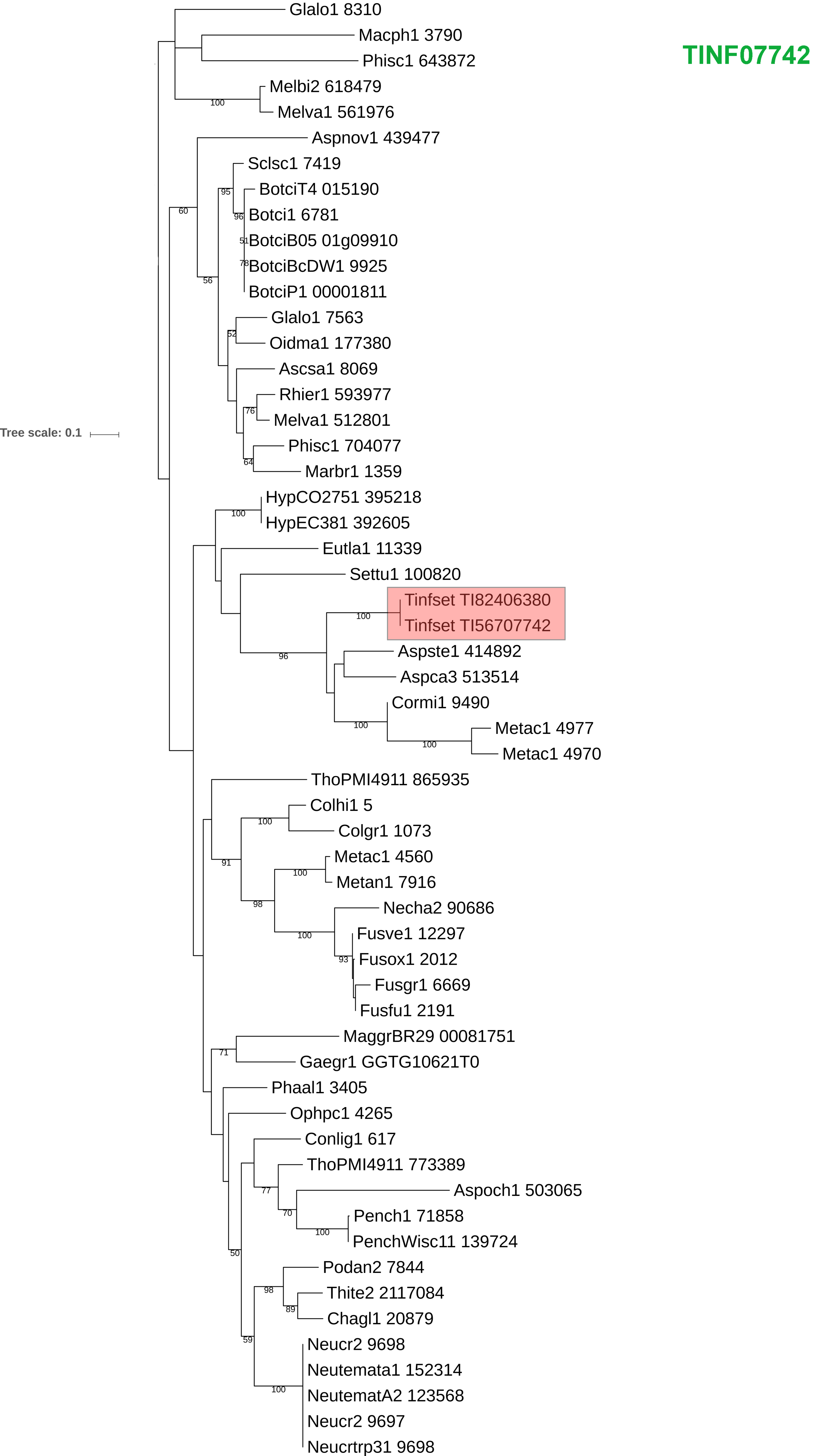
TINF07742
Tree scale: 0.1
Glalo1 8310
Macph1 3790
Phisc1 643872
Melbi2 618479
Melva1 561976
Aspnov1 439477
Sclsc1 7419
BotciT4 015190
Botci1 6781
BotciB05 01g09910
BotciBcDW1 9925
BotciP1 00001811
Glalo1 7563
Oidma1 177380
Ascsa1 8069
Rhier1 593977
Melva1 512801
Phisc1 704077
Marbr1 1359
HypCO2751 395218
HypEC381 392605
Eutla1 11339
Settu1 100820
Tinfset TI82406380
Tinfset TI56707742
Aspste1 414892
Aspca3 513514
Cormi1 9490
Metac1 4977
Metac1 4970
ThoPMI4911 865935
Colhi1 5
Colgr1 1073
Metac1 4560
Metan1 7916
Necha2 90686
Fusve1 12297
Fusox1 2012
Fusgr1 6669
Fusfu1 2191
MaggrBR29 00081751
Gaegr1 GGTG10621T0
Phaal1 3405
Ophpc1 4265
Conlig1 617
ThoPMI4911 773389
Aspoch1 503065
Pench1 71858
PenchWisc11 139724
Podan2 7844
Thite2 2117084
Chagl1 20879
Neucr2 9698
Neutemata1 152314
NeutematA2 123568
Neucr2 9697
Neucrtrp31 9698

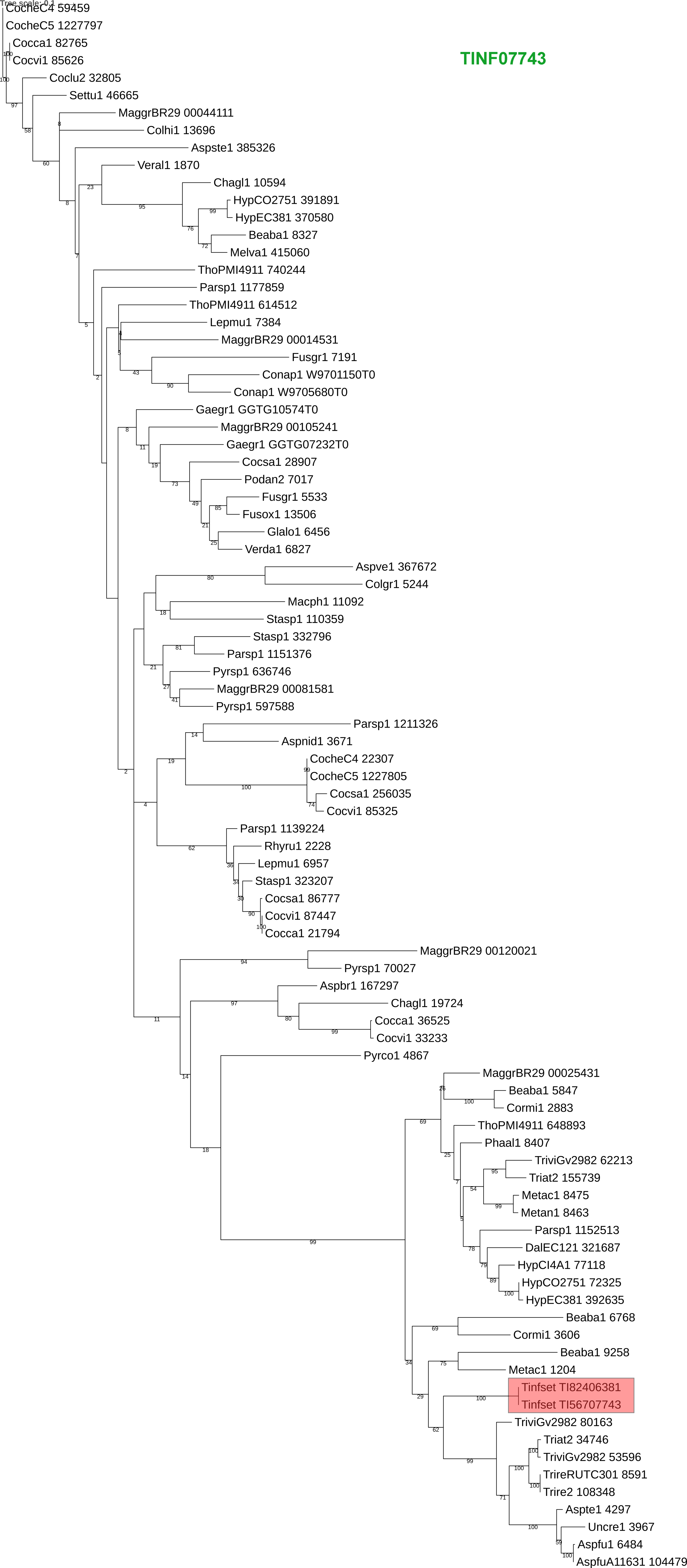
Tree scale: 0.1
TINF07743
CocheC4 59459
CocheC5 1227797
Cocca1 82765
Cocvi1 85626
Coclu2 32805
Settu1 46665
MaggrBR29 00044111
Colhi1 13696
Aspste1 385326
Veral1 1870
Chagl1 10594
HypCO2751 391891
HypEC381 370580
Beaba1 8327
Melva1 415060
ThoPMI4911 740244
Parsp1 1177859
ThoPMI4911 614512
Lepmu1 7384
MaggrBR29 00014531
Fusgr1 7191
Conap1 W9701150T0
Conap1 W9705680T0
Gaegr1 GGTG10574T0
MaggrBR29 00105241
Gaegr1 GGTG07232T0
Cocsa1 28907
Podan2 7017
Fusgr1 5533
Fusox1 13506
Glalo1 6456
Verda1 6827
Aspve1 367672
Colgr1 5244
Macph1 11092
Stasp1 110359
Stasp1 332796
Parsp1 1151376
Pyrsp1 636746
MaggrBR29 00081581
Pyrsp1 597588
Parsp1 1211326
Aspnid1 3671
CocheC4 22307
CocheC5 1227805
Cocsa1 256035
Cocvi1 85325
Parsp1 1139224
Rhyru1 2228
Lepmu1 6957
Stasp1 323207
Cocsa1 86777
Cocvi1 87447
Cocca1 21794
MaggrBR29 00120021
Pyrsp1 70027
Aspbr1 167297
Chagl1 19724
Cocca1 36525
Cocvi1 33233
Pyrco1 4867
MaggrBR29 00025431
Beaba1 5847
Cormi1 2883
ThoPMI4911 648893
Phaal1 8407
TriviGv2982 62213
Triat2 155739
Metac1 8475
Metan1 8463
Parsp1 1152513
DalEC121 321687
HypCI4A1 77118
HypCO2751 72325
HypEC381 392635
Beaba1 6768
Cormi1 3606
Beaba1 9258
Metac1 1204
Tinfset TI82406381
Tinfset TI56707743
TriviGv2982 80163
Triat2 34746
TriviGv2982 53596
TrireRUTC301 8591
Trire2 108348
Aspte1 4297
Uncre1 3967
Aspfu1 6484
AspfuA11631 104479

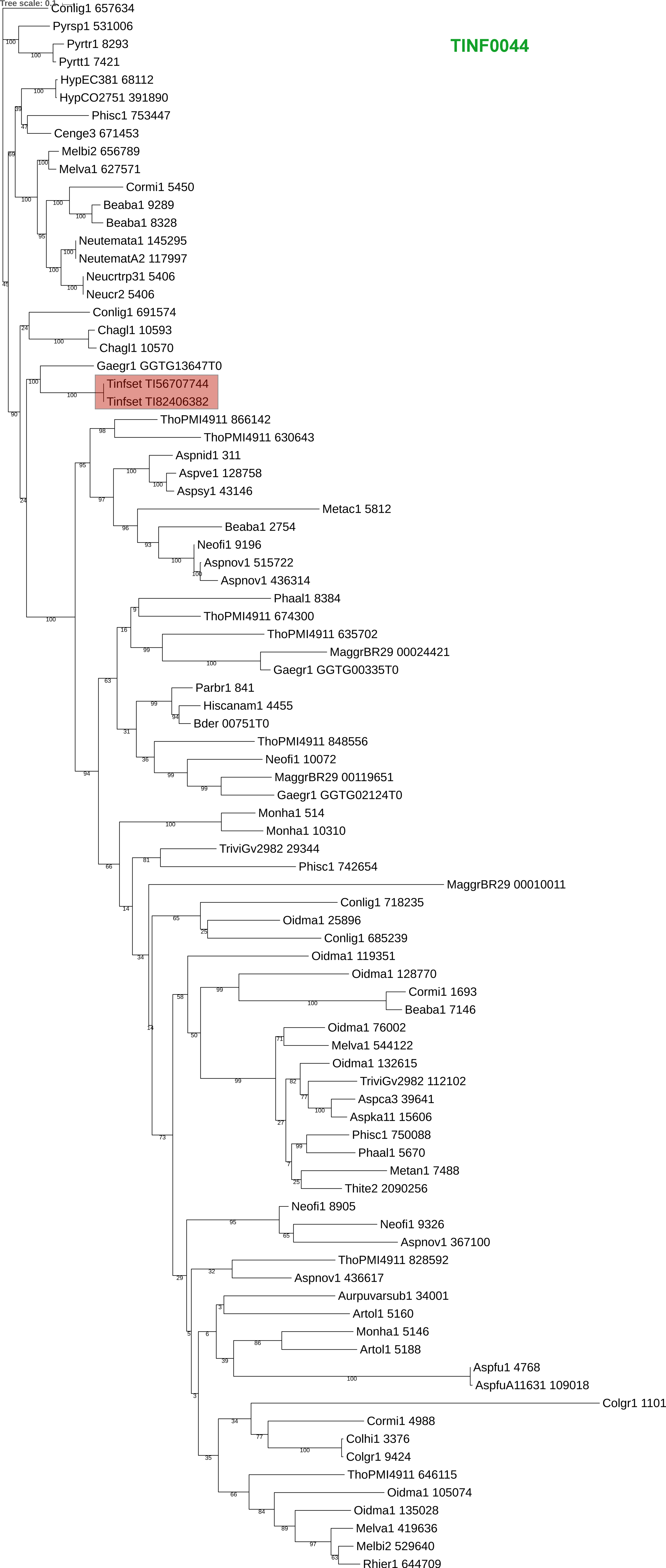
Tree scale: 0.1
TINF0044
Conlig1 657634
Pyrsp1 531006
Pyrtr1 8293
Pyrtt1 7421
HypEC381 68112
HypCO2751 391890
Phisc1 753447
Cenge3 671453
Melbi2 656789
Melva1 627571
Cormi1 5450
Beaba1 9289
Beaba1 8328
Neutemata1 145295
NeutematA2 117997
Neucrtrp31 5406
Neucr2 5406
Conlig1 691574
Chagl1 10593
Chagl1 10570
Gaegr1 GGTG13647T0
Tinfset TI56707744
Tinfset TI82406382
ThoPMI4911 866142
ThoPMI4911 630643
Aspnid1 311
Aspve1 128758
Aspsy1 43146
Metac1 5812
Beaba1 2754
Neofi1 9196
Aspnov1 515722
Aspnov1 436314
Phaal1 8384
ThoPMI4911 674300
ThoPMI4911 635702
MaggrBR29 00024421
Gaegr1 GGTG00335T0
Parbr1 841
Hiscanam1 4455
Bder 00751T0
ThoPMI4911 848556
Neofi1 10072
MaggrBR29 00119651
Gaegr1 GGTG02124T0
Monha1 514
Monha1 10310
TriviGv2982 29344
Phisc1 742654
MaggrBR29 00010011
Conlig1 718235
Oidma1 25896
Conlig1 685239
Oidma1 119351
Oidma1 128770
Cormi1 1693
Beaba1 7146
Oidma1 76002
Melva1 544122
Oidma1 132615
TriviGv2982 112102
Aspca3 39641
Aspka11 15606
Phisc1 750088
Phaal1 5670
Metan1 7488
Thite2 2090256
Neofi1 8905
Neofi1 9326
Aspnov1 367100
ThoPMI4911 828592
Aspnov1 436617
Aurpuvarsub1 34001
Artol1 5160
Monha1 5146
Artol1 5188
Aspfu1 4768
AspfuA11631 109018
Colgr1 1101
Cormi1 4988
Colhi1 3376
Colgr1 9424
ThoPMI4911 646115
Oidma1 105074
Oidma1 135028
Melva1 419636
Melbi2 529640
Rhier1 644709

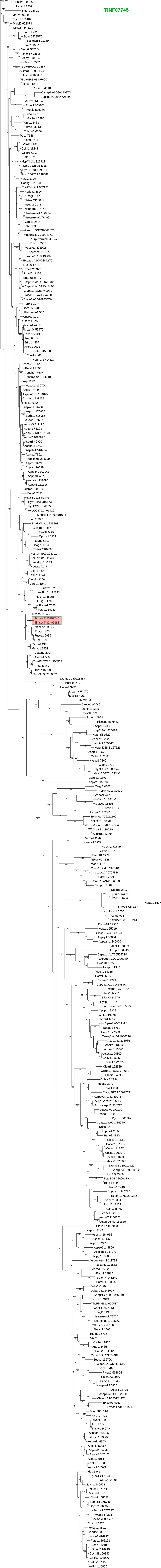
TINF07745

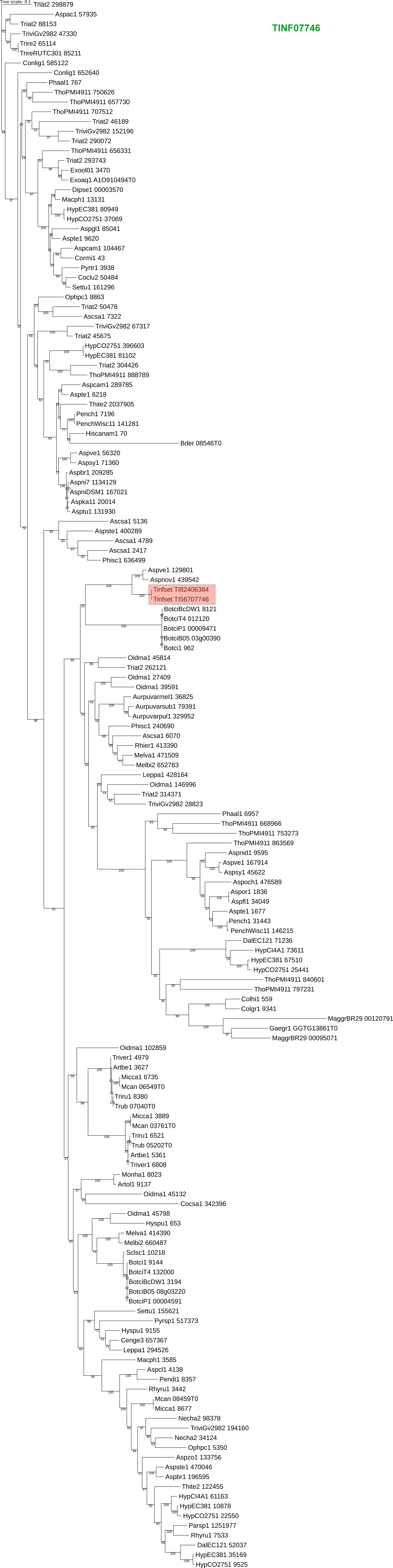
Tree scale: 0.1
TINF07746
Triat2 298879
Aspac1 57935
Triat2 88153
TriviGv2982 47330
Trire2 65114
TrireRUTC301 85211
Conlig1 585122
Conlig1 652640
Phaal1 767
ThoPMI4911 750626
ThoPMI4911 657730
ThoPMI4911 707512
Triat2 46189
TriviGv2982 152196
Triat2 290072
ThoPMI4911 656331
Triat2 293743
Exool01 3470
Exoaq1 A1O910494T0
Dipse1 00003570
Macph1 13131
HypEC381 80949
HypCO2751 37069
Aspgl1 85041
Aspte1 9620
Aspcam1 104467
Cormi1 43
Pyrtr1 3938
Coclu2 50484
Settu1 161296
Ophpc1 8863
Triat2 50478
Ascsa1 7322
TriviGv2982 67317
Triat2 45675
HypCO2751 396603
HypEC381 81102
Triat2 304426
ThoPMI4911 888789
Aspcam1 289785
Aspte1 6218
Thite2 2037905
Pench1 7196
PenchWisc11 141281
Hiscanam1 70
Bder 08546T0
Aspve1 56320
Aspsy1 71360
Aspbr1 209285
Aspni7 1134129
AspniDSM1 167021
Aspka11 20014
Asptu1 131930
Ascsa1 5136
Aspste1 400289
Ascsa1 4789
Ascsa1 2417
Phisc1 636499
Aspve1 129801
Aspnov1 439542
Tinfset TI82406384
Tinfset TI56707746
BotciBcDW1 8121
BotciT4 012120
BotciP1 00009471
BotciB05 03g00390
Botci1 962
Oidma1 45814
Triat2 262121
Oidma1 27409
Oidma1 39591
Aurpuvarmel1 36825
Aurpuvarsub1 79391
Aurpuvarpul1 329952
Phisc1 240690
Ascsa1 6070
Rhier1 413390
Melva1 471509
Melbi2 652783
Leppa1 428164
Oidma1 146996
Triat2 314371
TriviGv2982 28823
Phaal1 6957
ThoPMI4911 668966
ThoPMI4911 753273
ThoPMI4911 863569
Aspnid1 9595
Aspve1 167914
Aspsy1 45622
Aspoch1 476589
Aspor1 1836
Aspfl1 34049
Aspte1 1677
Pench1 31443
PenchWisc11 146215
DalEC121 71236
HypCI4A1 73611
HypEC381 67510
HypCO2751 25441
ThoPMI4911 840601
ThoPMI4911 797231
Colhi1 559
Colgr1 9341
MaggrBR29 00120791
Gaegr1 GGTG13861T0
MaggrBR29 00095071
Oidma1 102859
Triver1 4979
Artbe1 3627
Micca1 6735
Mcan 06549T0
Triru1 8380
Trub 07040T0
Micca1 3889
Mcan 03761T0
Triru1 6521
Trub 05202T0
Artbe1 5361
Triver1 6808
Monha1 8023
Artol1 9137
Oidma1 45132
Cocsa1 342396
Oidma1 45798
Hyspu1 653
Melva1 414390
Melbi2 660487
Sclsc1 10218
Botci1 9144
BotciT4 132000
BotciBcDW1 3194
BotciB05 08g03220
BotciP1 00004591
Settu1 155621
Pyrsp1 517373
Hyspu1 9155
Cenge3 657367
Leppa1 294526
Macph1 3585
Aspcl1 4138
Pendi1 8357
Rhyru1 3442
Mcan 08459T0
Micca1 8677
Necha2 98378
TriviGv2982 194160
Necha2 34124
Ophpc1 5350
Aspzo1 133756
Aspste1 470046
Aspbr1 196595
Thite2 122455
HypCI4A1 61163
HypEC381 10878
HypCO2751 22550
Parsp1 1251977
Rhyru1 7533
DalEC121 52037
HypEC381 35169
HypCO2751 9525

TINF07747

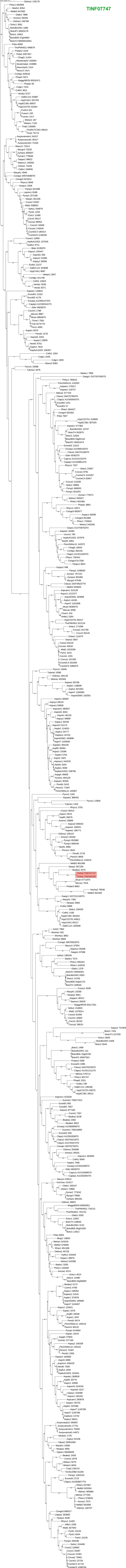

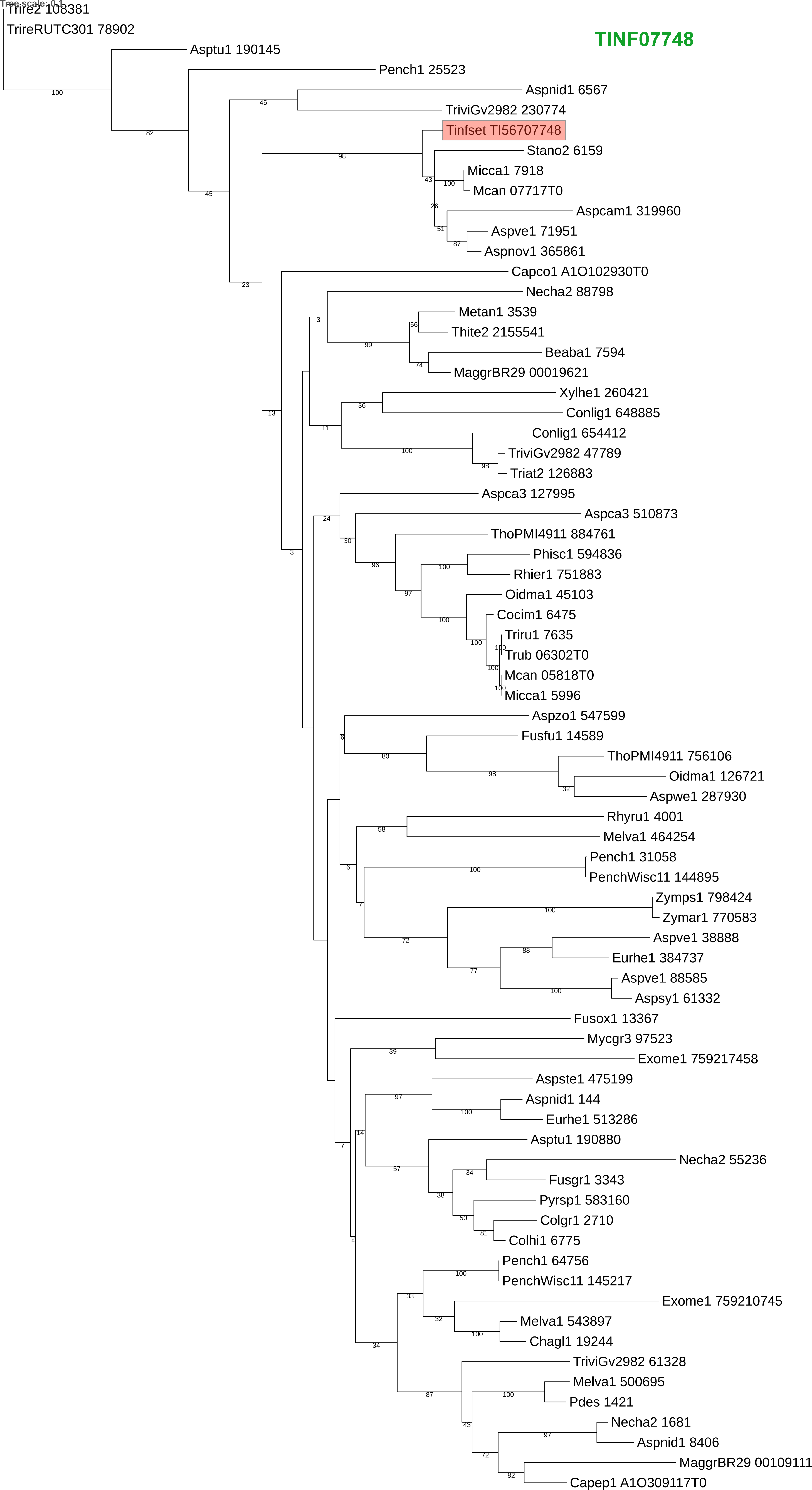
Tree scale: 0.1
TINF07748
Trire2 108381
TrireRUTC301 78902
Asptu1 190145
Pench1 25523
Aspnid1 6567
TriviGv2982 230774
Tinfset TI56707748
Stano2 6159
Micca1 7918
Mcan 07717T0
Aspcam1 319960
Aspve1 71951
Aspnov1 365861
Capco1 A1O102930T0
Necha2 88798
Metan1 3539
Thite2 2155541
Beaba1 7594
MaggrBR29 00019621
Xylhe1 260421
Conlig1 648885
Conlig1 654412
TriviGv2982 47789
Triat2 126883
Aspca3 127995
Aspca3 510873
ThoPMI4911 884761
Phisc1 594836
Rhier1 751883
Oidma1 45103
Cocim1 6475
Triru1 7635
Trub 06302T0
Mcan 05818T0
Micca1 5996
Aspzo1 547599
Fusfu1 14589
ThoPMI4911 756106
Oidma1 126721
Aspwe1 287930
Rhyru1 4001
Melva1 464254
Pench1 31058
PenchWisc11 144895
Zymps1 798424
Zymar1 770583
Aspve1 38888
Eurhe1 384737
Aspve1 88585
Aspsy1 61332
Fusox1 13367
Mycgr3 97523
Exome1 759217458
Aspste1 475199
Aspnid1 144
Eurhe1 513286
Asptu1 190880
Necha2 55236
Fusgr1 3343
Pyrsp1 583160
Colgr1 2710
Colhi1 6775
Pench1 64756
PenchWisc11 145217
Exome1 759210745
Melva1 543897
Chagl1 19244
TriviGv2982 61328
Melva1 500695
Pdes 1421
Necha2 1681
Aspnid1 8406
MaggrBR29 00109111
Capep1 A1O309117T0

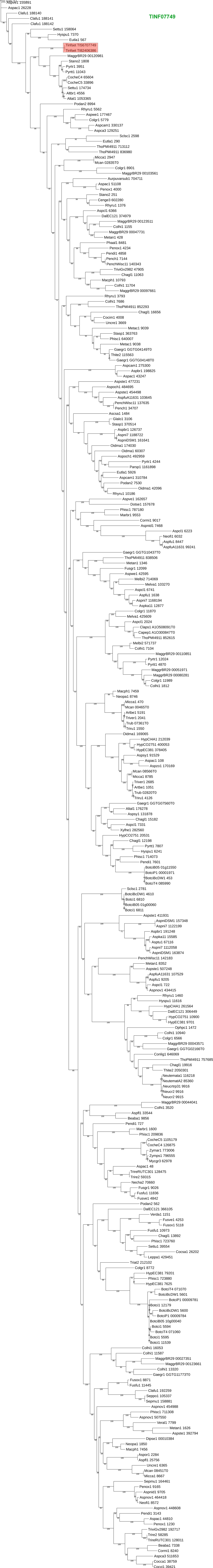
TINF07749
Aspwe1 155891
Aspac1 26228
Clafu1 188140
Clafu1 188141
Clafu1 188142
Settu1 158064
Hyspu1 7370
Eutla1 567
TinfsstT I58707749
TinfsstT I82406386
MaggrBR29 00120981
Stano2 1808
Pyrtr1 3951
Pyrtt1 11043
CocheC4 65604
CocheC5 33896
Settu1 174734
Altbr1 4556
Altal1 1053365
Podan2 8994
Rhyru1 5562
Aspwe1 177467
Colgr1 5779
Aspcam1 330137
Aspca3 129251
Sclsc1 2598
Eutla1 290
ThoPMI4911 713112
ThoPMI4911 836980
Micca1 2947
Mcan 02835T0
Colgr1 8901
MaggrBR29 00103561
Aurpuvarsub1 704711
Aspac1 51108
Penox1 4000
Stano2 251
Cenge3 602280
Rhyru1 1376
Aspcl1 6366
DalEC121 374979
MaggrBR29 00123511
Colhi1 1155
MaggrBR29 00047731
Metan1 428
Phaal1 8481
Penox1 4234
Pendi1 4858
Perch1 7144
PenchWisc11 140343
TrivGv2982 47905
Chagl1 11063
Macph1 10793
Colhi1 11704
MaggrBR29 00097661
Rhyru1 3793
Colhi1 7686
ThoPMI4911 852293
Chagl1 16656
Cocim1 4008
Uncre1 3669
Metac1 9039
Stasp1 363763
Phisc1 640007
Metac1 9038
Gaegr1 GGTG04149T0
Thite2 115563
Gaegr1 GGTG04148T0
Aspcam1 275300
Aspbr1 198825
Aspac1 43247
Aspste1 477231
Aspoch1 484695
Aspste1 454498
AspfuA1163 103645
PenchWisc11 137635
Pench1 34707
Ascsa1 1484
Glalo1 3106
Stasp1 370514
Aspbr1 126737
Aspni7 1188722
AspniDSM1 161641
Oidma1 174030
Oidma1 60307
Aspoch1 492959
Pyrtr1 4244
Parsp1 1161898
Eutla1 5926
Aspcam1 310784
Podan2 7530
Oidma1 42096
Rhyru1 10186
Aspve1 162657
Dotse1 157678
Phisc1 781780
Marbr1 9553
Cormi1 9017
Aspnid1 7468
Aspcl1 6223
Neofi1 6032
Aspfu1 8447
AspfuA1163 99241
Gaegr1 GGTG10437T0
ThoPMI4911 838506
Metan1 1346
Fusgr1 12099
Aspwe1 42595
Melbi2 714069
Melva1 103270
Aspcl1 6741
Aspfu1 1638
Aspni7 1168194
Aspka1 12877
Colgr1 11870
Melva1 425609
Aspcl1 2024
Claps1 A1O50809 1T0
Capep1 A1O300847T0
ThoPMI4911 852615
Melbi2 571737
Colhi1 7104
MaggrBR29 00110851
Pyrtr1 12024
Pyrtt1 4870
MaggrBR29 00051971
MaggrBR29 00080281
Colgr1 11989
Colhi1 1812
Macph1 7459
Neopa1 8746
Micca1 470
Mcan 00465T0
Artbe1 5191
Triver1 2041
Trub 07361T0
Triru1 1550
Oidma1 169065
HypCi4A1 212039
HypCO2751 400053
HypEC381 378405
Aspsy1 91529
Aspac1 108
Aspzo1 170169
Mcan 08566T0
Micca1 8785
Triver1 2685
Artbe1 1051
Trub 02820T0
Triru1 4126
Gaegr1 GGTG07560T0
Altal1 176278
Aspsy1 131878
Chagl1 15182
Aspcl1 7331
Xylhe1 282560
HypCO2751 20531
Colgr1 12198
Pyrtt1 7807
Hyspu1 6241
Phisc1 714073
Pendi1 7601
BotciB05 01g11550
BotciP1 00001971
BotciBcDW1 453
BotciT4 085990
Sclsc1 2781
BotciBcDW1 4610
Botci1 6810
BotciB05 01g00060
Botci1 6811
Aspste1 411931
AspniDSM1 157348
Aspni7 1122199
Aspbr1 191248
Aspka1 15585
Asptu1 67116
Aspni7 1112058
AspniDSM1 163874
PenchWisc11 142183
Metan1 8352
Aspste1 507248
AspfuA1163 107529
Aspfu1 9205
Aspcl1 722
Aspnov1 434415
Rhyru1 1460
Hyspu1 11616
HypCi4A1 261564
DalEC121 306449
HypCO2751 10900
HypEC381 9701
Ophpc1 1472
Colhi1 10940
Colgr1 6566
MaggrBR29 00043571
Gaegr1 GGTG02166T0
Conlig1 640069
ThoPMI4911 757685
Chagl1 19916
Thite2 2050301
Neutematal 116218
NeutematA2 85360
Neucrtrp31 9916
Neucr2 9916
Neucr2 9915
MaggrBR29 00044041
Colhi1 3520
Aspfl1 33544
Beaba1 9856
Pendi1 727
Marbr1 1600
Phisc1 209836
CocheC5 1105179
CocheC4 126875
Zymar1 773006
Zymps1 796555
Mycgr3 62978
Aspac1 48
TrireRUTC301 128475
Trire2 59315
Necha2 70660
Fusgr1 9026
Fusfu1 11836
Fusve1 4842
Podan2 562
DalEC121 366105
Verda1 1151
Fusve1 4253
Fusox1 5118
Fusfu1 10973
Chagl1 13892
Phisc1 723760
Settu1 39554
Cocsa1 26202
Leppa1 429451
Triat2 212102
Colgr1 8772
HypEC381 79201
Phisc1 723880
HypEC381 7625
BotciT4 071070
BotciBcDW1 5601
BotciP1 00009781
Botci1 12179
BotciBcDW1 5600
BotciP1 00009784
BotciB05 10g00040
Botci1 5594
BotciT4 071060
Botci1 5595
Botci1 11539
Colhi1 16053
Colhi1 11587
MaggrBR29 00027351
MaggrBR29 00123661
Colhi1 13320
Gaegr1 GGTG11773T0
Fusox1 8871
Fusfu1 11445
Clafu1 192259
Seppo1 105337
Sepmu1 158881
Aspnov1 454988
Phisc1 711308
Aspnov1 507550
Veral1 7799
Metan1 1626
Aspste1 392794
Dipse1 00010384
Neopa1 1850
Macph1 7456
Aspor1 2284
Aspfl1 25756
Uncre1 6365
Mcan 08451T0
Micca1 8667
Sepmu1 164461
Penox1 9165
Aspnid1 9705
Aspnov1 464418
Neofi1 8572
Aspnov1 448608
Pendi1 3143
Aspac1 44810
Penox1 1230
TrivGv2982 192717
Trire2 58285
TrireRUTC301 128011
Beaba1 7338
Cormi1 8240
Aspca3 511653
Cocca1 38759
Cocca1 39421

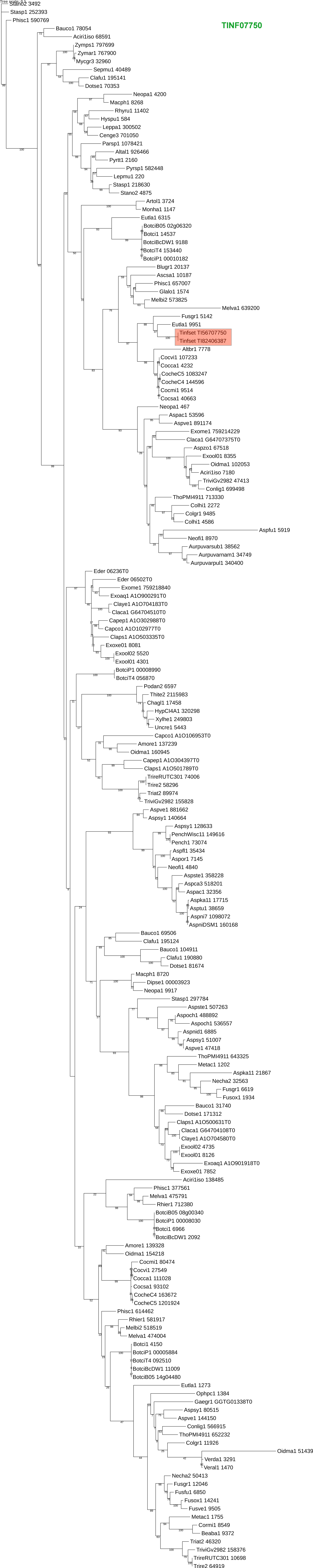
TINF07750
Tinfset TI56707750
Tinfset TI82406387

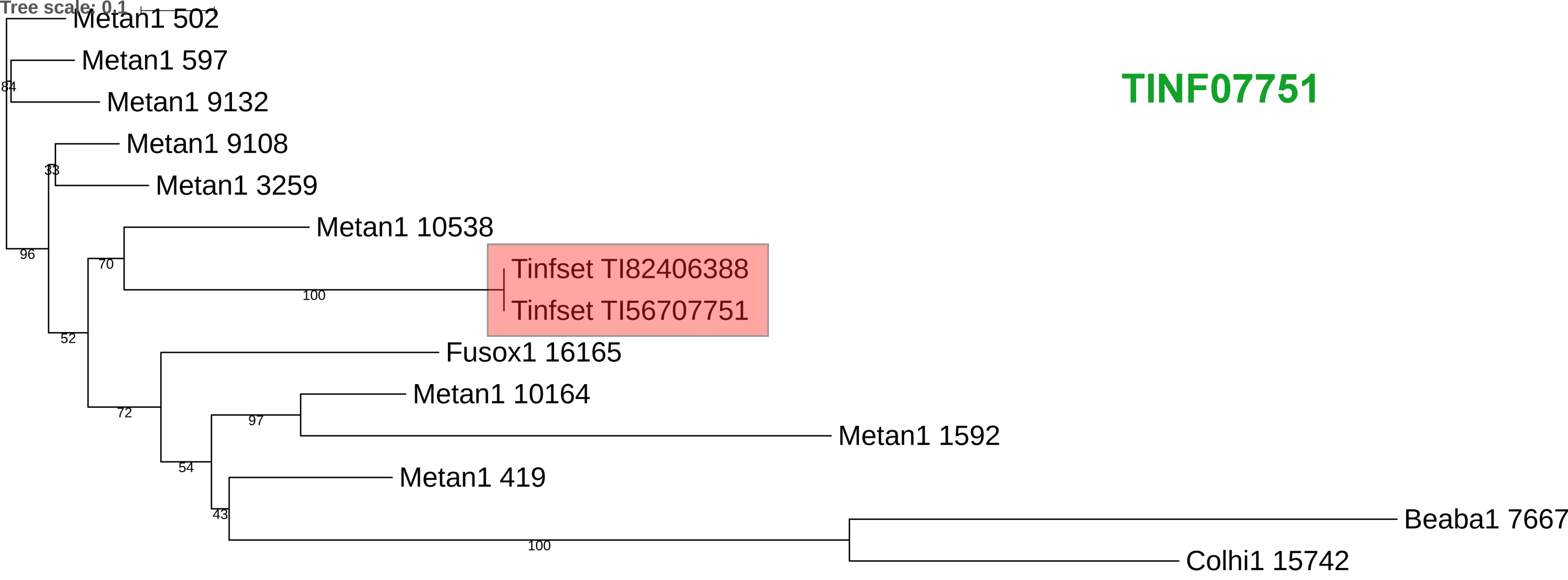
Tree scale: 0.1
TINF07751
Metan1 502
Metan1 597
Metan1 9132
Metan1 9108
Metan1 3259
Metan1 10538
Tinfset TI82406388
Tinfset TI56707751
Fusox1 16165
Metan1 10164
Metan1 1592
Metan1 419
Beaba1 7667
Colhi1 15742
84
33
96
70
100
52
72
97
54
43
100

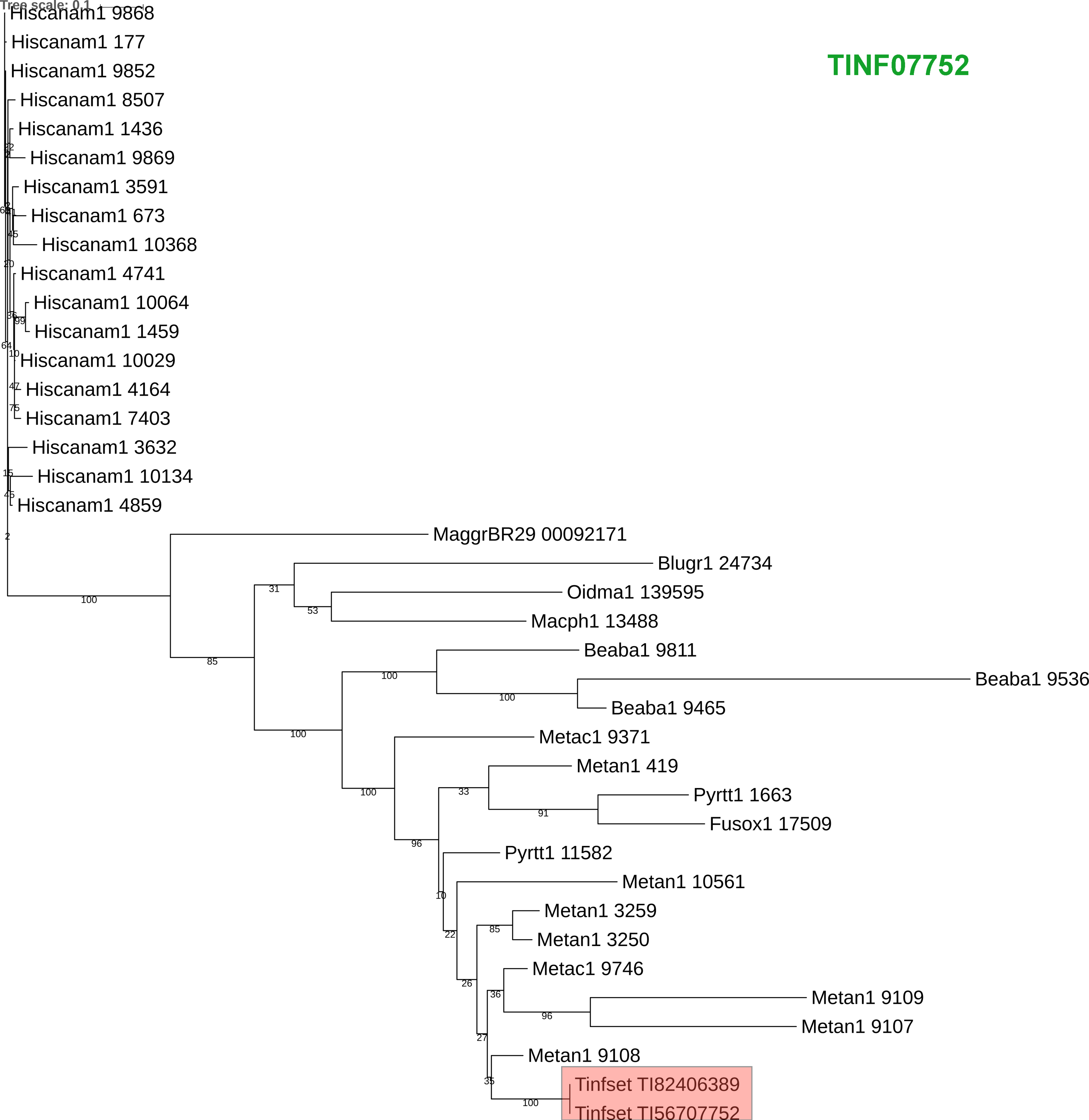
Tree scale: 0.1
TINF07752
Hiscanam1 9868
Hiscanam1 177
Hiscanam1 9852
Hiscanam1 8507
Hiscanam1 1436
Hiscanam1 9869
Hiscanam1 3591
Hiscanam1 673
Hiscanam1 10368
Hiscanam1 4741
Hiscanam1 10064
Hiscanam1 1459
Hiscanam1 10029
Hiscanam1 4164
Hiscanam1 7403
Hiscanam1 3632
Hiscanam1 10134
Hiscanam1 4859
MaggrBR29 00092171
Blugr1 24734
Oidma1 139595
Macph1 13488
Beaba1 9811
Beaba1 9536
Beaba1 9465
Metac1 9371
Metan1 419
Pyrtt1 1663
Fusox1 17509
Pyrtt1 11582
Metan1 10561
Metan1 3259
Metan1 3250
Metac1 9746
Metan1 9109
Metan1 9107
Metan1 9108
Tinfset TI82406389
Tinfset TI56707752

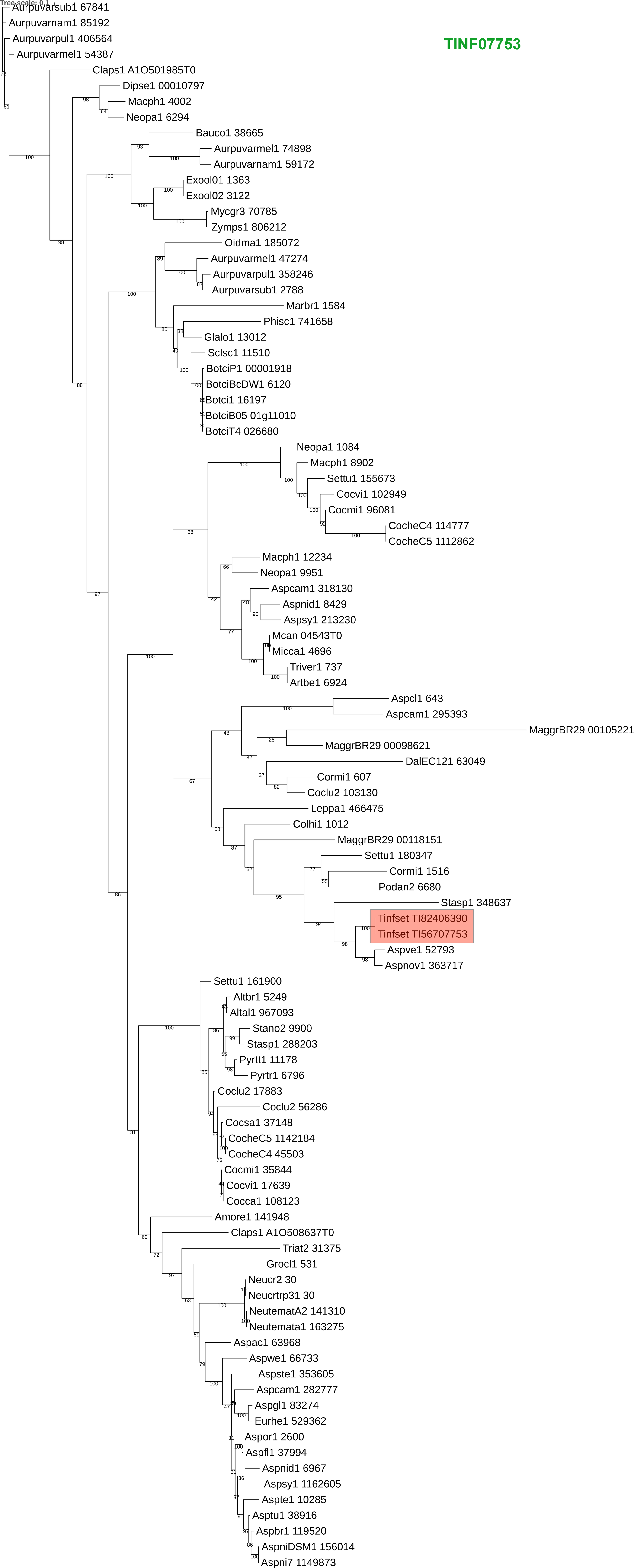
Tree scale: 0.1
TINF07753
Aurpuvarsub1 67841
Aurpuvarnam1 85192
Aurpuvarpul1 406564
Aurpuvarmel1 54387
Claps1 A1O501985T0
Dipse1 00010797
Macph1 4002
Neopa1 6294
Bauco1 38665
Aurpuvarmel1 74898
Aurpuvarnam1 59172
Exool01 1363
Exool02 3122
Mycgr3 70785
Zymps1 806212
Oidma1 185072
Aurpuvarmel1 47274
Aurpuvarpul1 358246
Aurpuvarsub1 2788
Marbr1 1584
Phisc1 741658
Glalo1 13012
Sclsc1 11510
BotciP1 00001918
BotciBcDW1 6120
Botci1 16197
BotciB05 01g11010
BotciT4 026680
Neopa1 1084
Macph1 8902
Settu1 155673
Cocvi1 102949
Cocmi1 96081
CocheC4 114777
CocheC5 1112862
Macph1 12234
Neopa1 9951
Aspcam1 318130
Aspnid1 8429
Aspsy1 213230
Mcan 04543T0
Micca1 4696
Triver1 737
Artbe1 6924
Aspcl1 643
Aspcam1 295393
MaggrBR29 00105221
MaggrBR29 00098621
DalEC121 63049
Cormi1 607
Coclu2 103130
Leppa1 466475
Colhi1 1012
MaggrBR29 00118151
Settu1 180347
Cormi1 1516
Podan2 6680
Stasp1 348637
Tinfset TI82406390
Tinfset TI56707753
Aspve1 52793
Aspnov1 363717
Settu1 161900
Altbr1 5249
Altal1 967093
Stano2 9900
Stasp1 288203
Pyrtt1 11178
Pyrtr1 6796
Coclu2 17883
Coclu2 56286
Cocsa1 37148
CocheC5 1142184
CocheC4 45503
Cocmi1 35844
Cocvi1 17639
Cocca1 108123
Amore1 141948
Claps1 A1O508637T0
Triat2 31375
Grocl1 531
Neucr2 30
Neucrtrp31 30
NeutematA2 141310
Neutemata1 163275
Aspac1 63968
Aspwe1 66733
Aspste1 353605
Aspcam1 282777
Aspgl1 83274
Eurhe1 529362
Aspor1 2600
Aspfl1 37994
Aspnid1 6967
Aspsy1 1162605
Aspte1 10285
Asptu1 38916
Aspbr1 119520
AspniDSM1 156014
Aspni7 1149873

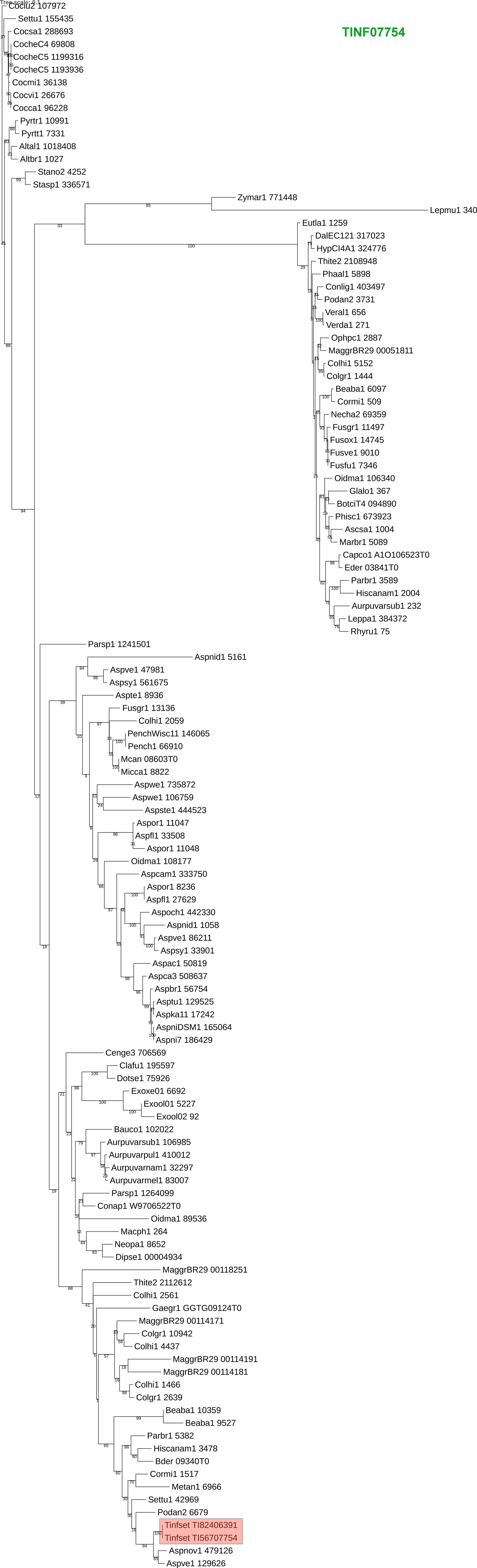
TINF07754
Coclu2 107972
Settu1 155435
Cocsa1 288693
CocheC4 69808
CocheC5 1199316
CocheC5 1193936
Cocmi1 36138
Cocvi1 26676
Cocca1 96228
Pyrtr1 10991
Pyrtt1 7331
Altal1 1018408
Altbr1 1027
Stano2 4252
Stasp1 336571
Zymar1 771448
Lepmu1 340
Eutla1 1259
DalEC121 317023
HypCI4A1 324776
Thite2 2108948
Phaal1 5898
Conlig1 403497
Podan2 3731
Veral1 656
Verda1 271
Ophpc1 2887
MaggrBR29 00051811
Colhi1 5152
Colgr1 1444
Beaba1 6097
Cormi1 509
Necha2 69359
Fusgr1 11497
Fusox1 14745
Fusve1 9010
Fusfu1 7346
Oidma1 106340
Glalo1 367
BotciT4 094890
Phisc1 673923
Ascsa1 1004
Marbr1 5089
Capco1 A1O106523T0
Eder 03841T0
Parbr1 3589
Hiscanam1 2004
Aurpuvarsub1 232
Leppa1 384372
Rhyru1 75
Parsp1 1241501
Aspnid1 5161
Aspve1 47981
Aspsy1 561675
Aspte1 8936
Fusgr1 13136
Colhi1 2059
PenchWisc11 146065
Pench1 66910
Mcan 08603T0
Micca1 8822
Aspwe1 735872
Aspwe1 106759
Aspste1 444523
Aspor1 11047
Aspfl1 33508
Aspor1 11048
Oidma1 108177
Aspcam1 333750
Aspor1 8236
Aspfl1 27629
Aspoch1 442330
Aspnid1 1058
Aspve1 86211
Aspsy1 33901
Aspac1 50819
Aspca3 508637
Aspbr1 56754
Asptu1 129525
Aspka11 17242
AspniDSM1 165064
Aspni7 186429
Cenge3 706569
Clafu1 195597
Dotse1 75926
Exoxe01 6692
Exool01 5227
Exool02 92
Bauco1 102022
Aurpuvarsub1 106985
Aurpuvarpul1 410012
Aurpuvarnam1 32297
Aurpuvarmel1 83007
Parsp1 1264099
Conap1 W9706522T0
Oidma1 89536
Macph1 264
Neopa1 8652
Dipse1 00004934
MaggrBR29 00118251
Thite2 2112612
Colhi1 2561
Gaegr1 GGTG09124T0
MaggrBR29 00114171
Colgr1 10942
Colhi1 4437
MaggrBR29 00114191
MaggrBR29 00114181
Colhi1 1466
Colgr1 2639
Beaba1 10359
Beaba1 9527
Parbr1 5382
Hiscanam1 3478
Bder 09340T0
Cormi1 1517
Metan1 6966
Settu1 42969
Podan2 6679
Tinfset TI82406391
Tinfset TI56707754
Aspnov1 479126
Aspve1 129626

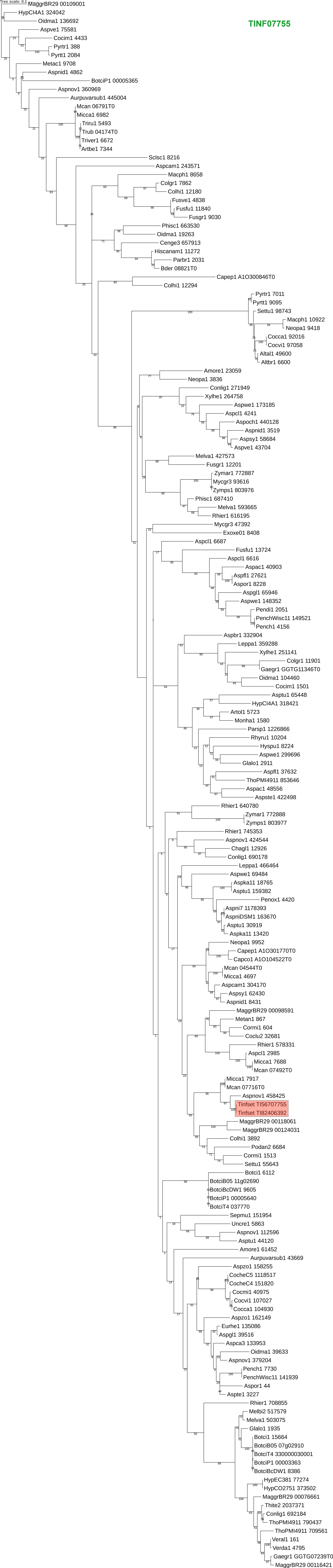
Tree scale: 0.1
TINF07755
MaggrBR29 00109001
HypCI4A1 324042
Oidma1 136692
Aspve1 75581
Cocim1 4433
Pyrtr1 388
Pyrtt1 2084
Metac1 9708
Aspnid1 4862
BotciP1 00005365
Aspnov1 360969
Aurpuvarsub1 445004
Mcan 06791T0
Micca1 6982
Triru1 5493
Trub 04174T0
Triver1 6672
Artbe1 7344
Sclsc1 8216
Aspcam1 243571
Macph1 8658
Colgr1 7862
Colhi1 12180
Fusve1 4838
Fusfu1 11840
Fusgr1 9030
Phisc1 663530
Oidma1 19263
Cenge3 657913
Hiscanam1 11272
Parbr1 2031
Bder 08821T0
Capep1 A1O300846T0
Colhi1 12294
Pyrtr1 7011
Pyrtt1 9095
Settu1 98743
Macph1 10922
Neopa1 9418
Cocca1 92016
Cocvi1 97058
Altal1 49600
Altbr1 6600
Amore1 23059
Neopa1 3836
Conlig1 271949
Xylhe1 264758
Aspwe1 173185
Aspcl1 4241
Aspoch1 440128
Aspnid1 3519
Aspsy1 58684
Aspve1 43704
Melva1 427573
Fusgr1 12201
Zymar1 772887
Mycgr3 93616
Zymps1 803976
Phisc1 687410
Melva1 593665
Rhier1 616195
Mycgr3 47392
Exoxe01 8408
Aspcl1 6687
Fusfu1 13724
Aspcl1 6616
Aspac1 40903
Aspfl1 27621
Aspor1 8228
Aspgl1 65946
Aspwe1 148352
Pendi1 2051
PenchWisc11 149521
Pench1 4156
Aspbr1 332904
Leppa1 359288
Xylhe1 251141
Colgr1 11901
Gaegr1 GGTG11346T0
Oidma1 104460
Cocim1 1501
Asptu1 65448
HypCI4A1 318421
Artol1 5723
Monha1 1580
Parsp1 1226866
Rhyru1 10204
Hyspu1 8224
Aspwe1 299696
Glalo1 2911
Aspfl1 37632
ThoPMI4911 853646
Aspac1 48556
Aspste1 422498
Rhier1 640780
Zymar1 772888
Zymps1 803977
Rhier1 745353
Aspnov1 424544
Chagl1 12926
Conlig1 690178
Leppa1 466464
Aspwe1 69484
Aspka11 18765
Asptu1 159382
Penox1 4420
Aspni7 1178393
AspniDSM1 163670
Asptu1 30919
Aspka11 13420
Neopa1 9952
Capep1 A1O301770T0
Capco1 A1O104522T0
Mcan 04544T0
Micca1 4697
Aspcam1 304170
Aspsy1 62430
Aspnid1 8431
MaggrBR29 00098591
Metan1 867
Cormi1 604
Coclu2 32681
Rhier1 578331
Aspcl1 2985
Micca1 7688
Mcan 07492T0
Micca1 7917
Mcan 07716T0
Aspnov1 458425
Tinfset TI56707755
Tinfset TI82406392
MaggrBR29 00118061
MaggrBR29 00124031
Colhi1 3892
Podan2 6684
Cormi1 1513
Settu1 55643
Botci1 6112
BotciB05 11g02690
BotciBcDW1 9605
BotciP1 00005640
BotciT4 037770
Sepmu1 151954
Uncre1 5863
Aspnov1 112596
Asptu1 44120
Amore1 61452
Aurpuvarsub1 43669
Aspzo1 158255
CocheC5 1118517
CocheC4 151820
Cocmi1 40975
Cocvi1 107027
Cocca1 104930
Aspzo1 162149
Eurhe1 135086
Aspgl1 39516
Aspca3 133953
Oidma1 39633
Aspnov1 379204
Pench1 7730
PenchWisc11 141939
Aspor1 44
Aspte1 3227
Rhier1 708855
Melbi2 517579
Melva1 503075
Glalo1 1935
Botci1 15664
BotciB05 07g02910
BotciT4 330000030001
BotciP1 00003363
BotciBcDW1 8386
HypEC381 77274
HypCO2751 373502
MaggrBR29 00076661
Thite2 2037371
Conlig1 692184
ThoPMI4911 790437
ThoPMI4911 709561
Veral1 161
Verda1 4795
Gaegr1 GGTG07239T0
MaggrBR29 00116421

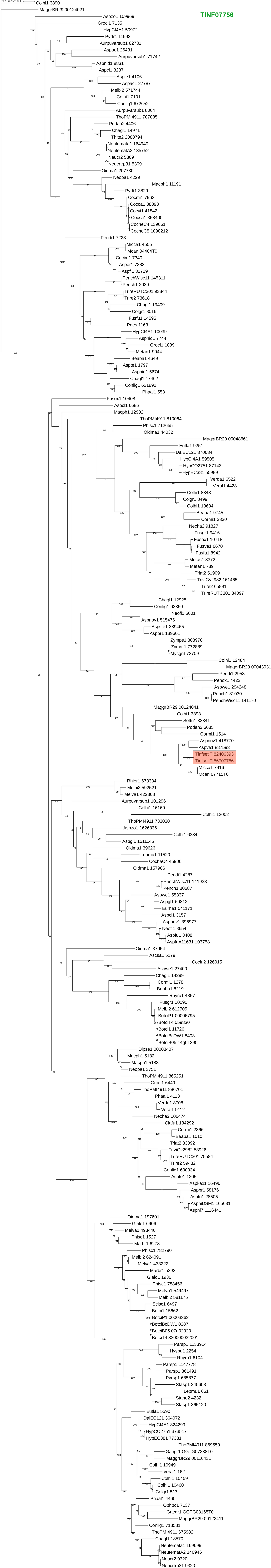
TINF07756
Tree scale: 0.1
Colhi1 3890
MaggrBR29 00124021
Aspzo1 109969
Grocl1 7135
HypCI4A1 50972
Pyrtr1 11992
Aurpuvarsub1 62731
Aspac1 26431
Aurpuvarsub1 71742
Aspnid1 8831
Aspcl1 3237
Aspte1 4106
Aspac1 27787
Melbi2 571744
Colhi1 7101
Conlig1 672652
Aurpuvarsub1 8064
ThoPMI4911 707885
Podan2 4406
Chagl1 14971
Thite2 2088794
Neutemata1 164940
NeutemataA2 135752
Neucr2 5309
Neucrtrp31 5309
Oidma1 207730
Neopa1 4229
Macph1 11191
Pyrtt1 3829
Cocmi1 7963
Cocca1 38898
Cocvi1 41842
Cocsa1 358400
CocheC4 139661
CocheC5 1098212
Pendi1 7223
Micca1 4555
Mcan 04404T0
Cocim1 7340
Aspor1 7282
Aspfl1 31729
PenchWisc11 145311
Pench1 2039
TrireRUTC301 93844
Trire2 73618
Chagl1 19409
Colgr1 8016
Fusfu1 14595
Pdes 1163
HypCI4A1 10039
Aspnid1 7744
Grocl1 1839
Metan1 9944
Beaba1 4649
Aspte1 1797
Aspnid1 5674
Chagl1 17462
Conlig1 621892
Phaal1 553
Fusox1 10408
Aspcl1 6686
Macph1 12982
ThoPMI4911 810064
Phisc1 712655
Oidma1 44032
MaggrBR29 00048661
Eutla1 9251
DalEC121 370634
HypCI4A1 59505
HypCO2751 87143
HypEC381 55989
Verda1 6522
Veral1 4428
Colhi1 8343
Colgr1 8499
Colhi1 13634
Beaba1 9745
Cormi1 3330
Necha2 91827
Fusgr1 9416
Fusox1 10718
Fusve1 6670
Fusfu1 8942
Metac1 8372
Metan1 789
Triat2 51909
TriviGv2982 161465
Trire2 65891
TrireRUTC301 84097
Chagl1 12925
Conlig1 63350
Neofi1 5001
Aspnov1 515476
Aspste1 389465
Aspbr1 139601
Zymps1 803978
Zymar1 772889
Mycgr3 72709
Colhi1 12484
MaggrBR29 00043931
Pendi1 2953
Penox1 4422
Aspwe1 294248
Pench1 81030
PenchWisc11 141170
MaggrBR29 00124041
Colhi1 3893
Settu1 33341
Podan2 6685
Cormi1 1514
Aspnov1 418770
Aspve1 887593
Tinfset TI82406393
Tinfset TI56707756
Micca1 7916
Mcan 07715T0
Rhier1 673334
Melbi2 592521
Melva1 422368
Aurpuvarsub1 101296
Colhi1 16160
Colhi1 12002
ThoPMI4911 733030
Aspzo1 1626836
Colhi1 6334
Aspgl1 1511145
Oidma1 39626
Lepmu1 11520
CocheC4 45906
Oidma1 157986
Pendi1 4287
PenchWisc11 141938
Pench1 80687
Aspwe1 55337
Aspgl1 69812
Eurhe1 541171
Aspcl1 3157
Aspnov1 396977
Neofi1 8654
Aspfu1 3408
AspfuA11631 103758
Oidma1 37954
Ascsa1 5179
Coclu2 126015
Aspwe1 27400
Chagl1 14299
Cormi1 1278
Beaba1 8219
Rhyru1 4857
Fusgr1 10090
Melbi2 612705
BotciP1 00006795
BotciT4 059830
Botci1 11726
BotciBcDW1 8403
BotciB05 14g01290
Dipse1 00008407
Macph1 5182
Macph1 5183
Neopa1 3751
ThoPMI4911 865251
Grocl1 6449
ThoPMI4911 886701
Phaal1 4113
Verda1 8708
Veral1 9112
Necha2 106474
Clafu1 184292
Cormi1 2366
Beaba1 1010
Triat2 33092
TriviGv2982 53926
TrireRUTC301 75584
Trire2 59482
Conlig1 690934
Aspte1 1205
Aspka11 16496
Aspbr1 58176
Asptu1 28505
AspniDSM1 165631
Aspni7 1116441
Oidma1 197601
Glalo1 6906
Melva1 498440
Phisc1 1527
Marbr1 6278
Phisc1 782790
Melbi2 624091
Melva1 433222
Marbr1 5392
Phisc1 788456
Glalo1 1936
Melva1 549497
Melbi2 581175
Sclsc1 6497
Botci1 15662
BotciP1 00003362
BotciBcDW1 8387
BotciB05 07g02920
BotciT4 330000032001
Parsp1 1133914
Hyspu1 2254
Rhyru1 6104
Parsp1 1147778
Parsp1 861491
Pyrsp1 685877
Stasp1 245653
Lepmu1 661
Stano2 4232
Stasp1 365120
Eutla1 5590
DalEC121 364072
HypCI4A1 324299
HypCO2751 373517
HypEC381 77331
ThoPMI4911 869559
Gaegr1 GGTG07238T0
MaggrBR29 00116431
Colhi1 10949
Veral1 162
Colhi1 10459
Colhi1 10460
Colgr1 517
Phaal1 4460
Ophpc1 7137
Gaegr1 GGTG03165T0
MaggrBR29 00122411
Conlig1 718581
ThoPMI4911 675982
Chagl1 18570
Neutemata1 169699
NeutemataA2 140946
Neucr2 9320
Neucrtrp31 9320

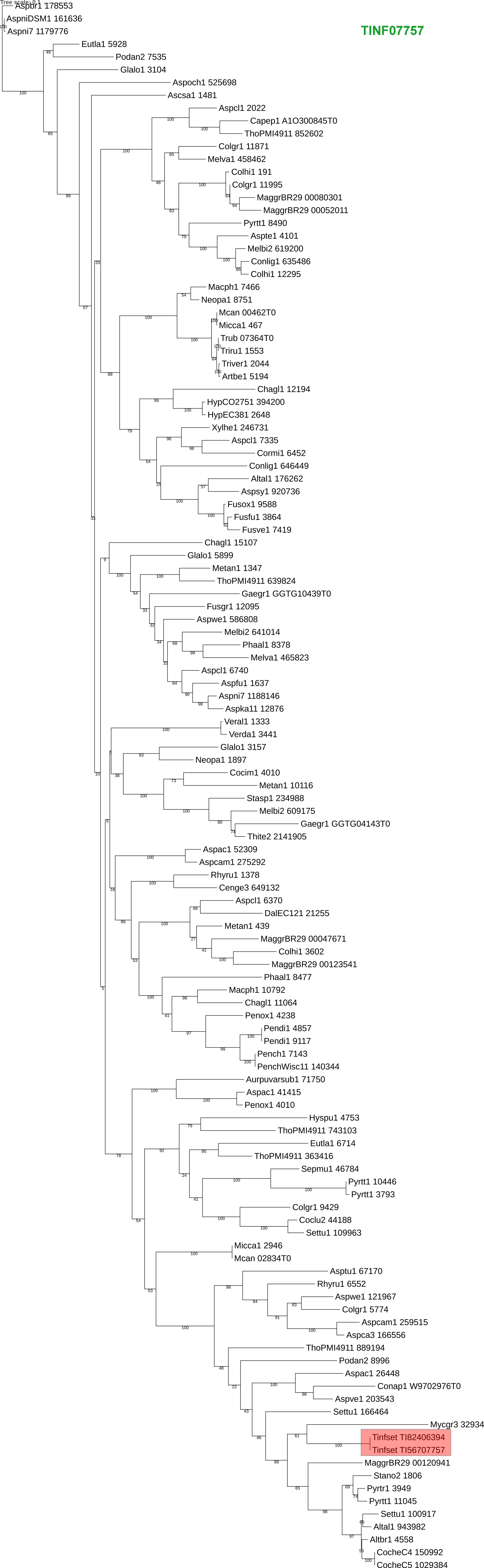

TINF07757
Tree scale: 0.1
Aspbr1 178553
AspniDSM1 161636
Aspni7 1179776
Eutla1 5928
Podan2 7535
Glalo1 3104
Aspoch1 525698
Ascsa1 1481
Aspcl1 2022
Capep1 A1O300845T0
ThoPMI4911 852602
Colgr1 11871
Melva1 458462
Colhi1 191
Colgr1 11995
MaggrBR29 00080301
MaggrBR29 00052011
Pyrtt1 8490
Aspte1 4101
Melbi2 619200
Conlig1 635486
Colhi1 12295
Macph1 7466
Neopa1 8751
Mcan 00462T0
Micca1 467
Trub 07364T0
Triru1 1553
Triver1 2044
Artbe1 5194
Chagl1 12194
HypCO2751 394200
HypEC381 2648
Xylhe1 246731
Aspcl1 7335
Cormi1 6452
Conlig1 646449
Altal1 176262
Aspsy1 920736
Fusox1 9588
Fusfu1 3864
Fusve1 7419
Chagl1 15107
Glalo1 5899
Metan1 1347
ThoPMI4911 639824
Gaegr1 GGTG10439T0
Fusgr1 12095
Aspwe1 586808
Melbi2 641014
Phaal1 8378
Melva1 465823
Aspcl1 6740
Aspfu1 1637
Aspni7 1188146
Aspka11 12876
Veral1 1333
Verda1 3441
Glalo1 3157
Neopa1 1897
Cocim1 4010
Metan1 10116
Stasp1 234988
Melbi2 609175
Gaegr1 GGTG04143T0
Thite2 2141905
Aspac1 52309
Aspcam1 275292
Rhyru1 1378
Cenge3 649132
Aspcl1 6370
DalEC121 21255
Metan1 439
MaggrBR29 00047671
Colhi1 3602
MaggrBR29 00123541
Phaal1 8477
Macph1 10792
Chagl1 11064
Penox1 4238
Pendi1 4857
Pendi1 9117
Pench1 7143
PenchWisc11 140344
Aurpuvarsub1 71750
Aspac1 41415
Penox1 4010
Hyspu1 4753
ThoPMI4911 743103
Eutla1 6714
ThoPMI4911 363416
Sepmu1 46784
Pyrtt1 10446
Pyrtt1 3793
Colgr1 9429
Coclu2 44188
Settu1 109963
Micca1 2946
Mcan 02834T0
Asptu1 67170
Rhyru1 6552
Aspwe1 121967
Colgr1 5774
Aspcam1 259515
Aspca3 166556
ThoPMI4911 889194
Podan2 8996
Aspac1 26448
Conap1 W9702976T0
Aspve1 203543
Settu1 166464
Mycgr3 32934
Tinfset TI82406394
Tinfset TI56707757
MaggrBR29 00120941
Stano2 1806
Pyrtr1 3949
Pyrtt1 11045
Settu1 100917
Altal1 943982
Altbr1 4558
CocheC4 150992
CocheC5 1029384

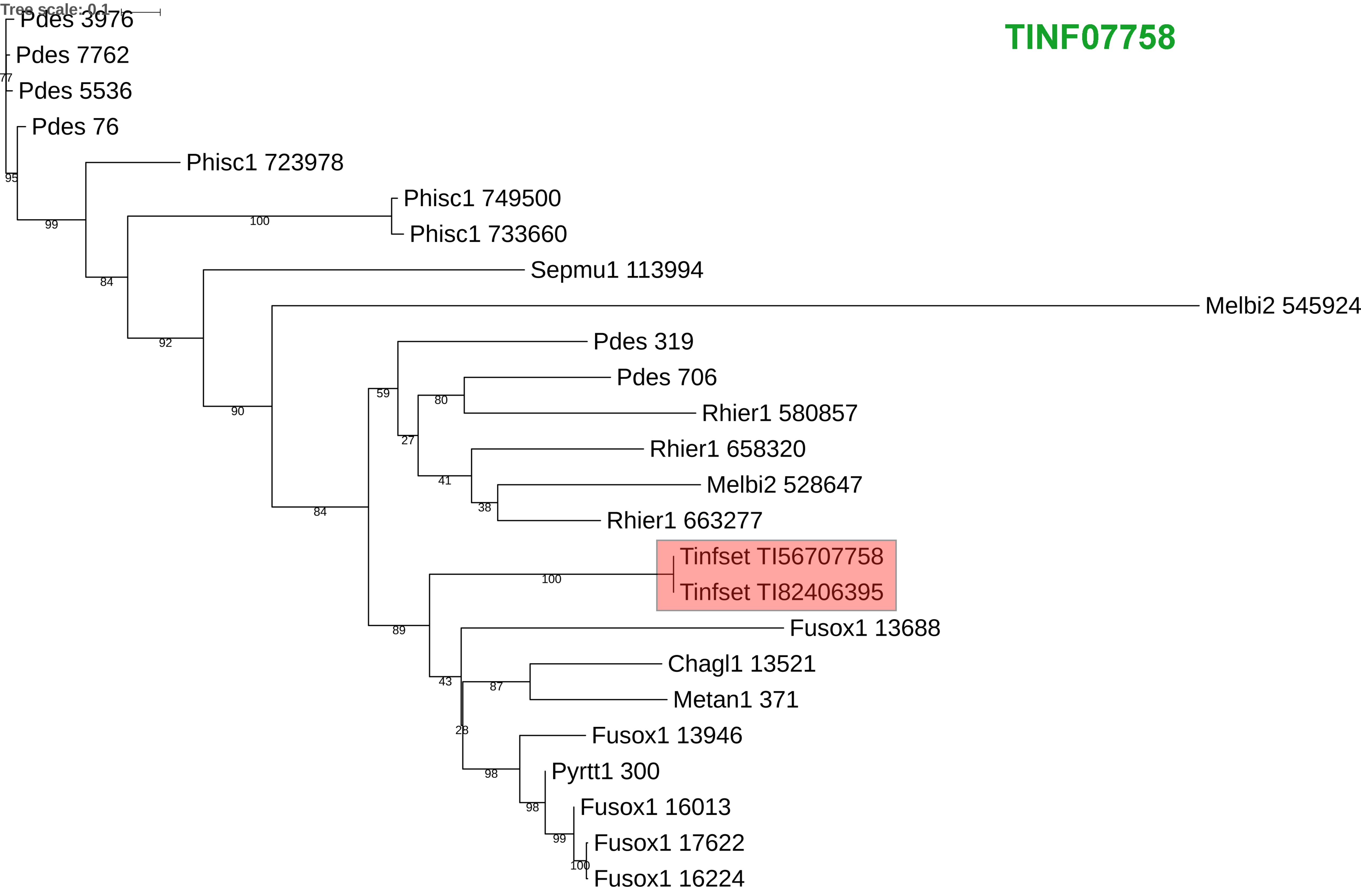

Tree scale: 0.1
TINF07758
Pdes 3976
Pdes 7762
Pdes 5536
Pdes 76
Phisc1 723978
Phisc1 749500
Phisc1 733660
Sepmu1 113994
Melbi2 545924
Pdes 319
Pdes 706
Rhier1 580857
Rhier1 658320
Melbi2 528647
Rhier1 663277
Tinfset TI56707758
Tinfset TI82406395
Fusox1 13688
Chagl1 13521
Metan1 371
Fusox1 13946
Pyrtt1 300
Fusox1 16013
Fusox1 17622
Fusox1 16224

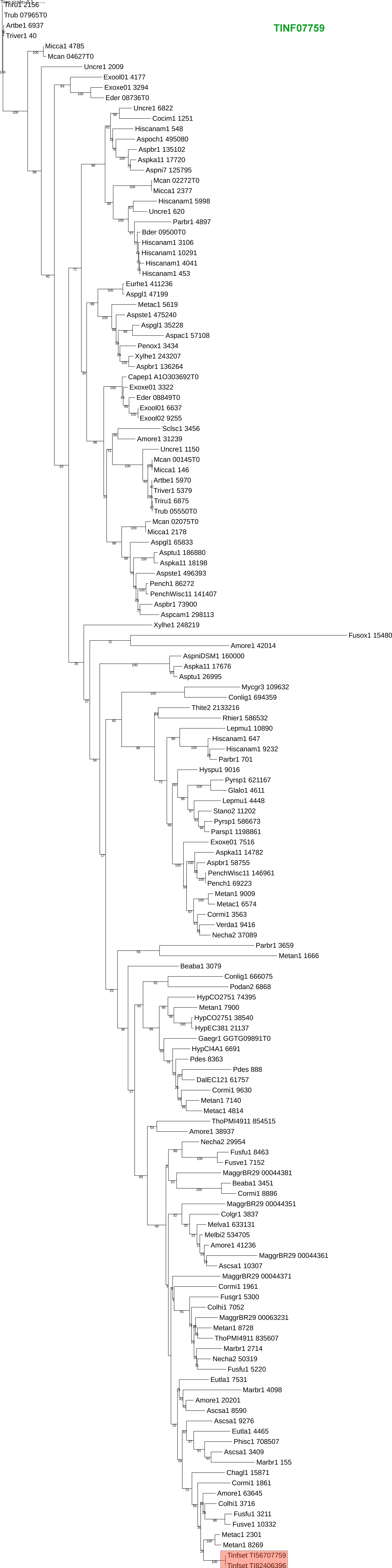
TINF07759
Tree scale: 0.1
Triru1 2156
Trub 07965T0
Artbe1 6937
Triver1 40
Micca1 4785
Mcan 04627T0
Uncre1 2009
Exool01 4177
Exoxe01 3294
Eder 08736T0
Uncre1 6822
Cocim1 1251
Hiscanam1 548
Aspoch1 495080
Aspbr1 135102
Aspka11 17720
Aspni7 125795
Mcan 02272T0
Micca1 2377
Hiscanam1 5998
Uncre1 620
Parbr1 4897
Bder 09500T0
Hiscanam1 3106
Hiscanam1 10291
Hiscanam1 4041
Hiscanam1 453
Eurhe1 411236
Aspgl1 47199
Metac1 5619
Aspste1 475240
Aspgl1 35228
Aspac1 57108
Penox1 3434
Xylhe1 243207
Aspbr1 136264
Capep1 A1O303692T0
Exoxe01 3322
Eder 08849T0
Exool01 6637
Exool02 9255
Sclsc1 3456
Amore1 31239
Uncre1 1150
Mcan 00145T0
Micca1 146
Artbe1 5970
Triver1 5379
Triru1 6875
Trub 05550T0
Mcan 02075T0
Micca1 2178
Aspgl1 65833
Asptu1 186880
Aspka11 18198
Aspste1 496393
Pench1 86272
PenchWisc11 141407
Aspbr1 73900
Aspcam1 298113
Xylhe1 248219
Fusox1 15480
Amore1 42014
AspniDSM1 160000
Aspka11 17676
Asptu1 26995
Mycgr3 109632
Conlig1 694359
Thite2 2133216
Rhier1 586532
Lepmu1 10890
Hiscanam1 647
Hiscanam1 9232
Parbr1 701
Hyspu1 9016
Pyrsp1 621167
Glalo1 4611
Lepmu1 4448
Stano2 11202
Pyrsp1 586673
Parsp1 1198861
Exoxe01 7516
Aspka11 14782
Aspbr1 58755
PenchWisc11 146961
Pench1 69223
Metan1 9009
Metac1 6574
Cormi1 3563
Verda1 9416
Necha2 37089
Parbr1 3659
Metan1 1666
Beaba1 3079
Conlig1 666075
Podan2 6868
HypCO2751 74395
Metan1 7900
HypCO2751 38540
HypEC381 21137
Gaegr1 GGTG09891T0
HypCl4A1 6691
Pdes 8363
Pdes 888
DalEC121 61757
Cormi1 9630
Metan1 7140
Metac1 4814
ThoPMI4911 854515
Amore1 38937
Necha2 29954
Fusfu1 8463
Fusve1 7152
MaggrBR29 00044381
Beaba1 3451
Cormi1 8886
MaggrBR29 00044351
Colgr1 3837
Melva1 633131
Melbi2 534705
Amore1 41236
MaggrBR29 00044361
Ascsa1 10307
MaggrBR29 00044371
Cormi1 1961
Fusgr1 5300
Colhi1 7052
MaggrBR29 00063231
Metan1 8728
ThoPMI4911 835607
Marbr1 2714
Necha2 50319
Fusfu1 5220
Eutla1 7531
Marbr1 4098
Amore1 20201
Ascsa1 8590
Ascsa1 9276
Eutla1 4465
Phisc1 708507
Ascsa1 3409
Marbr1 155
Chagl1 15871
Cormi1 1861
Amore1 63645
Colhi1 3716
Fusfu1 3211
Fusve1 10332
Metac1 2301
Metan1 8269
Tinfset TI56707759
Tinfset TI82406396

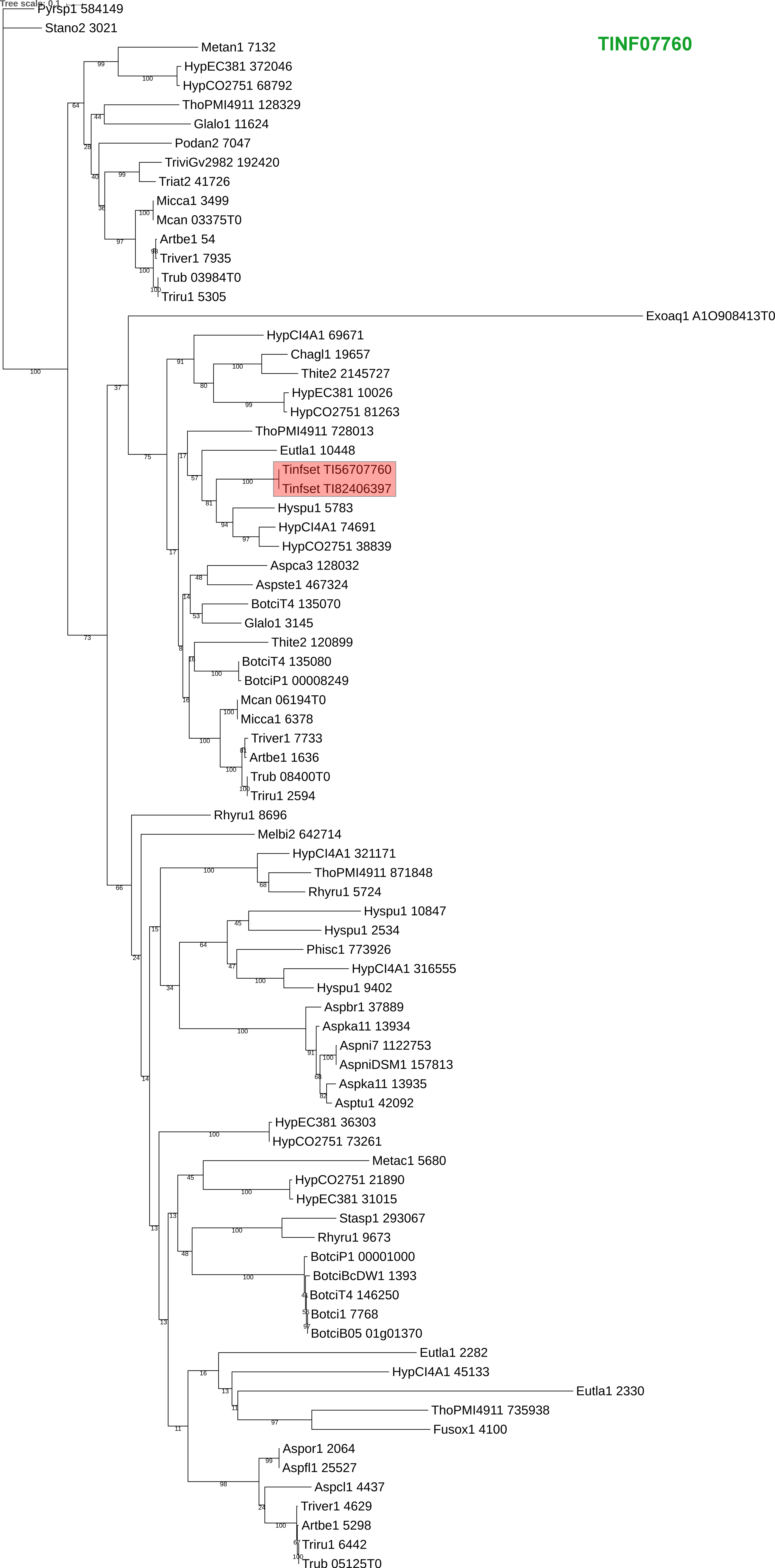
Tree scale: 0.1
TINF07760
Pyrsp1 584149
Stano2 3021
Metan1 7132
HypEC381 372046
HypCO2751 68792
ThoPMI4911 128329
Glalo1 11624
Podan2 7047
TriviGv2982 192420
Triat2 41726
Micca1 3499
Mcan 03375T0
Artbe1 54
Triver1 7935
Trub 03984T0
Triru1 5305
Exoaq1 A1O908413T0
HypCI4A1 69671
Chagl1 19657
Thite2 2145727
HypEC381 10026
HypCO2751 81263
ThoPMI4911 728013
Eutla1 10448
Tinfset TI56707760
Tinfset TI82406397
Hyspu1 5783
HypCI4A1 74691
HypCO2751 38839
Aspca3 128032
Aspste1 467324
BotciT4 135070
Glalo1 3145
Thite2 120899
BotciT4 135080
BotciP1 00008249
Mcan 06194T0
Micca1 6378
Triver1 7733
Artbe1 1636
Trub 08400T0
Triru1 2594
Rhyru1 8696
Melbi2 642714
HypCI4A1 321171
ThoPMI4911 871848
Rhyru1 5724
Hyspu1 10847
Hyspu1 2534
Phisc1 773926
HypCI4A1 316555
Hyspu1 9402
Aspbr1 37889
Aspka11 13934
Aspni7 1122753
AspniDSM1 157813
Aspka11 13935
Asptu1 42092
HypEC381 36303
HypCO2751 73261
Metac1 5680
HypCO2751 21890
HypEC381 31015
Stasp1 293067
Rhyru1 9673
BotciP1 00001000
BotciBcDW1 1393
BotciT4 146250
Botci1 7768
BotciB05 01g01370
Eutla1 2282
HypCI4A1 45133
Eutla1 2330
ThoPMI4911 735938
Fusox1 4100
Aspor1 2064
Aspfl1 25527
Aspcl1 4437
Triver1 4629
Artbe1 5298
Triru1 6442
Trub 05125T0

TINF07761

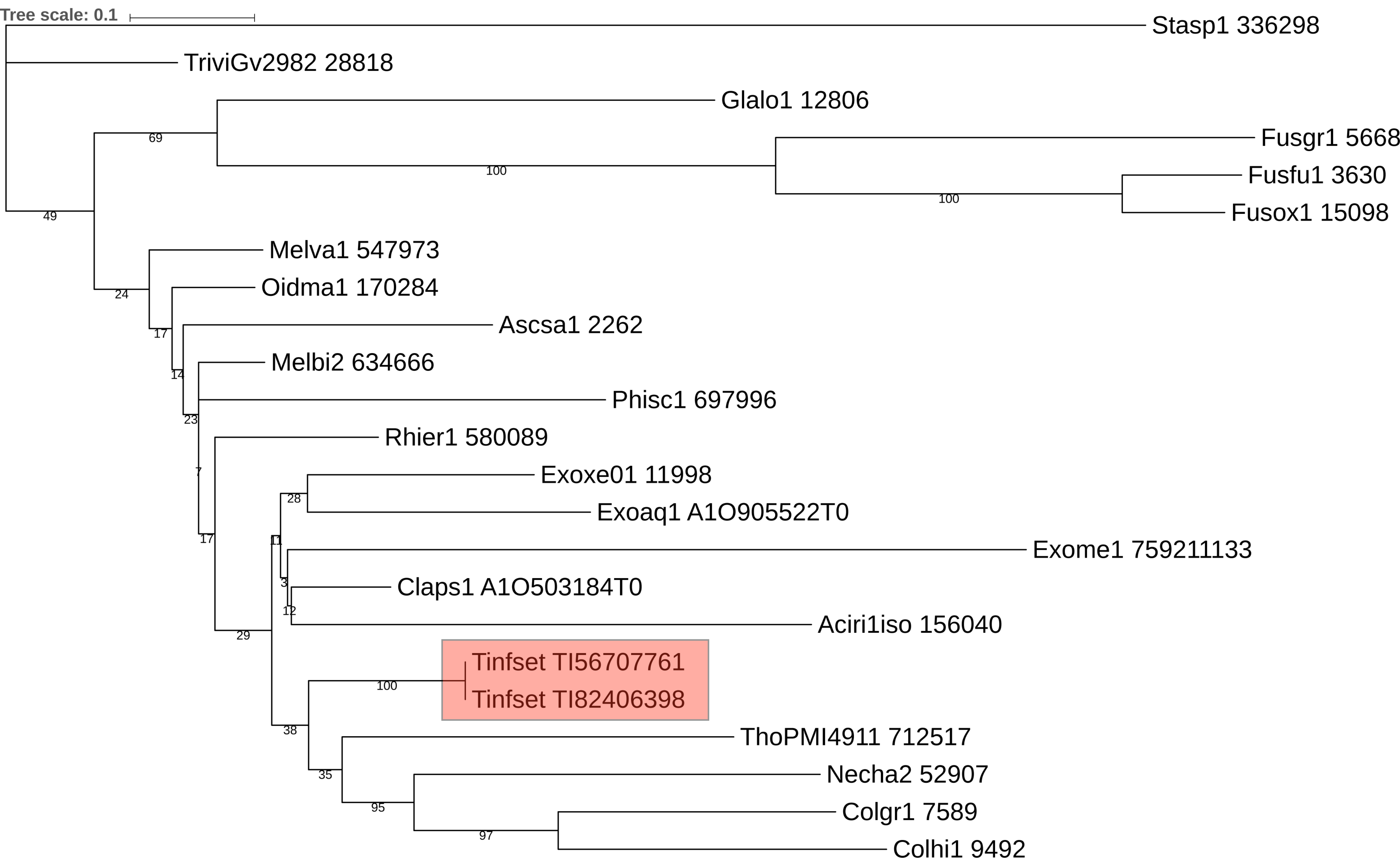

Tree scale: 0.1
Stasp1 336298
TriviGv2982 28818
Glalo1 12806
Fusgr1 5668
Fusfu1 3630
Fusox1 15098
Melva1 547973
Oidma1 170284
Ascsa1 2262
Melbi2 634666
Phisc1 697996
Rhier1 580089
Exoxe01 11998
Exoaq1 A1O905522T0
Exome1 759211133
Claps1 A1O503184T0
Aciri1iso 156040
Tinfset TI56707761
Tinfset TI82406398
ThoPMI4911 712517
Necha2 52907
Colgr1 7589
Colhi1 9492
69
100
100
49
24
17
14
23
7
17
28
11
3
12
29
100
38
35
95
97

Key to Fungal Taxa

Abbreviation	Fungal species
Aciri1iso	Acidomyces richmondensis BFW
Altal1	Alternaria alternata SRC1lrK2f
Altbr1	Alternaria brassicicola
Amore1	Amorphotheca resinae
Artbe1	Arthroderma benhamiae CBS 112371
Artol1	Arthrobotrys oligospora ATCC 24927
Ascsa1	Ascocoryne sarcoides NRRL50072
Aspac1	Aspergillus aculeatus ATCC16872
Aspbr1	Aspergillus brasiliensis
Aspca3	Aspergillus carbonarius
Aspcam1	Aspergillus campestris IBT 28561
Aspcl1	Aspergillus clavatus NRRL 1 from AspGD
Aspfl1	Aspergillus flavus NRRL3357
Aspfu1	Aspergillus fumigatus Af293 from AspGD
AspfuA11631	Aspergillus_fumigatus_A1163
Aspgl1	Aspergillus glaucus
Aspka11	Aspergillus kawachii IFO 4308
Aspni7	Aspergillus niger ATCC 1015
Aspnid1	Aspergillus nidulans from AspGD
AspniDSM1	Aspergillus niger CBS 513.88
Aspnov1	Aspergillus novofumigatus IBT 16806
Aspoch1	Aspergillus ochraceoroseus IBT 24754
Aspor1	Aspergillus oryzae RIB40
Aspste1	Aspergillus steynii IBT 23096
Aspsy1	Aspergillus sydowii
Aspte1	Aspergillus_terreus_NIH_2624
Asptu1	Aspergillus tubingensis
Aspve1	Aspergillus versicolor
Aspwe1	Aspergillus_wentii
Aspzo1	Aspergillus zonatus
Aurpuvarmel1	Aureobasidium pullulans var. melanogenum CBS 110374
Aurpuvarnam1	Aureobasidium pullulans var. namibiae CBS 147.97
Aurpuvarpul1	Aureobasidium pullulans var. pullulans EXF-150
Aurpuvarsub1	Aureobasidium pullulans var. subglaciale EXF-2481
Bauco1	Baudoinia compniacensis UAMH 10762

Bder	Blastomyces dermatitidis
Beaba1	Beauveria bassiana ARSEF 2860
Blugr1	Blumeria graminis f.sp. hordei DH14
Botci1	Botrytis_cinerea
BotciB05	Botrytis_cinerea
BotciBcDW1	Botrytis_cinerea
BotciP1	Botrytis_cinerea
BotciT4	Botrytis_cinerea
Capco1	Capronia coronata cbs 617.96
Capep1	Capronia epimyces cbs 606.96
Cenge3	Cenococcum geophilum 1.58
Chagl1	Chaetomium globosum v1.0
Claca1	Cladophialophora carrionii CBS 160.54
Clafu1	Cladosporium fulvum
Claps1	Cladophialophora psammophila CBS 110553
Claye1	Cladophialophora yegresii cbs 114405
Cocca1	Cochliobolus carbonum 26-R-13 v1.0
CocheC4	Cochliobolus heterostrophus C4
CocheC5	Cochliobolus heterostrophus C5
Cocim1	Coccidioides immitis RS
Coclu2	Cochliobolus lunatus m118
Cocmi1	Cochliobolus miyabeanus ATCC 44560
Cocsa1	Cochliobolus sativus ND90Pr
Cocvi1	Cochliobolus victoriae FI3
Colgr1	Colletotrichum graminicola M1.001
Colhi1	Colletotrichum higginsianum IMI 349063
Conap1	Coniosporium apollinis cbs 100218
Conlig1	Coniochaeta ligniaria NRRL 30616 v1.0
Cormi1	Cordyceps militaris CM01
DalEC121	Daldinia eschscholzii EC12
Dotse1	Dothistroma septosporum NZE10
Eder	Exophiala dermatitidis UT8656
Eurhe1	Eurotium rubrum
Eutla1	Eutypa lata UCREL1
Exoaq1	Exophiala aquamarina cbs 119918
Exome1	Exophiala mesophila CBS40295
Exool01	Exophiala oligosperma CBS72588
Exool02	Exophiala oligosperma CBS 72.588
Exoxe01	Exophiala xenobiotica
Fusfu1	Fusarium fujikuroi IMI 58289
Fusgr1	Fusarium graminearum

Fusox1	Fusarium oxysporum f. sp. lycopersici strain 4287
Fusve1	Fusarium verticillioides 7600
Gaegr1	Gaeumannomyces graminis var. tritici R3-111a-1
Glalo1	Glarea lozoyensis ATCC 20868
Grocl1	Grosmannia_clavigera_kw1407
Hiscanam1	Histoplasma capsulatum nam1
HypCI4A1	Hypoxylon sp. CI-4A
HypCO2751	Hypoxylon sp. CO27-5 v1.0
HypEC381	Hypoxylon sp. EC38 v3.0
Hyspu1	Hysterium pulicare
Lepmu1	Leptosphaeria maculans
Leppa1	Lepidopterella_palustris
Macph1	Macrophomina phaseolina MS6
MaggrBR29	Magnaporthe grisea BR29
Marbr1	Marssonina brunnea f. sp. multigermtubi MB m1
Mcan	Microsporum canis CBS113480
Melbi2	Meliniomyces bicolor E
Melva1	Meliniomyces variabilis F
Metac1	Metarhizium acridum CQMa 102
Metan1	Metarhizium robertsii ARSEF 23
Micca1	Microsporum canis CBS 113480
Monha1	Monacrosporium haptotylum CBS 200.50
Mycgr3	Mycosphaerella graminicola
Necha2	Nectria haematococca
Neofi1	Neosartorya fischeri NRRL 181
Neopa1	Neofusicoccum parvum UCRNP2
Neucr2	Neurospora crassa OR74A v2.0
Neucrtrp31	Neurospora crassa FGSC 73 trp-3 v1.0
Neutemata1	Neurospora tetrasperma FGSC 2509 mat a v1.0
NeutematA2	Neurospora tetrasperma FGSC 2508 mat A v2.0
Oidma1	Oidiodendron maius Zn
Ophpc1	Ophiostoma piceae UAMH 11346
Parbr1	Paracoccidioides_brasiliensis_Pb03
Parsp1	Paraconiothyrium sporulosum AP3s5-JAC2a
Pdes	Pseudogymnoascus destructans 20631-21
Pench1	Penicillium chrysogenum v1.0
PenchWisc11	Penicillium chrysogenum Wisconsin 54-1255
Pendi1	Penicillium digitatum PHI26
Penox1	Penicillium oxalicum 114-2
Phaal1	Phaeoacremonium aleophilum UCRPA7
Phieu1	Phialophora europaea cbs 101466

Phisc1	Phialocephala scopiformis 5WS22E1
Podan2	Podospora anserina S mat+
Pyrco1	Pyronema confluens CBS100304
Pyrsp1	Pyrenochaeta sp. DS3sAY3a
Pyrtr1	Pyrenophora tritici-repentis
Pyrtt1	Pyrenophora teres f. teres
Rhier1	Rhizoscyphus ericae UAMH 7357
Rhyru1	Rhytidhysteron rufulum
Sclsc1	Sclerotinia_sclerotiorum
Sepmu1	Septoria musiva SO2202
Settu1	Setosphaeria turcica Et28A
Stano2	Stagonospora nodorum SN15
Stasp1	Stagonospora sp. SRC1lsM3a
Thite2	Thielavia terrestris v2.0
ThoPMI4911	Thozetella sp. PMI 491
Tinfset	Tolypocladium inflatum multiple
Triat2	Trichoderma_atroviride
Trire2	Trichoderma reesei
TrireRUTC301	Trichoderma reesei RUT C-30
Triru1	Trichophyton_rubrum_CBS_118892
Triver1	Trichophyton verrucosum HKI 0517
TriviGv2982	Trichoderma virens Gv29-8
Trub	Trichophyton rubrum CBS118892
Tubme1	Tuber melanosporum from Genoscope
Uncre1	Uncinocarpus reesii 1704
Xylhe1	Xylona heveae TC161
Zymar1	Zymoseptoria ardabiliae STIR04 1.1.1
Zymps1	Zymoseptoria pseudotritici STIR04 2.2.1
