# Supplementary material for: Chromosome rearrangements shape the diversification of secondary metabolism in the cyclosporin producing fungus Tolypocladium inflatum
Source: BMC Genomics. 2019 Feb 7;20:120. doi: 10.1186/s12864-018-5399-x (PMC6367777; doi:10.1186/s12864-018-5399-x)

Figure S13 Mauve alignments of other clusters: conserved across all strains: A) Fumonisin-like cluster and B) Stipitatic acid-like cluster. Red shading shows conserved syntenic regions inside each SMBC.

A. Fumonisin-like Cluster - Chromosome 3 cluster 19

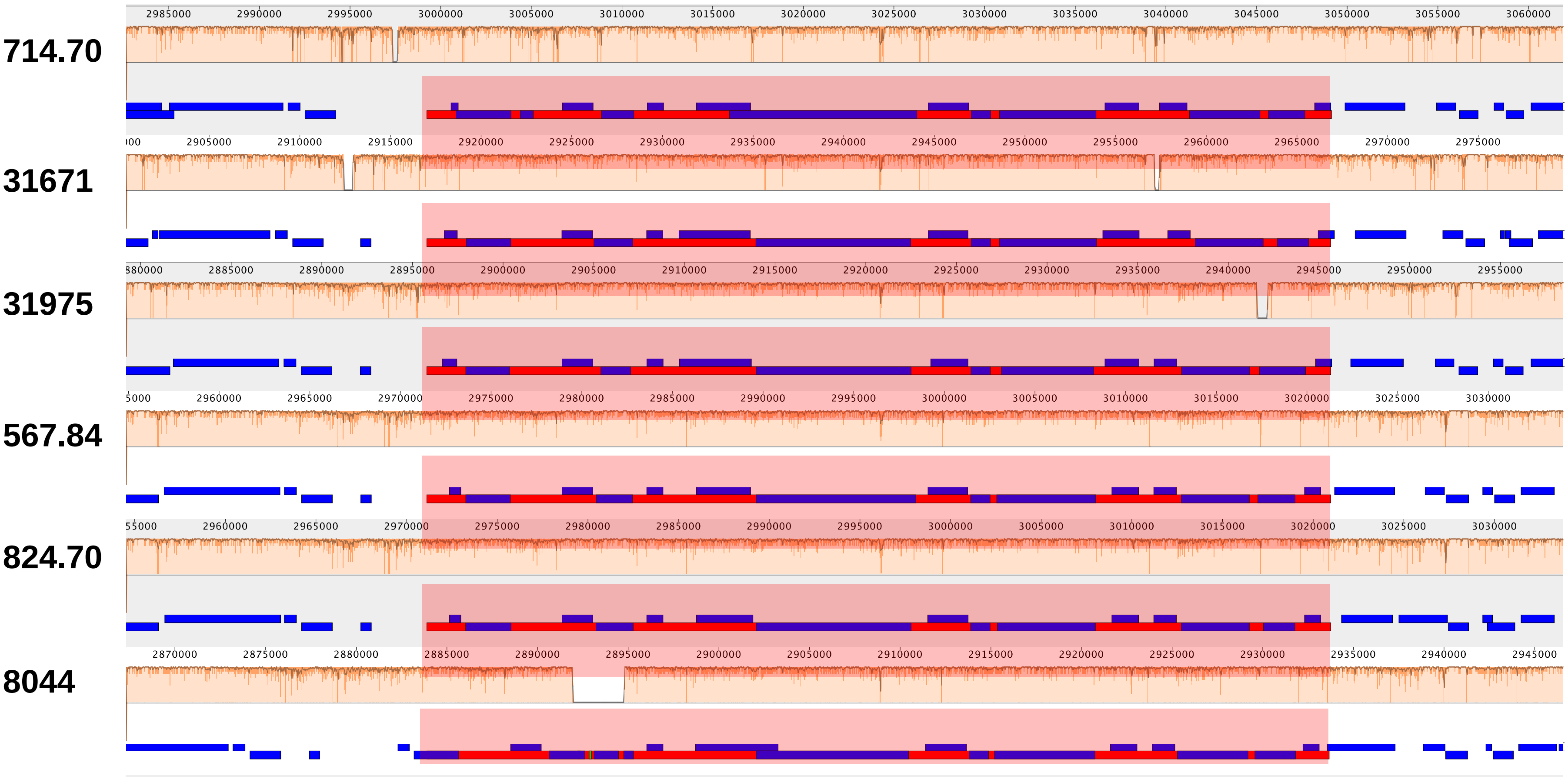

B. Stipitatic Acid-like Cluster - Chromosome 4 cluster 25

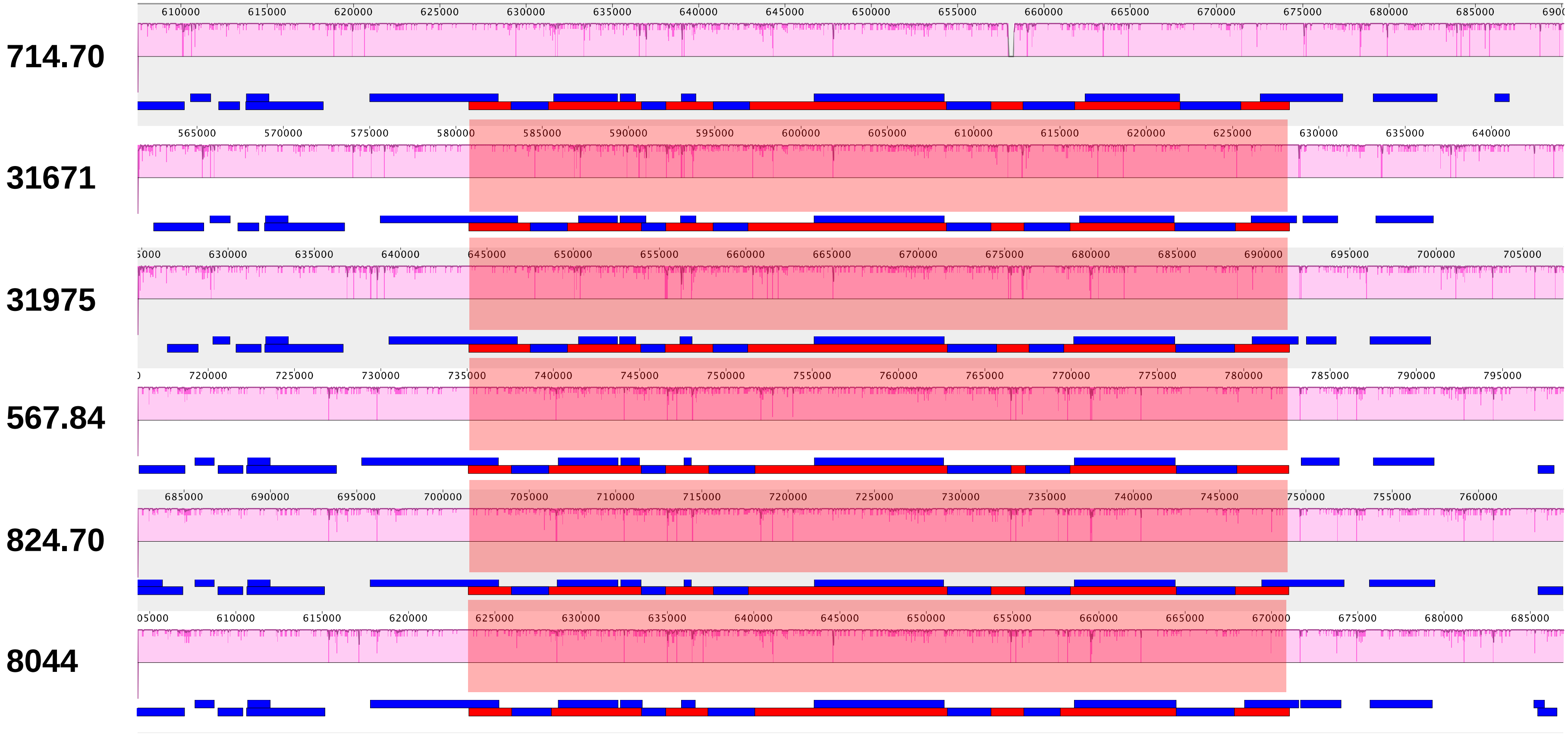

Supplement: Supplementary file 21 — Figure S13. Mauve alignments of conserved SMBGCs. (PDF 2.19 Mb) [file 12864_2018_5399_MOESM21_ESM.pdf]
